# A Sample-Centric and Knowledge-Driven Computational Framework for Natural Products Drug Discovery

**DOI:** 10.1021/acscentsci.3c00800

**Published:** 2024-02-20

**Authors:** Arnaud Gaudry, Marco Pagni, Florence Mehl, Sébastien Moretti, Luis-Manuel Quiros-Guerrero, Luca Cappelletti, Adriano Rutz, Marcel Kaiser, Laurence Marcourt, Emerson Ferreira Queiroz, Jean-Robert Ioset, Antonio Grondin, Bruno David, Jean-Luc Wolfender, Pierre-Marie Allard

**Affiliations:** † Institute of Pharmaceutical Sciences of Western Switzerland, University of Geneva, 1211 Geneva 4, Switzerland; ‡ School of Pharmaceutical Sciences, University of Geneva, 1211 Geneva 4, Switzerland; § Vital-IT, SIB Swiss Institute of Bioinformatics, 1015 Lausanne, Switzerland; ∥ Department of Medical and Parasitology and Infection Biology, Swiss Tropical and Public Health Institute, 4123 Allschwil, Switzerland; ⊥ Faculty of Science, University of Basel, 4002 Basel, Switzerland; # Drugs for Neglected Diseases Initiative (DNDi), 1202 Geneva, Switzerland; □ Green Mission Pierre Fabre, Institut de Recherche Pierre Fabre, 31562 Toulouse, France; ◆ Department of Biology, University of Fribourg, 1700 Fribourg, Switzerland

## Abstract

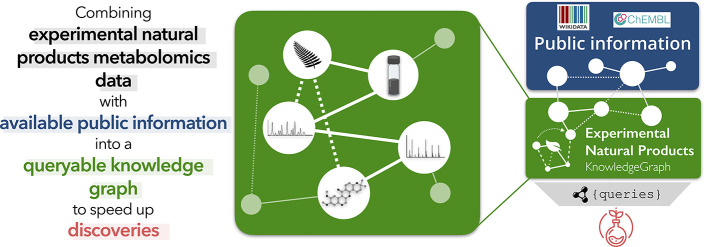

The ENPKG framework organizes large heterogeneous metabolomics data sets as a knowledge graph, offering exciting opportunities for drug discovery and chemodiversity characterization.

## 1 Introduction

Natural products (NPs) possess structural properties that confer them a privileged status in drug discovery. However, such properties also entail significant challenges for NPs chemists.^1,2^ NPs are characterized by more sp^3^ carbons, chiral centers, or oxygen atoms than synthetic compounds, and while explaining their relevance to biological targets and higher hit rates in drug discovery screening programs, this complexity also explains the difficulties encountered in their chemical synthesis.^3−7^ At the natural extracts (NEs) level, the complexity arises from the vast array of structurally diverse compounds, each at varying concentrations, constituting the NE mixture. This complexity poses a significant challenge in determining the precise composition of NEs.

At present, the main instrumental setup used to characterize complex NEs is ultrahigh-performance liquid chromatography coupled with tandem high-resolution mass spectrometry (UHPLC-HRMS^2^).^8^ The resulting data are usually processed to obtain a list of LC-MS features—a chromatographic peak with a given *m*/*z*, retention time (RT), area/intensity—and their associated MS^2^ spectrum. The features from all considered samples are then classically aligned based on their RT and *m*/*z* to obtain a feature quantification table of dimensions *N* × *M* (*N* represents the number of samples and *M* the number of features) and their associated fragmentation spectra. The latter can be used to perform structural annotation, and the quantitative, spectral, and structural data can then be integrated and visualized through feature-based molecular networking (FBMN).^9−11^ FBMN is a technique used to organize features’ fragmentation spectra in spectral similarity clusters for visualization and analysis. In addition to the quantification table, different extracts’ metadata, such as taxonomic position of the biosource or bioactivity, can be integrated into the FBMN to enhance its analysis and interpretation. Multi-informative FBMN can then help to highlight groups of features specific to active extracts or a given taxon.^12^

However, the feature alignment stage is problematic for extensive UHPLC-MS^2^ metabolomics research projects encompassing a large number of samples. First, since metabolite profiling data are typically recorded in independent batches, the resulting data are prone to batch effects due to the variation in both LC and MS dimensions, thus preventing a proper alignment.^13,14^ Second, the inclusion of novel samples to previously analyzed data sets requires the recomputation of alignment and postalignment processing steps such as structural annotation or FBMN.^11^ This drawback is particularly problematic for large data sets (hundreds to thousands of samples), for which these analyses require a large amount of time and computational resources. Such classical approaches—hereafter qualified as *data set-*centric—result in the compartmentalization of data and information into hermetic project-related silos, triggering a need for new methods to exploit the data. To this end, we recently developed the MEMO approach to compare chemodiverse samples based on their MS^2^ fingerprints without relying on an RT-based alignment.^14^ In this work, we push this concept further and propose shifting from a data set-centric to a sample-centric approach, enabling alignment not only through spectral data but also via related chemical information or any relevant metadata. Each sample is considered individually for taxonomic metadata standardization, feature detection, structural annotation, and FBMN. The resulting standardized data and information from all considered samples are then integrated into a single knowledge graph (KG). This sample-centric approach, implemented in the Experimental Natural Products Knowledge Graph (ENPKG) framework, is described hereafter.

A KG can store complex and heterogeneous data, which can be organized and interpreted using the graph topology. A format used to build a KG is the resource description framework (RDF), a standard graph model for structured and semistructured data interchange on the Web.^15^ In a KG, data can thus be stored as RDF *subject*–*predicate*–*object* triples, such as, for example, *molecule A* (subject) is *found in* (predicate) *species X* (object). These graphs can be queried using the SPARQL Protocol and RDF Query Language (SPARQL), a language designed to query and extract information from RDF databases.^16^ It is a powerful language facilitating precise and targeted retrieval of specific data elements from large and structured data sets, enabling the efficient exploration and analysis of complex information networks. Because the RDF is a W3C standard, KGs are interoperable, and it is possible to elaborate federated queries over multiple end points to retrieve and link data from different RDF databases (DBs). An example of a knowledge base is Wikidata, which contains more than 1 billion statements. It has a wide range of applications—from bibliographic information management^17^ to biomedical data integration^18^—and is currently used by the life science community to disseminate FAIR (findability, accessibility, interoperability, and reusability) data and knowledge.^19,20^ In NPs research, Wikidata is used as a dissemination and curation platform for documented structure–organism pairs by the LOTUS initiative.^19^ KGs have also been used in drug discovery, for example, to predict adverse drug reactions or help find the best candidates for drug repurposing.^21,22^

Thanks to their versatility, KGs can integrate standardized experimental data *and* public knowledge. Integrating scientific knowledge early into the data analysis workflow can enhance the discovery process by helping researchers interpret and contextualize the experimental results. Santos et al. published a brilliant example in this direction—the clinical knowledge graph—illustrating how integrating experimental clinical proteomics data *and* biomedical knowledge bases into a unique KG effectively enhances data analysis.^23^ In the context of drug discovery, the graph generated by the ENPKG framework allows to efficiently convert the large amount of information obtained in natural extracts screening campaigns into discoveries. To confirm this, we showcased its implementation to leverage the results obtained from the phenotypic screening of 1,600 plant extracts against three trypanosomatids: *Leishmania donovani* (Q1950752), *Trypanosoma cruzi* (Q150162), and *T. brucei rhodesiense* (Q30216064). To illustrate the potential of our approach to organize and interrogate heterogeneous data sets, we have further formatted and integrated a publicly available metabolomics data set of 337 Korean medicinal plants acquired under different experimental conditions in the ENPKG.^24^ Here, we will first detail the workflow structure and its technical aspects and then show how the KG structure can help answer research questions and lead to the identification of new anti-*T. cruzi* and anti-*L. donovani* compounds. We finally discuss the current limitations of the workflow and future improvements.

## 2 Results and Discussion

### 2.1 Conceptual Overview of the ENPKG Workflow

Modern NPs metabolomics workflows produce large amounts of data and information. Each natural extract is characterized by its metadata—taxonomy of the organism, organ, or part studied, type of extract, bioactivity, etc.—and hundreds to thousands of metabolites characterized by their LC-MS features, chemical classes, or structural annotations. Molecular networking (MN) has appeared in recent years as a revolutionary solution to organize and explore large spectral data sets.^10^ While MN offers an efficient way to display spectral similarities, large data sets are still challenging to explore using visualization software only (*e.g*., Cytoscape).^25^ Limitations in data exploration are quickly reached when trying to map multiple layers of additional information (*e.g*., bioactivity results, taxonomical origins, chemical classes, etc.). In addition, there are currently no efficient solutions for the sharing, parallel exploration, and reuse of such large heterogeneous data sets.

**Figure 1:**
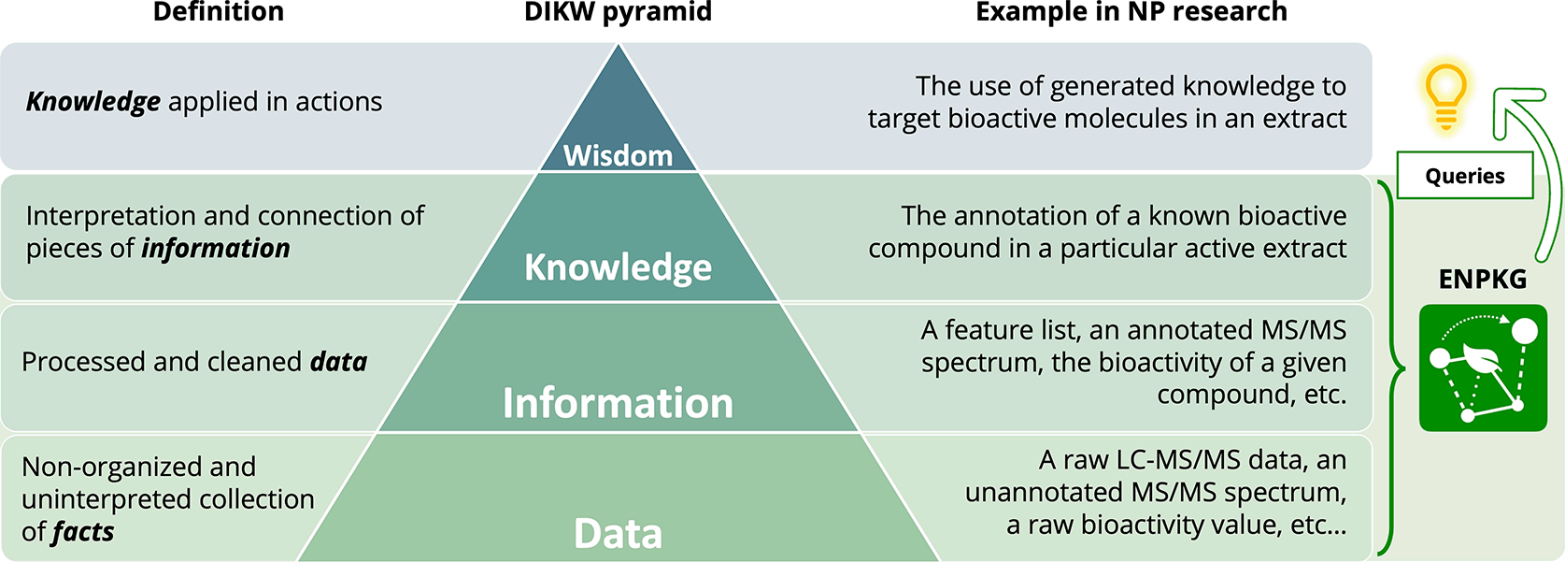
Conceptual overview of the Experimental Natural Products Knowledge Graph (ENPKG) using the Data, Information, Knowledge, and Wisdom (DIKW) pyramid. *Data*, such as raw LC-MS^2^, are automatically processed into *information* (a structural annotation, for example) in a sample-centric way. Data and information are then standardized and integrated into a unified knowledge graph (ENPKG) structure that allows the generation of knowledge by linking these pieces of data and information within and across samples and the publicly available knowledge through links to WD and ChEMBL, for example. The resulting ensemble can then serve to answer various questions through queries (e.g., via SPARQL) and paves the way for the implementation of automated reasoning mechanisms.

**Figure 2:**
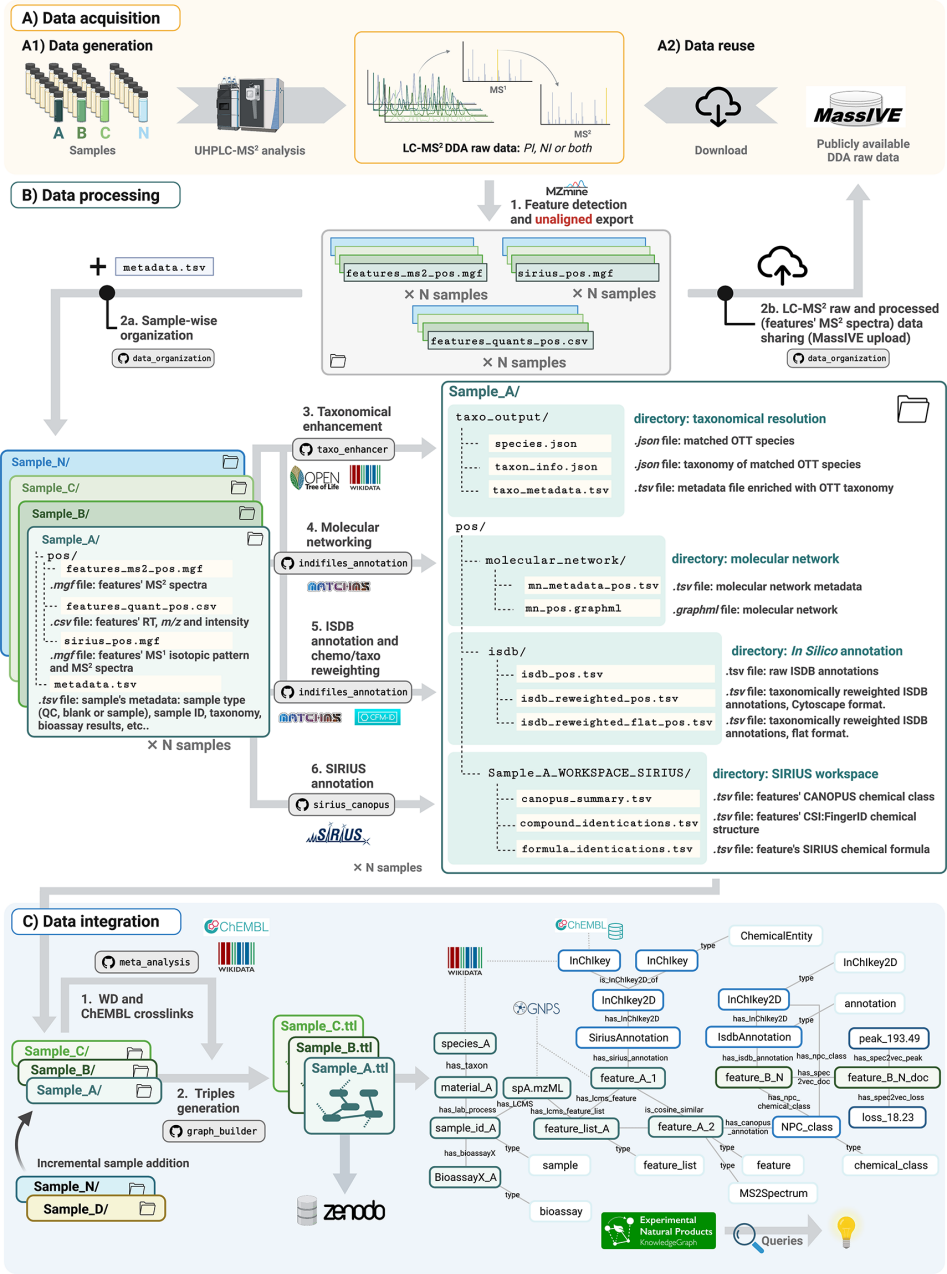
Experimental Natural Products Knowledge Graph (ENPKG) generation workflow. The ENPKG workflow follows three stages: Data acquisition (A), Data processing (B) and Data integration (C). There are two ways for (A) Data acquisition: (A1) Data generation: novel LC-MS^2^ data are directly added by the researcher. (A2) Data reuse: Ingestion of publicly available LC-MS^2^ data. Data obtained through A1 and A2 can be combined. (B) Data processing: The raw DDA data are (1) processed using MZmine feature finding, (2a) organized in a sample-wise directory architecture, and (2b) experimental raw data (.mzML) and LC-MS features’ spectra (.mgf) are uploaded on the GNPS MASSive repository. Then, for each sample, the following steps are performed (3) taxonomical metadata standardization and uniformization using Open Tree Taxonomy matching and Wikidata cross-links, (4) molecular networks generation via matchms, (5) structural annotation using ISDB matching coupled to taxonomical and chemical consistency reweighting, and (6) structural annotation using SIRIUS and CSI:FingerID and chemical class annotation using CANOPUS. Once the processing is done, the generated data, information, and knowledge (DIK) are integrated into a single KG. (C) Data integration: (1) First, the meta_analysis stage allows cross-linking of chemical structures to Wikidata and the addition of ChEMBL compounds with reported activity against a given target. (2) Then, the content of each directory is formatted as RDF triples (.ttl format) to generate a standalone KG for each sample. These individual KG can be shared on a repository such as Zenodo to enhance reusability and sharing. The overall KG can then be conveniently generated by combining the individual KGs. A detailed scheme of the KG is presented in Figure S1. PI: positive ionization, NI: negative ionization, KG: knowledge graph, WD: Wikidata.

The ENPKG workflow was designed to address these challenges. It aims to compile and streamline the various types of data and information generated by modern NPs metabolomics workflows into a unified KG, thereby effectively organizing and distilling the resulting knowledge. Thanks to reconciliation with external identifiers and semantic enrichment, newly generated data are matched and augmented with publicly shared knowledge. With this specific KG architecture, powerful interrogation strategies using the SPARQL, for example, can be harnessed to explore the gathered data, information, and knowledge. A conceptual overview of the ENPKG framework, using the Data, Information, Knowledge, and Wisdom (DIKW) pyramid is illustrated in Figure 1. Technical implementation and applications are detailed in the next sections and schematized in Figure 2.

### 2.2 Technical Overview of the ENPKG Workflow

The ENPKG workflow requires LC-HRMS^2^ data and associated metadata as input. LC-HRMS^2^ data can be obtained in two ways: directly generated by researchers (Figure 2 A1. Data generation) or by reusing published data sets (Figure 2 A2. Data reuse). In both cases, the data for each sample are processed to perform feature detection and export the resulting files (Figure 2B, step 1), here, using MZmine.^26^ The MS data files—the raw LC-HRMS^2^ data (.mzML format) and the features’ MS^2^ spectra (.mgf) files—are then uploaded to a MassIVE repository (Figure 2B, step 2b), hence allowing the complete MS data set to benefit from a Digital Object Identifier (DOI) and to attribute to each MSMS spectrum its corresponding universal spectrum identifier (USI).^27^ This mechanism allows efficient referencing, sharing, and observation of spectra, but also LC-HRMS^2^ profiles, through the GNPS dashboard, and in the end, facilitates their publication and reuse.^28,29^ In addition, we generated four links allowing to perform, for each individual spectrum of the ENPKG, a direct or analog spectral search against the GNPS libraries or the GNPS data index through the Fasst Search interface (https://fasst.gnps2.org/fastsearch/). In addition to the MS data, the minimal sufficient metadata should contain: the LC-HRMS^2^ data filename(s), a sample identifier (*sample_id*), the sample’s type (*QC*, *blank* or *sample*), a sample’s sources identifier (*source_id*), and the sample source’s taxonomical denomination (*source_taxon*) [see https://github.com/enpkg/enpkg_full/tree/main/01_enpkg_data_organization for details]. All previously described files are then organized in a sample-centered directory architecture—i.e., one sample corresponds to one directory (Figure 2B, step 2a) —before further processing.

The data present in each directory are then processed as follows: (1) taxonomical standardization of the samples’ provided biosource using OpenTree and OTT, and reconciliation with external identifiers in Wikidata,^30,31^ (2) a local implementation of FBMN^11^ using the matchms package,^32^ (3) chemical structure annotation using an *in silico* database of natural products (ISDB-LOTUS) matching along with taxonomical and chemical consistency reweighting,^33−35^ and (4) chemical structure and chemical class annotation using SIRIUS/CSI:FingerID^36−39^ and CANOPUS, respectively^40^ (Figure 2B, steps 3 to 6). Comparing structural annotations results from two different approaches (here ISDB-LOTUS and SIRIUS) allows the user to strengthen confidence in spectral annotation(s)—if both outputs are structurally similar—or penalize annotation(s)—if both outputs are highly dissimilar. At each processing step, the parameters used are saved to ensure reproducibility.

The previously generated set of data and information are then semantically enriched to construct a KG structure; for this, the next steps require the representation of data as subject–predicate–object (RDF triple) and reconciliation with external identifiers (Wikidata chemical structures, for example) when possible (Figure 2C). Data representation as triples implies, for example, linking a given LC-MS feature to its corresponding SIRIUS annotation by a predicate (see a visual representation of the triple). In Figure 2C, some representative examples illustrate how predicates link different subjects and objects to form a KG. To incorporate MS^2^ information into the KG, each LC-MS feature’s MS^2^ fragmentation spectrum is formatted as a document containing peaks and neutral losses using the spec2vec package.^41^ This incorporation of spectral data is innovative and of great interest as it enables the interlinking of all entities of the KG, such as spectra, but also molecular structure or extracts, through subspectral features (peaks, losses, or groups thereof). This particular alignment is fully independent of the chromatographic conditions and allows the realization of powerful queries regarding the spectral relatedness of features originating from different samples (see, for example, Table 1, query 6, and query 9). The spectral annotations (non stereochemically-defined chemical structures identified by their 2D InChIKeys) are then linked, if present, with their stereochemically defined counterparts in WD (identified by their complete InChIKeys). This reconciliation allows the user to run federated SPARQL queries over ENPKG and WD to retrieve, for example, among a given sample’s structural annotations, the ones already reported in the same taxon (see Table 1, query 4). Additionally, all chemical structures are classified using the NPClassifier chemical taxonomy (at the pathway, superclass, and class levels).^19,42^ The chemical structures can also be enriched with their reported bioactivity against a selected biological target in ChEMBL.

**Table 1:** Examples of SPARQL Queries on the ENPKG

query #	query description	returns	link
**1**	How many features (PI and NI modes) have the same SIRIUS/CSI:FingerID and ISDB annotation?	33,254 features	SPARQL
**2**	Which samples have features (PI mode) annotated as aspidosperma-type alkaloids by CANOPUS with a probability score above 0.5, ordered by the decreasing count of features as aspidosperma-type alkaloids?	32 samples. The sample with the highest count of features annotated as aspidosperma alkaloids (74) is from *Tabernaemontana coffeoides* (Apocynaceae) seeds extract.	SPARQL
**3**	Among the structural annotations from *Tabernaemontana coffeoides* (Apocynaceae) seeds extract, which ones contain an aspidospermidine substructure?	3 distinct stereochemically undefined structures (2D InChiKey) that contain an aspidospermidine substructure.	SPARQL
**4**	Among the SIRIUS structural annotations from *Tabernaemontana coffeoides* (Apocynaceae) seeds extract, which ones are reported in the *Tabernaemontana* genus in WD?	17 distinct stereochemically undefined structures annotated by SIRIUS in *Tabernaemontana coffeoides* (Apocynaceae) seeds extract and reported in at least one *Tabernaemontana* sp.	SPARQL
**5**	Which compounds annotated in the active extract of *Melochia umbellata* have activity against *T. cruzi* reported (in ChEMBL), and in which taxon they are reported (in Wikidata)?	14 distinct stereochemically undefined structures, reported in *Waltheria indica*, *Waltheria communis*, *Antidesma venenosum*, *Antidesma membranaceum*, or *Melochia chamaedrys*.	SPARQL
**6**	Which features have the most fragments and neutral losses in common with feature id 1 from *Waltheria indica* aerial part in PI mode (the [M + H]^+^ ion of waltherione G in this extract)?	91,758 features with at least 1 fragment or neutral loss in common with feature id 1 from *Waltheria indica* aerial part in PI mode. The feature with the most fragments and neutral losses in common (20) is feature id 1 from the anti-*T.cruzi* active extract of *Melochia umbellata*.	SPARQL
**7**	Filter the PI mode features of *Melochia umbellata* annotated as [M + H]^+^ by SIRIUS to keep the ones for which a feature in NI mode is detected with the same retention time (±3 s) and a mass corresponding to the [M – H]^−^ adduct (±5 ppm).	62 features from *Melochia umbellata* in PI mode annotated as [M + H]^+^ by SIRIUS with their corresponding potential [M – H]^−^.	SPARQL
**8**	For features from *Melochia umbellata* in PI mode with SIRIUS annotations, get the ones for which a feature in NI mode with the same retention time (±3 s) has the same SIRIUS annotation (2D IK).	22 features in PI mode for which a feature in NI mode with the same retention time has the same annotation.	SPARQL
**9**	Return extracts from the Korean medicinal plants data set containing the 5 features which are the most spectrally related (most fragments and neutral losses in common) to a feature from the 1,600 plants data set annotated as scopolamine. Return, via a Wikidata federated query, the species and upper taxonomy of the selected extracts.	5 features in PI mode. The two first features belong to extracts of plants of the Solanaceae family (known producers of tropanic alkaloids).	SPARQL

Finally, all the above-generated data are formatted in RDF turtle format (.ttl files) using the RDFlib python package,^43^ shared in Zenodo repositories at the ENPKG Zenodo community, and gathered as a single KG managed using GraphDB. GraphDB can then be used to visualize and mine the generated data through various SPARQL queries, as exemplified in the next section. All the scripts used for this data treatment are available for examination and reuse on the ENPKG GitHub organization, with an overview of the workflow and the links to the different repositories available at https://github.com/enpkg/enpkg_workflow.

### 2.3 Application of the ENPKG Framework to the Exploration of Large Data sets of Chemo-Diverse Plant Extracts

To benchmark its applicability, we applied the developed workflow to explore a data set of 1,600 plant extract samples previously published and described in 2022.^14^ In parallel to their LC-MS^2^ profiling in positive (PI) and negative (NI) ionization modes, these samples were screened against three human health-relevant trypanosomatids: *L. donovani*, *T. cruzi*, and *T. brucei rhodesiense*. To illustrate the capacities of the ENPKG approach for incremental sample addition, we also integrated data from three *Waltheria indica* (Q7966688) samples acquired in the context of a previous project at our lab, including samples obtained using different extraction protocols and profiled in 2014 in PI mode on a different analytical platform.^14^ We also integrated publicly available data from 337 methanolic extracts of Korean Pharmacopoeia plants profiled in PI mode on a Q-ToF spectrometer (MSV00008616).^24^ After processing through the ENPKG workflow, the data were integrated into a single KG, available at https://enpkg.commons-lab.org/graphdb/, which includes, at the time of publication, over 161 million statements.

#### 2.3.1 Description of the Obtained Knowledge-Graph and Examples of Queries

Interaction with the generated ENPKG is achieved through the SPARQL query module of the GraphDB instance available at https://enpkg.commons-lab.org/graphdb/sparql. It is first possible to get a broad overview of the dimensions of the generated data through simple queries. Hereafter, we describe the obtained results and the respective SPARQL queries as hyperlinks with the results at the time of publication. For example, the MZmine data processing yielded 788,623 and 364,967 features in positive ionization (PI) and negative ionization (NI) modes, respectively. Among these 1,153,590 features, 343,787 have an ISDB annotation, 846,850 have a SIRIUS/CSI:FingerID annotation, and 899,718 have a CANOPUS chemical class annotation. The structural annotations (ISDB and SIRIUS) correspond to 106,647 distinct planar structures (i.e., 2D InChiKeys), corresponding to the structures of 138,146 different stereochemically defined compounds on WD. It is also possible to query extracts’ bioactivity data, for example to retrieve the 8 non-cytotoxic extracts presenting an activity against *T. cruzi*. While these queries are mostly descriptive, SPARQL allows for more elaborated queries and is a powerful tool for gaining insights about the data set, as exemplified below.

It is, for instance, possible to combine the ISDB and SIRIUS/CSI:FingerID structural annotations to retrieve the 33,254 features annotated with the same structure by both approaches (Table 1, query 1). Using CANOPUS annotations, it is possible to retrieve the samples with the most annotations belonging to a given chemical class, such as aspidosperma-type alkaloids (Table 1, query 2). This query showed that the extract presenting the highest number of features (74) annotated with this chemical class was the one from the seeds of *Tabernaemontana coffeoides* (Q15376858) (Apocynaceae (Q173756)). Based on this information, using a federated query with the SACHEM IDSM end point,^51^ it is possible to further refine the search to retrieve 3 structural annotations from this extract that contain aspidospermidine (Q15410259) as a substructure (Table 1, query 3). Taking advantage of the links to WD, it is also possible to enhance the results of SIRIUS/CSI:FingerID structural annotations with their corresponding biological sources and retrieve the ones (17 planar structures at the time of writing) reported in the *Tabernaemontana* genus (Q310915) (Table 1, query 4). It is to be noted that this number, and all other results depending on Wikidata federated queries, might evolve if new organism–structure pairs are added (or removed) to (from) Wikidata.

Thanks to the integrated ChEMBL data, retrieving annotated compounds with reported activity against a specific target is possible. We applied this approach to retrieve the ChEMBL-reported anti-*T. cruzi* activities of compounds annotated in the *Melochia umbellata* [(Q6813281) (Malvaceae, Q156551)] extract we had evaluated as active against *T. cruzi*. For the 10 distinct returned compounds, we also queried the taxa in which they are reported from WD, returning 14 structure-organism pairs at the time of writing (Table 1, query 5). Interestingly, among these 10 distinct compounds annotated in the active *M. umbellata* extract, all are quinoline alkaloids reported in *Waltheria indica* (Malvaceae). The overall chemical similarity between these two taxa is confirmed at the experimental spectral level using the integration of features’ peaks and neutral losses into ENPKG. For this we designed a SPARQL request that can be used to retrieve the features with the highest number of peaks and neutral losses in common with the MS^2^ spectrum of the [M + H]^+^ ion of waltherione G (Q110090875) detected in the *Waltheria indica* (Q7966688) aerial parts extract profiled in 2014 using another LC method and another Orbitrap spectrometer (Table 1, query 6).^14^ This query reveals that the feature sharing the highest number of peaks and losses among those from the set of 1,940 PI analyses is feature id 1 in the PI mode analysis of the active extract of *Melochia umbellata* (with a confirmed cosine similarity of 0.98). This feature shares 20 peaks and neutral losses with the waltherione G [M + H]^+^ ion, and the fasst search against the GNPS libraries further points toward a strong spectral match with waltherione G. Together, the previous SPARQL queries allowed us to putatively identify the compound(s) responsible for the observed antitrypanosomatid activity of *M. umbellata*. Such possibilities are exciting for exploring spectral data without necessarily relying on the preliminary establishment of structural annotations, RT-based alignment, or MN. These observations confirm previously published findings regarding the chemical contents and bioactivity potential of this active extract and the potentially active compounds it contains.^14,44,45^ While a labor-intensive data inspection is classically required to link an extract’s activity to the responsible compounds, this can now be expedited using a single SPARQL query, saving precious time and resources. This example illustrates the benefits of a single interface integrating spectral, chemical, taxonomic, and bioactivity data.

Finally, the KG structure allows for the organization and queries of heterogeneous data sets. As an illustration, we searched the Korean medicinal plants data set for analogs of a feature annotated as scopolamine in the 1,600 plant extracts data set. To do so, we queried the top 5 features from the Korean plants data set with the most common fragments and neutral losses with the feature of interest, along with the botanical family, genus, and species of the corresponding samples, retrieved through a federated Wikidata search (Table 1, query 9). The top two features are from *Datura metel* (Q715019) and *Scopolia japonica* (Q869524), both from the Solanaceae family (Q134172). The feature in *Datura metel* was putatively identified as scopolamine; see the corresponding fasst search in the GNPS libraries. The feature highlighted in *Scopolia japonica* apparently corresponds to a dihydrogenated version of scopolamine (see corresponding fasst search in the GNPS libraries). The integration of this metabolomics data set and the associated SPARQL query illustrate the power of the proposed approach, which allows for the search of common chemistries across metabolomics data sets acquired by different researchers, at different times using different platforms (Orbitrap vs QToF) under different chromatographic conditions (8 min versus 20 min runs) and extracted using different solvents (EtOAc vs MeOH). Some additional examples of applications are given in Table 1.

#### 2.3.2 Specific Applications in a Drug Discovery Context

The transformation of large metabolomics data sets in a queryable KG structure enhanced by the connection with public electronic resources offers exciting possibilities in the frame of drug discovery research programs. Indeed, the whole process can be viewed as a “virtual fractionation” of the profiled extracts, which, when applied to large extract collections, has been proven to help in the removal of common metabolic background across extracts and to efficiently highlight chemical scaffolds responsible for the observed bioactivities at the extract level, without passing by the cumbersome physical fractionation of the individual extracts.^12^ Here, the approach is pushed further and leverages the precise results of the high-throughput reductionist mass-spectrometry fragmentation process — information is obtained down to the submolecular level in the forms of singular peaks and losses — and enhances them through a contextualization process inherent to the KG structure. The reconciliation of chemical structure identifiers allows to connect metabolite annotation results to public resources documenting the bioactivity of molecular structures (e.g., ChEMBL). This offers precious information for the identification of bioactive molecular structures before their physical isolation, a process we define here as *biodereplication* and illustrate through the following examples. We would like to underline that if we have here incorporated ChEMBL data sets relevant to our current drug discovery objectives (i.e., antitrypanosomatids), there are no restrictions to the incorporation of other bioactivity data sets comprised of chemical structure and their evaluated bioactivity on any given target — provided that these are shared publicly. This opens exciting possibilities as it becomes possible to detect spectrally related analogs of compounds previously bioassayed by others, hence opening the possibility to fish potential bioactive compounds from complex extracts library *without* even realizing the initial screening campaigns nor the bioguided fractionation process.

##### 2.3.2.1 Identification of Potent Anti-*Trypanosoma cruzi* Agents

**Figure 3:**
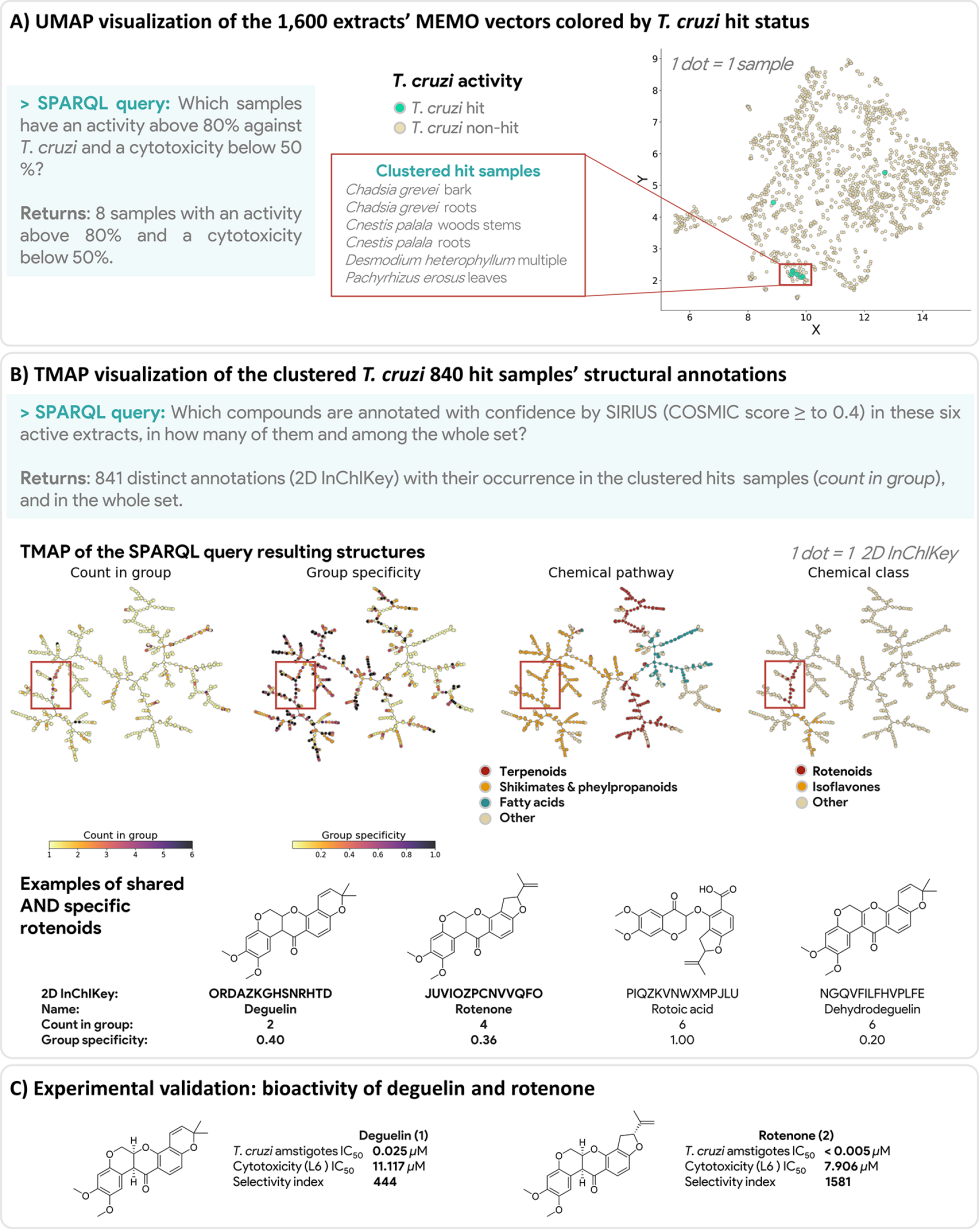
Identification of rotenoids derivatives as responsible for the anti-*T. cruzi* of 6 active extracts. (A) The anti-*T. cruzi* activity of extracts mapped on the MEMO UMAP showed a cluster of six active extracts. Link to query. (B) An analysis of structural annotations within these six active extracts showed that a particular class of compounds (rotenoids) is both shared among them (high *count in group*) and specific to them (high *group specificity*), suggesting that these compounds may be responsible for the extracts’ activity. Link to query. (C) This hypothesis was confirmed experimentally by evaluating the anti-*T. cruzi* activity of two of the annotated rotenoids, deguelin and rotenone.

As mentioned previously, the unaligned LC-MS^2^-generated data are also suited for MEMO analysis to compare samples’ MEMO vectors.^14^ The following example shows that MEMO-based visualizations and the ENPKG architecture are complementary ways to extract knowledge from such a data set. By using UMAP to visualize the MEMO vector similarity of the 1,600 extracts screened against *T. cruzi*, we can observe, among the 8 active extracts against *T. cruzi*, a cluster of 6 samples (Figure 3A).^46^ These 6 samples originate from 4 different species: *Desmodium heterophyllum* (Q10770714, Fabaceae), *Chadsia grevei* (Q15528494, Fabaceae), *Pachyrhizus erosus* (Q517283, Fabaceae), and *Cnestis palala* (Q15231964, Connaraceae). Following this observation, one can hypothesize that this clustering is due to structurally similar compounds in these extracts that could explain the similar bioactivity profiles. Using the following query, it is possible to retrieve 841 compounds annotated in at least one of these 6 selected extracts and quantify their occurrence both in the cluster of active extracts and among the whole set (Figure 3B). By visualizing these chemical annotations and their relationships in the form of a TMAP, it is possible to spot a cluster of compounds, mainly rotenoids derivatives, that are both shared among active extracts (high “count in-group”) and specific to this group (high “group specificity”).^47^ This shows here the interest of integrating data from inactive samples in the analysis. By looking only at common annotations in active extracts, rotenoids and fatty acids derivatives are highlighted (Figure 3B, first TMAP from the left). However, by looking at the specificity of these compounds, we can observe that only the rotenoids are specific to the cluster of active extracts, while fatty acids are spread among a large number of samples and thus less likely to be responsible for the activity (Figure 3B, second TMAP from the left). It is also possible to spot rotenoids as specific of these active extracts using the CANOPUS chemical class annotations (Figures S2 and S3). Indeed, these six active extracts present a count of rotenoid annotations superior to 40, while no other sample presents a count superior to 12 (query). The specific presence of these compounds common to most of these six active extracts suggests they are responsible for this activity. In addition, a search of ChEMBL reported anti-*T. cruzi*, *T. brucei*, or *L. donovani* activity among annotated compounds in these extracts returns 8 distinct compounds but no rotenoids, confirming the potentially novel activity of this class of compounds against *T. cruzi* (query).

To confirm this hypothesis, we evaluated two commercially available rotenoids, which were annotated in the data set: deguelin (**1**) (Q5251862) and rotenone (**2**) (Q412388), in the same antiparasitic assay used for the extracts screening (Figure 3 C). These two compounds were found to be extremely potent, presenting IC_50_ in the nanomolar range (0.025 and <0.005 μM for deguelin and rotenone, respectively), coupled with low cytotoxicity to the host cell (11.117 and 7.906 μM respectively). This is the first report of the activity of deguelin and rotenone, and, more broadly speaking, of rotenoids as a chemical class, against intracellular *T. cruzi* amastigotes despite rotenone being used as a pharmacological tool in several *Trypanosoma* metabolism studies.^48,49^ This discovery illustrates how the combination of MEMO-based visualizations and the ENPKG workflow enables hypotheses to be efficiently formulated and tested on a natural extract data set, which can lead to the rapid identification of bioactive molecules.

##### 2.3.2.2 Streamlined Identification and Isolation of Active Compounds Analogues

Modern MS^2^ annotation techniques keep improving and allow accurate annotation of an increasing number of features.^10,33,34,36^ With these improvements, dozens to hundreds of potential structures are putatively annotated in each extract, allowing switching from an extract library to a virtual chemical compound library perspective. The annotations can be used to look for structures or substructures of interest in one or multiple extracts, which can then be fractionated to obtain the pure compound(s) of interest and confirm or infirm its structural annotation.^50^ Such an approach is particularly interesting in drug discovery efforts to target potential analogs of compounds with promising activity. By standardizing the chemical information and integrating Wikidata and ChEMBL compounds’ bioactivity information, the ENPKG workflow allows fast retrieval of both confidently annotated compounds and compounds with reported activity. In addition, it can also take advantage of the federated query mechanism through the Sachem chemical structural similarity cartridge, on available Wikidata structures.^51^ As an illustration, we report the isolation and characterization of a triterpenoid quinone methide, 11β-hydroxypristimerin (**3**), and its *in vitro* activity against *L. donovani*.

**Figure 4:**
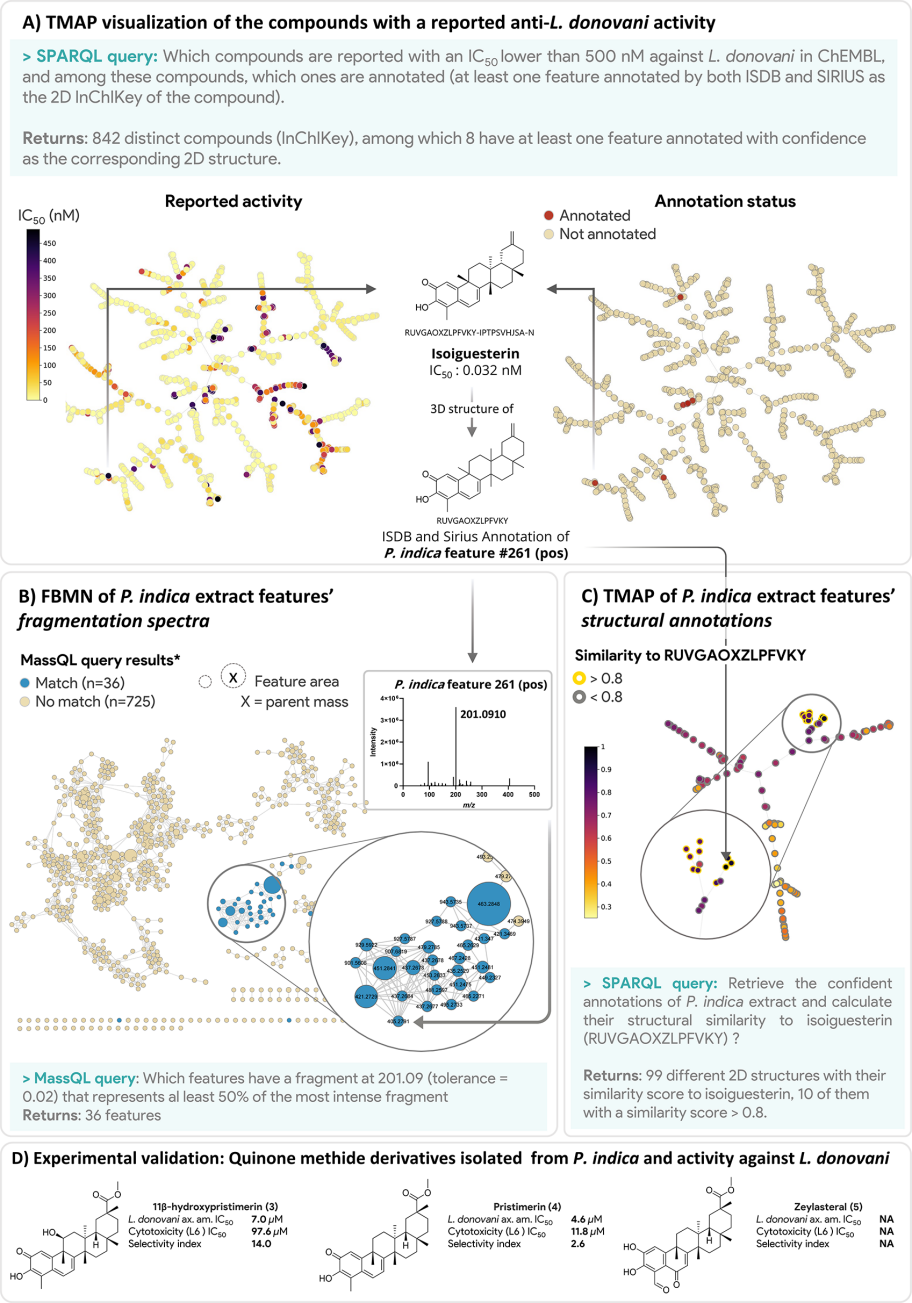
Identification of active triterpenoid quinone methide derivatives in *P. indica* extract using ChEMBL, SIRIUS, ISDB, MassQL, and MN data embedded in the ENPKG. (A) Using SPARQL, it is possible to retrieve compounds with high activity against *L. donovani* reported in ChEMBL and annotated in the sample set. Link to query. (B) Among compounds reported as active and annotated and with confidence in the set (8 compounds), isoiguesterin is annotated in *Pristimera indica*. The feature annotated as isoiguesterin (feature #261 in PI of *P. indica*) presents a fragment at *m*/*z* 201.09 characteristic of the quinone moiety. Using MassQL, it is possible to highlight 36 features in the PI mode FBMN of *P. indica* that present this fragment and are potential quinone methide analogs. (C) Using SPARQL and the annotations data, it is possible to retrieve 10 planar structures among the 99 *P. indica* confident annotations (SIRIUS annotations with a COSMIC score > 0.5 and a ZODIAC score > 0.8, and ISDB annotations with a taxonomical score ≥ 6), with a Tanimoto similarity with isoiguesterin superior or equal to 0.8, confirming the results obtained on the spectral data. Link to query. (D) Targeted isolation on *P. indica* extracts led to the obtention of three quinone methide derivatives: pristimerin, zeylasteral, and 11β-hydroxypristimerin. The bioactivity of 11β-hydroxypristimerin (3) and pristimerin (4) was evaluated in vitro, confirming their activity against *L. donovani* axenic amastigotes. NA: not applicable (isolated in insufficient amount).

Triterpenoid quinone methides derivatives have been reported as promising hits against *L. donovani* promastigotes, in particular, 20-*epi*-isoiguesterinol (RRKSDDREVOXSJD-TXARQUJHSA-N (Q27134610), IC_50_ = 0.027 μg/mL or 0.064 μM) and isoiguesterin (RUVGAOXZLPFVKY-IPTPSVHJSA-N (Q27134609), IC_50_ = 0.032 μg/mL or 0.079 μM).^52^ Isoiguesterin (Q27134609), is annotated with confidence by SIRIUS/CSI:FingerID and ISDB in *Pristimera indica* (Q11075650, Celastraceae) roots extract (feature #241 in PI mode) (query, Figure 4A). When studying the MS^2^ spectrum of this feature (see GNPS dashboard visualization), one can observe a fragment at *m*/*z* 201.09 characteristic of the quinone methide moiety of these triterpenoids (Q7844276).^53^ Using the recently developed MassQL language, it is possible to retrieve all features corresponding to a fragmentation spectrum in which this fragment is observed.^54^ This search revealed that among the 761 features of this extract (PI mode), 36 present this characteristic fragment with an intensity representing at least 50% of the most intense peak of the spectrum (MassQL query job available here). A visualization of the results of this query on the generated MN for the *P. indica* extract showed that these analogs are part of a cluster of potential structural analogs (Figure 4B).^32^ At the structural annotation level, it is possible to use the Sachem cartridge to retrieve analogs of a compound of interest among other WD compounds.^51^ Such an analysis revealed that, among 99 different confident structural annotations, 10 structures have a Tanimoto similarity superior or equal to 0.8 against isoiguesterin (query, Figure 4C). This example shows how the ENPKG structure can be exploited to take advantage of raw or semi-interpreted data (spectral data in this example) or processed data (annotations) to investigate a research question rapidly.

Based on the hypothesis mentioned above, the targeted phytochemical investigation of the *P. indica* roots extract led to the isolation of three triterpenoid quinone methide derivatives: 11β-hydroxypristimerin (**3**), pristimerin (**4**) (Q27108073), and zeylasteral (**5**) (Q105027438) (Figure 4D).^55−57^ For the structural elucidation of isolated compounds, see Materials and Methods. The bioactivity of pristimerin and 11β-hydroxypristimerin was evaluated *in vitro* (zeylastral was not isolated in sufficient amounts for biological evaluation), and their activity was confirmed with IC_50_ of 7.0 and 4.6 μM against *L. donovani* axenic amastigotes, for **3** and **4** respectively. Interestingly, 11β-hydroxypristimerin presented a higher selectivity index (14.0) on *L. donovani* axenic amastigotes than pristimerin (2.6), a compound reported as highly active against *L. donovani*.^58^

### 2.4 Overview, Current Limitations, and Future Improvements

Overall, these different application examples showcase some of the exciting opportunities offered by the ENPKG framework. The possibility of querying linked spectral, chemical, taxonomical, and bioactivity data of thousands of samples using a single technology streamlines the exploration of such multidimensional data sets. The workflow’s key features include the *sample-centric approach* and the *efficient connection to existing public resources*. On the one hand, the sample-centric approach ensures that the data generated over time can be added to existing data set(s) quickly and efficiently, as demonstrated through the integration of samples profiled several years apart on different analytical platforms. This possibility of incremental addition of samples opens the door to efficient large-scale (re)analysis, known as repository-scale analysis.^59^ In addition, the deposition of all LC-HRMS and spectral data on the GNPS MassIVE repository allows their straightforward visualization (LC-MS^2^ chromatograms and fragmentation spectra, using the GNPS dashboard) and the rapid launch of in-depth spectral analysis on selected samples or spectra (e.g., using MASST, MassQL, or other GNPS workflows).^10,28,54,60^ On the other hand, connection to existing databases allows a more direct interpretation of metabolomics data by matching the ‘*known*’ with the ‘*unknown*’. Using federated queries, it is possible to connect experimental datasets to publicly available and semantically enriched information. This step depends on the public availability of curated DBs, highlighting the central role of these infrastructures in advancing science.

For approaches like the ones we have presented to be more widely adopted, several aspects must be improved. First, it is essential to establish richer metadata collection mechanisms at the public mass spectrometry repository level. The ReDU framework represents a fundamental advancement in this sense.^29^ Future developments could take advantage of the progress made by initiatives such as the CEDAR workbench (https://metadatacenter.org/) and open-source toolkits such as Frictionless (https://frictionlessdata.io/) as powerful ways to standardize and facilitate metadata collection protocols. Other examples of community initiatives aiming to standardize MS instrumental metadata^61^ or sample metadata (https://github.com/ERGA-consortium/ERGA-sample-manifest) have been described. The steps involved in transitioning from raw mass spectrometry data to Linked Open Data formats for building a KG should be further standardized. This standardization will require strengthening the current data processing workflow through sounder data validation processes and the development of simplified user interfaces to facilitate data deposition. We would like to underline that in the current work, we have employed a series of tools and strategies commonly used in our laboratories for the peak-picking (i.e., MzMine) and metabolite annotation stage (i.e., Sirius, taxonomically informed metabolite annotation using the ISDB-LOTUS). These tools are, however, not the only ones available, and the ENPKG workflow should be adapted in the future to handle better the output of other peak-picking (*e.g.,* XCMS or asari), metabolite annotation strategies (*e.g.,* MSDial), or chemical taxonomies (e.g., ChemOnt from ClassyFire).^62−65^ An additional area of improvement concerns the query mechanisms. The SPARQL can extract valuable insights from a graph but may challenge inexperienced users. It will thus be necessary to work on interfaces designed to facilitate such interactions. In this respect, we currently explore natural language processing approaches to generate SPARQL from plain-text prompts.^66^ These tailored human-data interfaces offer promising perspectives for a wider adoption of the proposed ENPKG approach.

## 3 Conclusion

We developed a novel sample-centric and knowledge-driven computational metabolomics pipeline to explore large NPs extract data sets. This approach ensures an efficient integration of new samples over time while limiting the loss of valuable experimental data in hermetic project-related silos. The employed semantic web technologies facilitate the merging of experimental data with knowledge available from existing public scientific resources. We showcased the utility of this pipeline in a drug discovery context by exploring a 1,600 plant extract collection that was screened against trypanosomatids. Here, we could rapidly annotate *known* anti-*T. cruzi* compounds in an active extract, annotate *unknown* anti-*T. cruzi* compounds common to active extracts, and identify an active analog of reported anti-*L. donovani* compounds. These different applications demonstrate the flexibility of the developed framework and the possibility it offers to explore NPs metabolomics data in drug discovery context and beyond. Great advances still need to be made to democratize semantic web technologies in NPs research, yet we anticipate that computational workflows and knowledge management solutions such as ENPKG have the potential to fundamentally reshape current approaches employed to explore chemodiversity and improve the reusability of knowledge obtained during NPs metabolomics projects.

## 4 Materials and Methods

### 4.1 LC-HRMS^2^ Analysis

#### 4.1.1 1,600 Plant Extract Data Set

For PI mode, see section 2.2.2 LC-MS^2^ Analysis” in ref (14). For NI mode, MS parameters were set as follows. The optimized HESI-II parameters were as follows: source voltage, 2.5 kV (neg); sheath gas flow rate (N2), 55 units; auxiliary gas flow rate, 15 units; spare gas flow rate, 3.0; capillary temperature, 450.00 °C, S-Lens RF Level, 45. The mass analyzer was calibrated using a mixture of caffeine, methionine–arginine–phenylalanine–alanine–acetate (MRFA), sodium dodecyl sulfate, sodium taurocholate, and Ultramark 1621 in an acetonitrile/methanol/water solution containing 1% formic acid by direct injection. The data-dependent MS^2^ events were performed on the three most intense ions detected in full scan MS (Top3 experiment). The MS^2^ isolation window width was 1 Da, and the stepped normalized collision energy (NCE) was set to 15, 30, and 45 units. In data-dependent MS^2^ experiments, full scans were acquired at a resolution of 35,000 fwhm (at *m*/*z* 200) and MS^2^ scans at 17,500 fwhm, both with an automatically determined maximum injection time. After being acquired in an MS^2^ scan, parent ions were placed in a dynamic exclusion list for 2.0 s.

#### 4.1.2 Waltheria Indica Samples

For *Waltheria indica* samples (PI mode only), see section 2.3.2 LC-MS/MS Analysis in ref (14).

#### 4.1.3 Korean Pharmacopeia Plants Extracts

For details regarding the analysis of these extracts, see Kang KB et al.^24^

### 4.2 LC-HRMS^2^ Data-Processing

#### 4.2.1 1,600 Plant Extracts Data Set

For MZmine 2 parameters in PI mode, see “Plant Extract Dataset” in ref (14). For negative mode, the MS data were converted from RAW (Thermo) standard data format to mzXML format using the MSConvert software (v3.0.10385), part of the ProteoWizard package.^67^ The converted files were treated using the MZMine software suite v.2.53.^26^ The parameters were adjusted as follows: the centroid mass detector was used for mass detection with the noise level set to 1.0E4 for MS level set to 1, and to 0 for MS level set to 2. The ADAP chromatogram builder was used and set to a minimum group size of scans of 5, minimum group intensity threshold of 1.0E4, minimum highest intensity of 5.0E5, and *m*/*z* tolerance of 12 ppm.^68^ For chromatogram deconvolution, the algorithm used was the wavelets (ADAP). The intensity window S/N was used as an S/N estimator with a signal-to-noise ratio set at 10, a minimum feature height at 5.0E5, a coefficient area threshold at 50, a peak duration range from 0.02 to 0.5 min, and the RT wavelet range from 0.01 to 0.03 min. Isotopes were detected using the isotope peaks grouper with an *m*/*z* tolerance of 12 ppm, an RT tolerance of 0.01 min (absolute), the maximum charge set at 2, and the representative isotope used was the most intense. Each feature list was filtered using the feature filtering module to keep only features with an associated MS^2^ scan and an RT between 0.5 and 8.0 min. Note that these details are embedded inside the ENPKG; see enpkg:has_lcms_feature_list_a6a5420d414df1000ab74a2b82275839 (PI) and enpkg:has_lcms_feature_list_d5f38c47bc9e90a297d4c26ee02d05b5 (NI)

#### 4.2.2 Waltheria Indica Samples

For *Waltheria indica* samples (PI mode only), see section 2.3.3 Data-Processing in ref (14). See also enpkg:has_lcms_feature_list_a6a5420d414df1000ab74a2b82275839 (PI).

#### 4.2.3 Korean Pharmacopeia Plants Extracts

See enpkg:has_lcms_feature_list_a137fd4a263d3587d35f61a526932c09 (PI).

For all data sets in PI and NI (when available) modes, individual feature lists’ quantification tables and their associated MS^2^ spectra were exported for each sample individually using the “Export to GNPS” module. Features MS^1^ isotopic pattern and MS^2^ spectra were also exported using the “Export for SIRIUS” module for subsequent SIRIUS analysis. Exported files, together with the metadata (producing organism, bioactivity, etc.), were organized in a sample-wise folder architecture using scripts available at https://github.com/enpkg/enpkg_full/tree/main/01_enpkg_data_organization. Raw LC-MS^2^ data (.mzML) and individual deconvoluted. mgf files containing the features’ MS^2^ spectra have been uploaded to MassIVE for data-sharing and to allow for GNPS-related analyses (MASSIVE ID: MSV000087728 for the 1,600 plant extracts data set, MSV000088521 for *Waltheria indica* samples, and MSV000093464 for the Korean medicinal plants data set).

### 4.3 Data Treatment at the Sample Level

These data-treatment steps are performed at the level of each sample directory previously generated. The scripts used are part of the ENPKG workflow and are publicly available on GitHub. An overview of the different steps and the links to the corresponding directories are available at https://github.com/enpkg/enpkg_workflow and at https://github.com/enpkg/enpkg_full.

#### 4.3.1 Taxonomic Resolution of the Biosource

The name of the producing organism (binomial nomenclature) of each sample was resolved using the Open Tree of Life (OTT) taxonomy (v3.5) to retrieve the OTT ID and the Wikidata ID.^30,31^ The scripts used for this taxonomic resolution are available in https://github.com/enpkg/enpkg_full/tree/main/02_enpkg_taxo_enhancer.

The version and parameters used can be directly fetched from the graph as sub Properties of an enpkg:has_wd_id. For example under enpkg:has_wd_id_c18527bea8b2606a55457d607b24df69.

#### 4.3.2 Molecular Networking

Molecular networking was performed in Python using the matchms (v0.20.0) package^32^ for both PI and NI modes. The *m*/*z* tolerance for fragment matching was set to 0.01, and the modified cosine score cutoff for edge creation was set to 0.7. The *max_links* parameter was set to 10 with a *top_n* parameter set to 15. The scripts used for Molecular Networking are available at https://github.com/enpkg/enpkg_full/tree/main/03_enpkg_mn_isdb_isdb_taxo.

The version and parameters used can be directly fetched from the graph as sub Properties of an enpkg:has_mn_params. For example under enpkg:mn_params_f4fec9f496001612d60a75b5e1a43991.

#### 4.3.3 *In Silico* Spectral Annotation and Chemotaxonomical Reweighting

Spectral matching was performed in Python using the matchms (v0.20.0) package^32^ against an *in silico* database (ISDB-LOTUS^35^) of fragmented natural products structures^33,69^ for both PI and NI modes. The *m*/*z* tolerance for parent mass and fragment matching was set to 0.01. The minimal cosine score for matching was set to 0.2 with 6 minimal matching fragments. Also, potential adducts of compounds reported in the species of the processed sample were considered for annotation based on the parent mass only (MS^1^ annotation, ppm tolerance set to 10). Matching candidates were reranked according to the taxonomic distance between the producing organism of the candidate structure and the sample’s organism.^34^ Finally, candidate structures were reweighted according to the MN cluster chemical consistency following the Network Annotation Propagation principle.^70^ The best-ranked candidate for each feature was finally selected. The scripts used for spectral matching and taxonomical/chemical reweighting are available at https://github.com/enpkg/enpkg_full/tree/main/03_enpkg_mn_isdb_isdb_taxo.

A graphical description of this annotation workflow is presented in Figure S21. The version and parameters used can be directly fetched from the graph as sub Properties of an enpkg:has_isdb_annotation, for example, under enpkg:has_isdb_annotation_95a24ed68ee3b548d93d96b99ba630c4.

#### 4.3.4 SIRIUS, CSI:FingerID and CANOPUS Annotation

Exported features’ MS^2^ spectra were submitted to SIRIUS/CSI:FingerID and CANOPUS annotation. For PI mode, SIRIUS v4.9.12, SIRIUS lib v4.6.1, and CSI lib v1.6.0 (v5.5.7, v4.12.3, and v2.6.3 respectively, for *Waltheria indica* samples) were used with following parameters: a ppm tolerance set to 10, maximum *m*/*z* of 800, 10 candidates considered with the following adducts: [M + H]^+^, [M + NH_4_]^+^, [M + K]^+^, [M + Na]^+^. For structural matching, the biological databases were considered. For NI mode, SIRIUS v5.5.4, SIRIUS lib v4.12.3, and CSI lib v2.6.3 were used with the following parameters: a ppm tolerance set to 10, maximum *m*/*z* of 800, 10 candidates considered with the following adducts: [M – H]^−^, [M + K-2H]^−^, [M + Na-2H]^−^. For structural matching, the biological databases were considered. For both ionization modes, CANOPUS was run (no parameter needed). The script to automate SIRIUS and CANOPUS annotation on the ENPKG folder architecture is available at https://github.com/enpkg/enpkg_full/tree/main/04_enpkg_sirius_canopus.

The version and parameters used can be directly fetched from the graph as sub Properties of an enpkg:has_sirius_annotation. For example under enpkg:has_sirius_annotation_00c339c3b183cb7fbf466b4d334dbef2

#### 4.3.5 Chemical Structures Metadata Fetching and Wikidata Integration

The NPClassifier taxonomy of all 2D structures were retrieved by submitting their anisomeric SMILES to the NPClassifier API.^42^ Finally, the 2D InChiKeys were mapped against all WD compounds to retrieve the corresponding WD ID of the compounds sharing the same 2D IK. The script used for this processing is available at https://github.com/enpkg/enpkg_full/blob/d625e1a7a3365dd2fd70c66b00f8a59a80fd97a3/05_enpkg_meta_analysis/src/chemo_info_fetcher.py.

#### 4.3.6 Graph Building

The previously generated data were formatted as RDF using scripts available at https://github.com/enpkg/enpkg_full/tree/main/06_enpkg_graph_builder. The version used for the data presented here is available at https://github.com/enpkg/enpkg_full/tree/d625e1a7a3365dd2fd70c66b00f8a59a80fd97a3/06_enpkg_graph_builder.

The data used to build the herein presented KG are publicly available (see the Data availability statement). The generated knowledge graph is available at https://enpkg.commons-lab.org/graphdb/.

### 4.4 Sample-Set Data Treatment

#### 4.4.1 MEMO Analysis

To compare the total spectral content of the different samples (1,600 plant extracts data set only), we used our recently developed MEMO analysis (v0.1.4).^14^ Spectra were processed as follows in PI and NI modes. Only peaks with a relative intensity between 0.01 and 1 were kept for each spectrum, and spectra with less than 10 peaks were discarded. Losses between 10 and 200 *m*/*z* to the precursor were calculated, and the resulting spectra were translated into two decimals documents using spec2vec (v0.6.0).^41^ Finally, peaks and losses occurring in blank samples were removed. The script used is available at https://github.com/enpkg/enpkg_full/blob/d625e1a7a3365dd2fd70c66b00f8a59a80fd97a3/05_enpkg_meta_analysis/src/memo_unaligned_repo.py.

#### 4.4.2 ChEMBL Compounds Fetching

To allow for faster identification of compounds with an already reported activity against a given target, we implemented an automatic fetching and formatting of ChEMBL compounds with an activity reported against a given target.^71^ Using the Python ChEMBL web resource client (v0.10.8),^72^ all compounds with activity against a given target are retrieved with their associated metadata, such as ChEMBL ID, IChIKey, activity value, unit, type, reference, etc. The fetched data were standardized using RDKit (v2022.03.3),^73^ and the NP-likeness score of each compound was calculated to allow for filtering NP-unlike compounds.^74^ Compounds associated with activity against *Trypanosoma brucei rhodesiense* (CHEMBL612348), *T. cruzi* (CHEMBL368), or *Leishmania donovani* (CHEMBL367) with an NP-likeness score superior to −1 were retrieved (ChEMBL DB v30) and saved for integration in the knowledge graph. The script used for this step is available at https://github.com/enpkg/enpkg_full/blob/d625e1a7a3365dd2fd70c66b00f8a59a80fd97a3/05_enpkg_meta_analysis/src/download_chembl.py.

### 4.5 Plant Material, Isolation, and Structural Characterization of Pristimerin, Zeylastral, and 11-β-Hydroxypristimerin

The species *Prisitimera indica* (Q11075650) is part of the Pierre Fabre Laboratories (PFL) collection. The PFL collection is registered at the European Commission, receiving the accession number 03-FR-2020.^75,76^ The grounded dry roots plant material of *Prisitimera indica* (Willd.) A.C.Sm. was provided by the PFL (PFL identifier V113075).

The grounded dry roots (19.8 g) of *P. indica* were extracted successively with 200 mL of the following solvents (3 times each solvent and 24 h agitation): hexane, ethyl acetate, and methanol. Each extract was dried under a vacuum in a rotary evaporator at 35 °C to give: 61.5 mg of hexanic (RI-H), 103.7 mg of ethyl acetate (RI-A), and 728.3 mg of methanolic extract (RI-M).

Separations were performed in a semipreparative HPLC Shimadzu system equipped with LC-20A module pumps, an SPD-20A UV/vis, a 7725I Rheodyne valve, and an FRC-10A fraction collector (Shimadzu, Kyoto, Japan).

The RI-H (38.6 mg) was separated in an Xbridge C_18_ (250 × 19 mm i.d., 5 μm) column. The flow rate was set to 17 mL min^–1^, and a gradient elution was carried out with a binary solvent system of 0.1% aqueous formic acid [A] and 0.1% formic acid in acetonitrile [B]. A gradient (v/v) of [B] was used as follows [*t*(min), % B]: 0.00, 45; 30.00, 50; 100.00, 90; 110.00, 100; 115.00, 100; followed by re-equilibration steps (117.00, 45; 120.00, 45). The collection was done by volume (15 mL) and based on the 254 and 280 nm UV traces. A total of 33 fractions were collected. The fraction collected at *t*_R_ 70.0 min was purified in an Xbridge C18 (250 × 10 mm i.d., 5 μm) column, using an isocratic composition of acetonitrile + 0.1 formic acid: 0.1% aqueous formic acid 60:40 at a flow rate of 10 mL min^–1^ to afford 0.3 mg of zeylasteral (*R*_t_ 40.5 min). The fraction collected at t_R_ 88.0 min was purified in an Xbridge C18 (250 × 10 mm i.d., 5 μm) column, using an isocratic composition of acetonitrile +0.1 formic acid: 0.1% aqueous formic acid 70:30 at a flow rate of 10 mL min^–1^ to afford 1.3 mg of 11-*β*-hydroxypristimerin (*R*_t_ 40.5 min).

The RI-A (59.2 mg) was separated in an Xbridge C18 (250 × 19 mm i.d., 5 μm) column. The flow rate was set to 17 mL min^–1^, and a gradient elution was carried out with a binary solvent system of 0.1% aqueous formic acid [A] and 0.1% formic acid in acetonitrile [B]. A gradient (v/v) of [B] was used as follows [*t*(min), % B]: 0.00, 5; 5.00, 40; 52.00, 55; 77.00, 100; 82.00, 100; followed by re-equilibration steps (85.00, 5; 90.00, 5). The collection was done by volume (15 mL) and based on the 254 and 280 nm UV traces. This separation afforded 0.8 mg of pristimerin (*R*_t_ 83.0 min).

#### 4.5.1 Description of the Isolated Compounds

##### 4.5.1.1 11-β-Hydroxypristimerin (**3**)^57^

Amorphous dark orange powder, [α]_D_^20^ +21 (c 0.0009, MeOH). UV (MeH) λ_max_ 219 nm, 329 nm.

^1^H NMR (CDCl_3_, 600 MHz) δ 0.60 (3H, s, H_3_-27), 0.97 (1H, m, H_2_-22β), 1.09 (3H, s, H_3_-28), 1.19 (3H, s, H_3_-30), 1.29 (3H, s, H_3_-26), 1.39 (1H, td, *J* = 14.0, 13.2, 4.8 Hz, H_2_-21β), 1.50 (1H, dd, *J* = 14.0, 6.1 Hz, H_2_-16α), 1.58 (3H, m, H_2_-15, H-18), 1.63 (3H, s, H_3_-25), 1.73 (1H, dd, *J* = 16.2, 8.4 Hz, H_2_-19β), 1.86 (1H, td, *J* = 14.0, 6.1 Hz, H_2_-16β), 1.99 (1H, td, *J* = 14.0, 4.8 Hz, H_2_-22α), 1.99 (1H, t, *J* = 13.7, 12.2 Hz, H_2_-12β), 2.18 (3H, s, H_3_-23), 2.22 (1H, d, *J* = 13.2 Hz, H_2_-21α), 2.36 (1H, dd, *J* = 13.7, 6.4 Hz, H_2_-12α), 2.39 (1H, d, *J* = 16.2 Hz, H_2_-19α), 3.59 (3H, s, OMe), 4.56 (1H, dd, *J* = 12.2, 6.4 Hz, H-11), 6.26 (1H, d, *J* = 7.1 Hz, H-7), 6.87 (1H, d, *J* = 7.1 Hz, H-6), 7.31 (1H, s, H-1); ^13^C NMR (CDCl_3_, 151 MHz) δ 10.5 (CH_3_-23), 18.7 (CH_3_-27), 21.6 (CH_3_-26), 28.8 (CH_2_-15), 29.9 (CH_2_-21), 31.0 (C-17), 31.0 (CH_2_-19), 31.6 (CH_3_-28), 32.8 (CH_3_-30), 34.4 (CH_2_-22), 34.5 (CH_3_-25), 36.4 (CH_2_-16), 40.5 (C-20), 40.7 (C-13), 43.5 (CH_2_–12), 44.0 (CH-18), 45.0 (C-14), 48.2 (C-9), 65.5 (CH-11), 118.2 (C-4), 118.9 (CH-7), 121.8 (CH-1), 128.7 (C-5), 132.3 (CH-6), 146.1 (C-3), 161.7 (C-10), 167.3 (C-8), 178.3 (C-2), 178.9 (C-29). For NMR spectra, see Supplementary Figures S4–S9. HRESIMS *m*/*z* 481.2950 (calculated for C_30_H_41_O_5_, error 0.33 ppm).

##### 4.5.1.2 Pristimerin (**4**)^56^

Amorphous dark orange powder, [α]_D_^20^ −22 (c 0.0005, MeOH). UV (MeH) λ_max_ 214 nm, 422 nm.

^1^H NMR (CD_3_OD, 600 MHz) δ 0.57 (3H, s, H_3_-27), 0.97 (1H, m, H_2_-22β), 1.13 (3H, s, H_3_-28), 1.17 (3H, s, H_3_-30), 1.32 (3H, s, H-26), 1.32 (1H, m, H_2_-12″), 1.44 (1H, td, *J* = 13.5, 4.7 Hz, H_2_-21β), 1.48 (3H, s, H_3_-25), 1.50 (1H, m, H_2_-16a), 1.64 (1H, m, H_2_-15″), 1.66 (1H, m, H-18), 1.70 (1H, m, H_2_-15′), 1.74 (1H, m, H_2_-19b), 1.80 (2H, m, H_2_-12′, H_2_-11a), 1.95 (1H, td, *J* = 14.4, 6.4 Hz, H_2_-16β), 2.10 (1H, m, H_2_-22α), 2.18 (1H, d, *J* = 13.5 Hz, H_2_-21α), 2.21 (3H, s, H_3_-23), 2.24 (1H, d, *J* = 10.9 Hz, H_2_-11β), 2.47 (1H, d, *J* = 16.0 Hz, H_2_-19α), 3.56 (3H, s, OMe), 6.47 (2H, m, H-1, H-7), 7.21 (1H, dd, *J* = 7.2, 1.4 Hz, H-6); ^13^C NMR (CD_3_OD, 151 MHz) δ 10.3 (CH_3_-23), 19.2 (CH_3_-27), 22.1 (CH_3_-26), 29.7 (CH_2_-15), 30.7 (CH_2_-12), 30.9 (CH_2_-21), 31.4 (C-17), 31.9 (CH_2_-19), 32.0 (CH_3_-28), 33.0 (CH_3_-30), 34.6 (CH_2_-11), 35.9 (CH_2_-22), 37.6 (CH_2_-16), 38.9 (CH_3_-25), 40.7 (C-13), 41.6 (C-20), 44.2 (C-9), 45.7 (CH-18), 46.3 (C-14), 52.2 (OMe), 119.8 (CH-7), 120.1 (C-5), 120.6 (CH-1), 128.7, 136.3 (CH-6), 147.7 (C-3), 166.4 (C-10), 171.8 (C-8), 180.6 (C-29). For NMR spectra, see Supplementary Figures S10–S15. HRESIMS *m*/*z* 465.3002 (calculated for C_30_H_41_O_4_, error 0.69 ppm).

##### 4.5.13 Zeylasteral (**5**)^55^

Amorphous orange powder, [α]_D_^20^ +43 (c 0.0003, MeOH). UV (MeOH) λ_max_ 204 nm, 255 nm, 300 nm.

^1^H NMR (CDCl_3_, 600 MHz) δ 0.57 (3H, s, H_3_-27), 0.99 (2H, d, *J* = 14.1 Hz, H_2_-22β), 1.11 (3H, s, H_3_-28), 1.18 (3H, s, H_3_-30), 1.32 (3H, s, H_3_-26), 1.39 (1H, td, *J* = 14.1, 4.6 Hz, H_2_-21β), 1.56 (3H, s, H_3_-25), 1.58 (1H, m, H_2_-15b), 1.61 (1H, m, H-18), 1.68 (1H, m, H_2_-15a), 1.69 (1H, m, H_2_-16a), 1.70 (1H, dd, *J* = 15.9, 8.2 Hz, H_2_-19β), 1.73 (1H, m, H_2_-12b)1.82 (1H, ddd, *J* = 13.9, 5.3, 2.4 Hz, H_2_-12α), 1.89 (1H, m, H_2_-16b), 1.92 (1H, m, H_2_-11a), 2.05 (1H, td, *J* = 14.1, 4.2 Hz, H_2_-22α), 2.20 (1H, d, *J* = 14.1 Hz, H_2_-21α), 2.26 (1H, ddd, *J* = 13.6, 4.3, 2.4 Hz, H_2_-11β), 2.42 (1H, d, *J* = 15.9 Hz, H_2_-19α), 3.53 (3H, s, OMe), 6.19 (1H, s, 2OH), 6.34 (1H, s, H-7), 7.29 (1H, s, H-1), 11.04 (1H, s, H-23), 12.87 (1H, s, 3OH); ^1^H NMR (CDCl_3_, 151 MHz) δ 18.2 (CH_3_-27), 20.5 (CH_3_-26), 28.6 (CH_2_-15), 29.7 (CH_2_-12), 29.8 (CH_2_-21), 30.3 (C-17), 30.7 (CH_2_-19), 31.5 (CH_3_-28), 32.6 (CH_3_-30), 33.6 (CH_2_-11), 34.7 (CH_2_-22), 36.2 (CH_2_-16), 36.3 (CH_3_-25), 39.2 (C-13), 40.3 (C-20), 40.4 (C-9), 44.2 (CH-18), 44.9 (C-14), 51.4 (OMe), 116.1 (CH-1), 116.8 (C-4), 122.8 (C-5), 125.0 (CH-7), 148.9 (C-2), 149.3 (C-3), 150.2 (C-10), 173.8 (C-8), 178.6 (C-29), 200.1 (CH-23). For NMR spectra, see Supplementary Figures S16–S20. HRESIMS *m*/*z* 495.2754 (calculated for C_30_H_38_O_6_, error 2.69 ppm).

### 4.6 Plant Extracts Collection Bioactivity Assays

#### 4.6.1 Activity against *Leishmania donovani*

Amastigotes of *L. donovani* strain MHOM/ET/67/L82 were grown in axenic culture at 37 °C in SM medium^77^ at pH 5.4 supplemented with 10% heat-inactivated fetal bovine serum under an atmosphere of 5% CO_2_ in the air. 50 μL medium was added to each well of the 96-well microtiter plates. 50 μL of culture medium with 1 × 10^6^/mL amastigotes from axenic culture were added in 96-well microtiter plates. Extracts were dissolved in 5% DMSO at 0.2 mg/mL. Five μL and 1 μL of the sample solution respectively were added to the wells. The test concentrations were 10 μg/mL and 2 μg/mL. 50 μL of culture medium with 1 × 10^6^/mL amastigotes from axenic culture was added in 96-well microtiter plates. After 70 h of incubation, the plates were inspected under an inverted microscope to ensure growth of the controls and sterile conditions. Ten μL of Alamar Blue [12.5 mg resazurin dissolved in 100 mL distilled water,^78^] were then added to each well, and the plates were incubated for another 2 h. Then the plates were read with a Spectramax Gemini XS microplate fluorometer (Molecular Devices Corporation, San Jose, CA, USA) using an excitation wavelength of 536 nm and an emission wavelength of 588 nm. The data were evaluated in Excel. For each test concentration, the percent growth inhibition was calculated in comparison with an untreated control, and miltefosine at 10 μg/mL was included as positive control.

#### 4.6.2 Activity against *Trypanosoma brucei* Rhodesiense

This stock was isolated in 1982 from a human patient in Tanzania, and after several mouse passages cloned and adapted to axenic culture conditions.^79^ Minimum Essential Medium (50 μL) supplemented with 25 mM HEPES, 1 g/L additional glucose, 1% MEM nonessential amino acids (100×), 0.2 mM 2-mercaptoethanol, 1 mM sodium-pyruvate, and 15% heat-inactivated horse serum was added to each well of a 96-well microtiter plate. Extracts were dissolved in 5% DMSO at 0.2 mg/mL. Five μL and 1 μL of the sample solution respectively were added to the wells. The test concentrations were 10 μg/mL and 2 μg/mL. Then 4 × 10^3^ bloodstream forms of *T. b. rhodesiense* STIB 900 in 50 μL was added to each well, and the plate was incubated at 37 °C under a 5% CO_2_ atmosphere for 70 h. Ten μL resazurin solution (resazurin, 12.5 mg in 100 mL double-distilled water) was then added to each well, and incubation continued for a further 2–4 h.^80^ Then, the plates were read with a Spectramax Gemini XS microplate fluorometer (Molecular Devices Corporation) using an excitation wavelength of 536 nm and an emission wavelength of 588 nm. The data were evaluated in Excel. For each test concentration, the percent growth inhibition was calculated in comparison with an untreated control, and melarsoprol at 0.07 μg/mL was included as a positive control.

#### 4.6.3 Activity against *Trypanosoma cruzi*

Rat skeletal myoblasts (L-6 cells) were seeded in 96-well microtiter plates at 2000 cells/well in 100 μL RPMI 1640 medium with 10% FBS and 2 mM l-glutamine. After 24 h, the medium was removed and replaced by 100 μL per well containing 5000 trypomastigote forms of *T. cruzi* Tulahuen strain C2C4 containing the β-galactosidase (Lac Z) gene.^81^ After 48 h, the medium was removed from the wells and replaced by 100 μL fresh medium. Extracts were dissolved in 5% DMSO at 0.2 mg/mL. Five μL of the sample solution was added to the wells so that the test concentration was 10 μg/mL. After 96 h of incubation, the plates were inspected under an inverted microscope to ensure growth of the controls and sterility. Then the substrate CPRG/Nonidet (50 μL) was added to all wells. A color reaction developed within 2–6 h and could be read photometrically at 540 nm. The data were evaluated in Excel. For each test concentration, the percent growth inhibition was calculated in comparison with an untreated control, and benznidazole at 10 μg/mL was included as a positive control.

#### 4.6.4 Cytotoxicity Assay: L-6 Cells

Assays were performed in 96-well microtiter plates, each well containing 100 μL of RPMI 1640 medium supplemented with 1% l-glutamine (200 mM) and 10% fetal bovine serum, and 4000 L-6 cells (a primary cell line derived from rat skeletal myoblasts).^82,83^ Extracts were dissolved in 5% DMSO at 0.2 mg/mL. Five μL of the sample solution was added to the wells so that the test concentration was 10 μg/mL. After 70 h of incubation, the plates were inspected under an inverted microscope to ensure growth of the controls and sterile conditions. Ten μL of Alamar Blue was then added to each well, and the plates were incubated for another 2 h. Then the plates were read with a Spectramax Gemini XS microplate fluorometer (Molecular Devices Corporation) using an excitation wavelength of 536 nm and an emission wavelength of 588 nm. The data were evaluated in Excel. For each test concentration, the percent growth inhibition was calculated in comparison with an untreated control, and podophyllotoxin (Sigma P4405) at 0.1 μg/mL was included as a positive control.

### 4.7 Pure Compounds Bioactivity Assays

#### 4.7.1 Cytotoxicity Assay: L-6 Cells

Assays were performed in 96-well microtiter plates, each well containing 100 μL of RPMI 1640 medium supplemented with 1% l-glutamine (200 mM) and 10% fetal bovine serum, and 4000 L-6 cells (a primary cell line derived from rat skeletal myoblasts).^82,83^ Serial drug dilutions of eleven 3-fold dilution steps covering a range from 100 to 0.002 μg/mL were prepared. After 70 h of incubation, the plates were inspected under an inverted microscope to ensure the growth of the controls and sterile conditions. 10 μL of resazurin was then added to each well, and the plates were incubated for another 2 h. Then the plates were read with a Spectramax Gemini XS microplate fluorometer (Molecular Devices Corporation, Sunnyvale, CA, USA) using an excitation wavelength of 536 nm and an emission wavelength of 588 nm. The IC_50_ values were calculated by linear regression (Huber 1993) and 4-parameter logistic regression from the sigmoidal dose inhibition curves using SoftmaxPro software (Molecular Devices Corporation, Sunnyvale, CA, USA). Podophyllotoxin (Sigma P4405) is used as a control.

#### 4.7.2 Activity against *Leishmania donovani*

Amastigotes of *L. donovani* strain MHOM/ET/67/L82 were grown in axenic culture at 37 °C in SM medium^77^ at pH 5.4 supplemented with 10% heat-inactivated fetal bovine serum under an atmosphere of 5% CO_2_ in air. One hundred microliters of culture medium with 105 amastigotes from axenic culture with or without a serial drug dilution were seeded in 96-well microtiter plates. Serial drug dilutions of eleven 3-fold dilution steps covering a range from 100 to 0.002 μg/mL were prepared. After 70 h of incubation, the plates were inspected under an inverted microscope to ensure growth of the controls and sterile conditions. Ten μL of resazurin (12.5 mg resazurin dissolved in 100 mL distilled water) were then added to each well, and the plates were incubated for another 2 h. Then the plates are read with a Spectramax Gemini XS microplate fluorometer (Molecular Devices Corporation, Sunnyvale, CA, USA) using an excitation wavelength of 536 nm and an emission wavelength of 588 nm. From the sigmoidal inhibition curves, the IC_50_ values were calculated by linear regression^84^ and 4-parameter logistic regression using SoftmaxPro software (Molecular Devices Corporation, Sunnyvale, CA, USA).

#### 4.7.3 Activity against *Trypanosoma cruzi*

Rat skeletal myoblasts (L-6 cells) were seeded in 96-well microtiter plates at 2000 cells/well in 100 μL RPMI 1640 medium with 10% FBS and 2 mM l-glutamine. After 24 h,the medium was removed and replaced by 100 μL per well containing 5000 trypomastigote forms of *T. cruzi* Tulahuen strain C2C4 containing the β-galactosidase (Lac Z) gene.^81^ After 48 h, the medium was removed from the wells and replaced by 100 μL fresh medium with or without a serial drug dilution of eleven 3-fold dilution steps covering a range from 100 to 0.002 μg/mL. After 96 h of incubation, the plates were inspected under an inverted microscope to ensure the growth of the controls and sterility. Then the substrate CPRG/Nonidet (50 μL) was added to all wells. A color reaction developed within 2–6 h and could be read photometrically at 540 nm. Data were analyzed with the graphic program Softmax Pro (Molecular Devices), which calculated IC_50_ values by linear regression^84^ and 4-parameter logistic regression from the sigmoidal dose inhibition curves. Benznidazole was used as a control (IC_50_ 0.5 ± 0.2 g/mL).

## Data Availability

All the scripts used to process the data are available on different repositories available at https://github.com/enpkg. The different versions and parameters used are described in Materials and Methods. The raw LC-MS^2^ data and the features’ MS^2^ spectra are available on the MASSive repository: MSV000087728, MSV000088521, and MSV000093464 for the 1,600 extracts collection, *Waltheria* samples, and Korean medicinal plants, respectively. The samples’ individual. ttl files are available on Zenodo. For the 1,600 extracts collection, data sets were uploaded in 20 batches - each batch corresponding to a given 96-well plate: plate 138, record 10284426; plate 139, record 10284425; plate 140, record 10284422; plate 141, record 10284417; plate 142, record 10284416; plate 143, record 10284413; plate 144, record 10284412; plate 145, record 10284409; plate 146, record 10284408; plate 147, record 10284405; plate 150, record 10282160; plate 151, record 10282147; plate 152, record 10282126; plate 153, record 10282138; plate 154, record 10282116; plate 155, record 10282099; plate 156, record 10282088; plate 157, record 10282003; plate 158, record 10282073; plate 159, record 10282053. The 337 Korean medicinal plants data set was uploaded in 4 batches, MSV000093464_batch_01, record 10285076; MSV000093464_batch_02, record 10285383; MSV000093464_batch_03, record 10285452; and MSV000093464_batch_04, record 10285516. *Waltheria* data are available at 8038719, ChEMBL data at 7953284, and the graph schemes (*enpkg* and *enpkg_module*) at 8079700. The scripts used to generate the figures are available athttps://github.com/ArnaudGaudry/enpkg_publication_examples.

## Supporting Information Available

The supporting material contains The Supporting Information is available free of charge at https://pubs.acs.org/doi/10.1021/acscentsci.3c00800.

A schematic view of the Experimental Natural Products Knowledge Graph (KG) data model (S1), additional scatter plots for the anti-*T. cruzi* chapter (S2–S3), NMR spectra of the isolated compounds (S4–S20), and a schematic overview of the annotation workflow applied in the ENPKG framework (S21) (PDF)
Transparent Peer Review report available (PDF)

## References

[ref1] David B.; Wolfender J.-L.; Dias D. A. The pharmaceutical industry and natural products: historical status and new trends. Phytochem Rev. 2015, 14, 299–315. 10.1007/s11101-014-9367-z.

[ref2] Newman D. J.; Cragg G. M. Natural Products as Sources of New Drugs over the Nearly Four Decades from 01/1981 to 09/2019. J. Nat. Prod. 2020, 83, 770–803. 10.1021/acs.jnatprod.9b01285. 32162523

[ref3] Feher M.; Schmidt J. M. Property Distributions: Differences between Drugs, Natural Products, and Molecules from Combinatorial Chemistry. J. Chem. Inf. Comput. Sci. 2003, 43, 218–227. 10.1021/ci0200467. 12546556

[ref4] Clemons P. A.; Bodycombe N. E.; Carrinski H. A.; Wilson J. A.; Shamji A. F.; Wagner B. K.; et al. Small molecules of different origins have distinct distributions of structural complexity that correlate with protein-binding profiles. Proc. Natl. Acad. Sci. U. S. A. 2010, 107, 18787–18792. 10.1073/pnas.1012741107. 20956335 PMC2973913

[ref5] Bindseil K. U.; Jakupovic J.; Wolf D.; Lavayre J.; Leboul J.; van der Pyl D. Pure compound libraries; a new perspective for natural product based drug discovery. Drug Discov Today. 2001, 6, 840–847. 10.1016/S1359-6446(01)01856-6. 11495757

[ref6] Sukuru S. C. K.; Jenkins J. L.; Beckwith R. E. J.; Scheiber J.; Bender A.; Mikhailov D.; et al. Plate-based diversity selection based on empirical HTS data to enhance the number of hits and their chemical diversity. J. Biomol Screen. 2009, 14, 690–699. 10.1177/1087057109335678. 19531667

[ref7] Stratton C. F.; Newman D. J.; Tan D. S. Cheminformatic comparison of approved drugs from natural product versus synthetic origins. Bioorg. Med. Chem. Lett. 2015, 25, 4802–4807. 10.1016/j.bmcl.2015.07.014. 26254944 PMC4607632

[ref8] Wolfender J.-L.; Litaudon M.; Touboul D.; Queiroz E. F. Innovative omics-based approaches for prioritisation and targeted isolation of natural products - new strategies for drug discovery. Nat. Prod Rep. 2019, 36, 855–868. 10.1039/C9NP00004F. 31073562

[ref9] Nothias L.-F.; Petras D.; Schmid R.; Dührkop K.; Rainer J.; Sarvepalli A.; et al. Feature-based molecular networking in the GNPS analysis environment. Nat. Methods. 2020, 17, 905–908. 10.1038/s41592-020-0933-6. 32839597 PMC7885687

[ref10] Wang M.; Carver J. J.; Phelan V. V.; Sanchez L. M.; Garg N.; Peng Y.; et al. Sharing and community curation of mass spectrometry data with GNPS. Nat. Biotechnol. 2016, 34, 828–837. 10.1038/nbt.3597. 27504778 PMC5321674

[ref11] Watrous J.; Roach P.; Alexandrov T.; Heath B. S.; Yang J. Y.; Kersten R. D.; et al. Mass spectral molecular networking of living microbial colonies. Proc. Natl. Acad. Sci. U. S. A. 2012, 109, E1743–52. 10.1073/pnas.1203689109. 22586093 PMC3387089

[ref12] Olivon F.; Allard P.-M.; Koval A.; Righi D.; Genta-Jouve G.; Neyts J.; et al. Bioactive Natural Products Prioritization Using Massive Multi-informational Molecular Networks. ACS Chem. Biol. 2017, 12, 2644–2651. 10.1021/acschembio.7b00413. 28829118

[ref13] Rodríguez-Coira J.; Delgado-Dolset M. I.; Obeso D.; Dolores-Hernández M.; Quintás G.; Angulo S. Troubleshooting in Large-Scale LC-ToF-MS Metabolomics Analysis: Solving Complex Issues in Big Cohorts. Metabolites. 2019, 9, 247 10.3390/metabo9110247. 31652940 PMC6918290

[ref14] Gaudry A.; Huber F.; Nothias L.-F.; Cretton S.; Kaiser M.; Wolfender J.-L., MEMO: Mass Spectrometry-Based Sample Vectorization to Explore Chemodiverse Datasets. Frontiers in Bioinformatics. 2022, 2, 10.3389/fbinf.2022.842964. PMC9580960 36304329

[ref15] RDF - Semantic Web Standards. [cited 25 Aug 2022]. Available: https://www.w3.org/RDF/.

[ref16] SPARQL Query Language for RDF. [cited 25 Aug 2022]. Available: https://www.w3.org/TR/rdf-sparql-query/.

[ref17] Ahmad I. A. H.; Losacco G. L.; Shchurik V.; Wang X.; Cohen R. D.; Herron A. N.; et al. Trapping-enrichment multi-dimensional liquid chromatography with on-line deuterated solvent exchange for streamlined structure elucidation at the microgram scale. Angew. Chem., Int. Ed. Engl. 2022, 61, e202117655 10.1002/anie.202117655. 35139257

[ref18] Turki H.; Shafee T.; Hadj Taieb M. A.; Ben Aouicha M.; Vrandečić D.; Das D.; et al. Wikidata: A large-scale collaborative ontological medical database. J. Biomed Inform. 2019, 99, 103292 10.1016/j.jbi.2019.103292. 31557529

[ref19] Rutz A.; Sorokina M.; Galgonek J.; Mietchen D.; Willighagen E.; Gaudry A., The LOTUS initiative for open knowledge management in natural products research. Elife 2022, 11, 10.7554/eLife.70780. PMC9135406 35616633

[ref20] Waagmeester A.; Stupp G.; Burgstaller-Muehlbacher S.; Good B. M.; Griffith M.; Griffith O. L., Wikidata as a knowledge graph for the life sciences. Elife 2020, 9, 10.7554/eLife.52614. PMC7077981 32180547

[ref21] Zeng X.; Tu X.; Liu Y.; Fu X.; Su Y. Toward better drug discovery with knowledge graph. Curr. Opin Struct Biol. 2022, 72, 114–126. 10.1016/j.sbi.2021.09.003. 34649044

[ref22] Himmelstein D. S.; Lizee A.; Hessler C.; Brueggeman L.; Chen S. L.; Hadley D., Systematic integration of biomedical knowledge prioritizes drugs for repurposing. Elife 2017, 6, 10.7554/eLife.26726. PMC5640425 28936969

[ref23] Santos A.; Colaco A. R.; Nielsen A. B.; Niu L.; Strauss M.; Geyer P. E.; Coscia F.; Albrechtsen N. J. W.; Mundt F.; Jensen L. J.; Mann M. A knowledge graph to interpret clinical proteomics data. Nat. Biotechnol. 2022, 40, 692–702. 10.1038/s41587-021-01145-6. 35102292 PMC9110295

[ref24] Kang K. B.; Jeong E.; Son S.; Lee E.; Lee S.; Choi S. Y.; et al. Mass spectrometry data on specialized metabolome of medicinal plants used in East Asian traditional medicine. Sci. Data. 2022, 9, 528 10.1038/s41597-022-01662-2. 36030263 PMC9420114

[ref25] Shannon P.; Markiel A.; Ozier O.; Baliga N. S.; Wang J. T.; Ramage D.; et al. Cytoscape: a software environment for integrated models of biomolecular interaction networks. Genome Res. 2003, 13, 2498–2504. 10.1101/gr.1239303. 14597658 PMC403769

[ref26] Pluskal T.; Castillo S.; Villar-Briones A.; Orešič M. MZmine 2: Modular framework for processing, visualizing, and analyzing mass spectrometry-based molecular profile data. BMC Bioinformatics. 2010, 11, 395 10.1186/1471-2105-11-395. 20650010 PMC2918584

[ref27] Deutsch E. W.; Perez-Riverol Y.; Carver J.; Kawano S.; Mendoza L.; Van Den Bossche T.; et al. Universal Spectrum Identifier for mass spectra. Nat. Methods. 2021, 18, 768–770. 10.1038/s41592-021-01184-6. 34183830 PMC8405201

[ref28] Petras D.; Phelan V. V.; Acharya D.; Allen A. E.; Aron A. T.; Bandeira N.; et al. GNPS Dashboard: collaborative exploration of mass spectrometry data in the web browser. Nat. Methods. 2022, 19, 134–136. 10.1038/s41592-021-01339-5. 34862502 PMC8831450

[ref29] Jarmusch A. K.; Wang M.; Aceves C. M.; Advani R. S.; Aguirre S.; Aksenov A. A.; et al. ReDU: a framework to find and reanalyze public mass spectrometry data. Nat. Methods. 2020, 17, 901 10.1038/s41592-020-0916-7. 32807955 PMC7968862

[ref30] Hinchliff C. E.; Smith S. A.; Allman J. F.; Burleigh J. G.; Chaudhary R.; Coghill L. M.; et al. Synthesis of phylogeny and taxonomy into a comprehensive tree of life. Proc. Natl. Acad. Sci. U. S. A. 2015, 112, 12764–12769. 10.1073/pnas.1423041112. 26385966 PMC4611642

[ref31] Mctavish E. J.; Sánchez-Reyes L. L.; Holder M. T. OpenTree: A Python Package for Accessing and Analyzing Data from the Open Tree of Life. Syst. Biol. 2021, 70, 1295–1301. 10.1093/sysbio/syab033. 33970279 PMC8513759

[ref32] Huber F.; Verhoeven S.; Meijer C.; Spreeuw H.; Castilla E.; Geng C.; et al. Matchms - processing and similarity evaluation of mass spectrometry data. J. Open Source Softw. 2020, 5, 2411 10.21105/joss.02411.

[ref33] Allard P.-M.; Péresse T.; Bisson J.; Gindro K.; Marcourt L.; Pham V. C.; et al. Integration of Molecular Networking and In-Silico MS/MS Fragmentation for Natural Products Dereplication. Anal. Chem. 2016, 88, 3317–3323. 10.1021/acs.analchem.5b04804. 26882108

[ref34] Rutz A.; Dounoue-Kubo M.; Ollivier S.; Bisson J., Bagheri M.; Saesong T., Taxonomically Informed Scoring Enhances Confidence in Natural Products Annotation. Front Plant Sci. 2019, 10, 10.3389/fpls.2019.01329. PMC6824209 31708947

[ref35] Allard P.-M.; Bisson J.; Rutz A. ISDB: In Silico spectral databases of natural products. Zenodo, 2022, 10.5281/ZENODO.7534250

[ref36] Duhrkop K.; Fleischauer M.; Ludwig M.; Aksenov A. A.; Melnik A. V.; Meusel M.; et al. SIRIUS 4: a rapid tool for turning tandem mass spectra into metabolite structure information. Nat. Methods. 2019, 16, 299–302. 10.1038/s41592-019-0344-8. 30886413

[ref37] Duhrkop K.; Shen H.; Meusel M.; Rousu J.; Bocker S. Searching molecular structure databases with tandem mass spectra using CSI:FingerID. Proc. Natl. Acad. Sci. U. S. A. 2015, 112, 12580–12585. 10.1073/pnas.1509788112. 26392543 PMC4611636

[ref38] Ludwig M.; Nothias L.-F.; Dührkop K.; Koester I.; Fleischauer M.; Hoffmann M. A.; et al. Database-independent molecular formula annotation using Gibbs sampling through ZODIAC. Nature Machine Intelligence. 2020, 2, 629–641. 10.1038/s42256-020-00234-6.

[ref39] Hoffmann M. A.; Nothias L.-F.; Ludwig M.; Fleischauer M.; Gentry E. C.; Witting M.; et al. High-confidence structural annotation of metabolites absent from spectral libraries. Nat. Biotechnol. 2022, 40, 411–421. 10.1038/s41587-021-01045-9. 34650271 PMC8926923

[ref40] Dührkop K.; Nothias L.-F.; Fleischauer M.; Reher R.; Ludwig M.; Hoffmann M. A.; et al. Systematic classification of unknown metabolites using high-resolution fragmentation mass spectra. Nat. Biotechnol. 2021, 39, 462–471. 10.1038/s41587-020-0740-8. 33230292

[ref41] Huber F.; Ridder L.; Verhoeven S.; Spaaks J. H.; Diblen F.; Rogers S.; et al. Spec2Vec: Improved mass spectral similarity scoring through learning of structural relationships. PLoS Comput. Biol. 2021, 17, e1008724 10.1371/journal.pcbi.1008724. 33591968 PMC7909622

[ref42] Kim H. W.; Wang M.; Leber C. A.; Nothias L.-F.; Reher R.; Kang K. B.; et al. NPClassifier: A Deep Neural Network-Based Structural Classification Tool for Natural Products. J. Nat. Prod. 2021, 84, 2795–2807. 10.1021/acs.jnatprod.1c00399. 34662515 PMC8631337

[ref43] Boettiger C.; Ooms J.; pdatascience; Leinweber K.; Hester J. ropensci/rdflib, v0.2.3, 2020. 10.5281/zenodo.3604372.

[ref44] Cretton S.; Dorsaz S.; Azzollini A.; Favre-Godal Q.; Marcourt L.; Ebrahimi S. N.; et al. Antifungal Quinoline Alkaloids from Waltheria indica. J. Nat. Prod. 2016, 79, 300–307. 10.1021/acs.jnatprod.5b00896. 26848627

[ref45] Cretton S.; Bréant L.; Pourrez L.; Ambuehl C.; Perozzo R.; Marcourt L.; et al. Chemical constituents from Waltheria indica exert in vitro activity against Trypanosoma brucei and T. cruzi. Fitoterapia. 2015, 105, 55–60. 10.1016/j.fitote.2015.06.007. 26072041

[ref46] McInnes L.; Healy J.; Melville J. UMAP: Uniform Manifold Approximation and Projection for Dimension Reduction. arXiv [stat.ML] 2018. Available: http://arxiv.org/abs/1802.03426.

[ref47] Probst D.; Reymond J.-L. Visualization of very large high-dimensional data sets as minimum spanning trees. J. Cheminform. 2020, 12, 12 10.1186/s13321-020-0416-x. 33431043 PMC7015965

[ref48] Hernandez F. R.; Turrens J. F. Rotenone at high concentrations inhibits NADH-fumarate reductase and the mitochondrial respiratory chain of Trypanosoma brucei and T. cruzi. Mol. Biochem. Parasitol. 1998, 93, 135–137. 10.1016/S0166-6851(98)00015-2. 9662035

[ref49] Affranchino J. L.; De Tarlovsky M. N.; Stoppani A. O. Respiratory control in mitochondria from Trypanosoma cruzi. Mol. Biochem. Parasitol. 1985, 16, 289–298. 10.1016/0166-6851(85)90071-4. 3903495

[ref50] Queiroz E. F.; Alfattani A.; Afzan A.; Marcourt L.; Guillarme D.; Wolfender J. L. Utility of dry load injection for an efficient natural products isolation at the semi-preparative chromatographic scale. J. Chromatogr A 2019, 1598, 85 10.1016/j.chroma.2019.03.042. 30926257

[ref51] Kratochvíl M.; Vondrášek J.; Galgonek J. Sachem: a chemical cartridge for high-performance substructure search. J. Cheminform. 2018, 10, 27 10.1186/s13321-018-0282-y. 29797000 PMC5966370

[ref52] Thiem D. A.; Sneden A. T.; Khan S. I.; Tekwani B. L. Bisnortriterpenes from Salacia madagascariensis. J. Nat. Prod. 2005, 68, 251–254. 10.1021/np0497088. 15730255

[ref53] Khalid S. A.; Tahir A. E.; Satti G. M.; Friedrichsen G. M.; Christensen S. B. Isolation and characterization of pristimerin as the antiplasmodial and antileishmanial agent of Maytenus senegalensis (Lam.) Exell. ARKIVOC 2007, 2007, 129–134. 10.3998/ark.5550190.0008.915.

[ref54] Jarmusch A. K.; Aron A. T.; Petras D.; Phelan V. V.; Bittremieux W., Acharya D. D., A Universal Language for Finding Mass Spectrometry Data Patterns. bioRxiv, 2022, p 2022.08.06.503000. 10.1101/2022.08.06.503000. PMC12334354 40355727

[ref55] Gamlath C. B.; Gunaherath K. B.; Gunatilaka A. A. L. Studies on terpenoids and steroids. Part 10. Structures of four new natural phenol is D:A-friedo-24-noroleanane triterpenoids. J. Chem. Soc. Perkin 1. 1987, 2849–2854. 10.1039/p19870002849.

[ref56] Niampoka C.; Suttisri R.; Bavovada R.; Takayama H.; Aimi N. Potentially cytotoxic triterpenoids from the root bark of Siphonodon celastrineus Griff. Arch Pharm. Res. 2005, 28, 546–549. 10.1007/BF02977756. 15974440

[ref57] Espindola L. S.; Dusi R. G.; Demarque D. P.; Braz-Filho R.; Yan P.; Bokesch H. R.; et al. Cytotoxic Triterpenes from Salacia crassifolia and Metabolite Profiling of Celastraceae Species. Molecules. 2018, 23, 1494 10.3390/molecules23061494. 29925807 PMC6099938

[ref58] dos Santos V.; Leite K.; da Costa Siqueira M.; Regasini L.; Martinez I.; Nogueira C.; Galuppo M.; Stolf B.; Pereira A.; Cicarelli R.; Furlan M.; Graminha M. Antiprotozoal activity of quinonemethide triterpenes from Maytenus ilicifolia (Celastraceae). Molecules. 2013, 18, 1053–1062. 10.3390/molecules18011053. 23322069 PMC6270509

[ref59] Jarmusch S. A.; van der Hooft J. J. J.; Dorrestein P. C.; Jarmusch A. K. Advancements in capturing and mining mass spectrometry data are transforming natural products research. Nat. Prod Rep. 2021, 38, 2066 10.1039/D1NP00040C. 34612288 PMC8667781

[ref60] Wang M.; Jarmusch A. K.; Vargas F.; Aksenov A. A.; Gauglitz J. M.; Weldon K.; et al. Mass spectrometry searches using MASST. Nat. Biotechnol. 2020, 38, 23 10.1038/s41587-019-0375-9. 31894142 PMC7236533

[ref61] Claeys T.; Van Den Bossche T.; Perez-Riverol Y.; Gevaert K.; Vizcaíno J. A.; Martens L. lesSDRF is more: maximizing the value of proteomics data through streamlined metadata annotation. Nat. Commun. 2023, 14, 1–4. 10.1038/s41467-023-42543-5. 37875519 PMC10598006

[ref62] Smith C. A.; Want E. J.; O’Maille G.; Abagyan R.; Siuzdak G. XCMS: processing mass spectrometry data for metabolite profiling using nonlinear peak alignment, matching, and identification. Anal. Chem. 2006, 78, 779–787. 10.1021/ac051437y. 16448051

[ref63] Li S.; Siddiqa A.; Thapa M.; Chi Y.; Zheng S. Trackable and scalable LC-MS metabolomics data processing using asari. Nat. Commun. 2023, 14, 4113 10.1038/s41467-023-39889-1. 37433854 PMC10336130

[ref64] Tsugawa H.; Cajka T.; Kind T.; Ma Y.; Higgins B.; Ikeda K.; et al. MS-DIAL: data-independent MS/MS deconvolution for comprehensive metabolome analysis. Nat. Methods. 2015, 12, 523–526. 10.1038/nmeth.3393. 25938372 PMC4449330

[ref65] Djoumbou Feunang Y.; Eisner R.; Knox C.; Chepelev L.; Hastings J.; Owen G.; et al. ClassyFire: automated chemical classification with a comprehensive, computable taxonomy. J. Cheminform. 2016, 8, 61 10.1186/s13321-016-0174-y. 27867422 PMC5096306

[ref66] Sima A. C.; Mendes de Farias T.; Anisimova M.; Dessimoz C.; Robinson-Rechavi M.; Zbinden E.; et al. Bio-SODA UX: enabling natural language question answering over knowledge graphs with user disambiguation. Distrib Parallel Databases. 2022, 40, 409–440. 10.1007/s10619-022-07414-w. 36097541 PMC9458692

[ref67] Chambers M. C.; Maclean B.; Burke R.; Amodei D.; Ruderman D. L.; Neumann S.; et al. A cross-platform toolkit for mass spectrometry and proteomics. Nat. Biotechnol. 2012, 30, 918–920. 10.1038/nbt.2377. 23051804 PMC3471674

[ref68] Myers O. D.; Sumner S. J.; Li S.; Barnes S.; Du X. One Step Forward for Reducing False Positive and False Negative Compound Identifications from Mass Spectrometry Metabolomics Data: New Algorithms for Constructing Extracted Ion Chromatograms and Detecting Chromatographic Peaks. Anal. Chem. 2017, 89, 8696–8703. 10.1021/acs.analchem.7b00947. 28752754

[ref69] Wang F.; Liigand J.; Tian S.; Arndt D.; Greiner R.; Wishart D. S. CFM-ID 4.0: More Accurate ESI-MS/MS Spectral Prediction and Compound Identification. Anal. Chem. 2021, 93, 11692 10.1021/acs.analchem.1c01465. 34403256 PMC9064193

[ref70] da Silva R. R.; Wang M.; Nothias L.-F.; van der Hooft J. J. J.; Caraballo-Rodriguez A. M.; Fox E.; Balunas M. J.; Klassen J. L.; Lopes N. P.; Dorrestein P. C. Propagating annotations of molecular networks using in silico fragmentation. PLoS Comput. Biol. 2018, 14, e1006089 10.1371/journal.pcbi.1006089. 29668671 PMC5927460

[ref71] Gaulton A.; Bellis L. J.; Bento A. P.; Chambers J.; Davies M.; Hersey A.; et al. ChEMBL: a large-scale bioactivity database for drug discovery. Nucleic Acids Res. 2012, 40, D1100–7. 10.1093/nar/gkr777. 21948594 PMC3245175

[ref72] Davies M.; Nowotka M.; Papadatos G.; Dedman N.; Gaulton A.; Atkinson F.; et al. ChEMBL web services: streamlining access to drug discovery data and utilities. Nucleic Acids Res. 2015, 43, W612–20. 10.1093/nar/gkv352. 25883136 PMC4489243

[ref73] RDKit: Open-source cheminformatics. Available: https://www.rdkit.org/.

[ref74] Ertl P.; Roggo S.; Schuffenhauer A. Natural product-likeness score and its application for prioritization of compound libraries. J. Chem. Inf Model. 2008, 48, 68–74. 10.1021/ci700286x. 18034468

[ref75] Allard P.-M.; Gaudry A.; Quirós-Guerrero L.-M.; Rutz A.; Dounoue-Kubo M.; Walker T. W. N., Open and reusable annotated mass spectrometry dataset of a chemodiverse collection of 1,600 plant extracts. Gigascience 2022, 12, 10.1093/gigascience/giac124. PMC9845059 36649739

[ref76] European Commission. Official European Commission register of collections. 2020. Available: https://ec.europa.eu/environment/nature/biodiversity/international/abs/pdf/Register%20of%20Collections.pdf.

[ref77] Cunningham I. New culture medium for maintenance of tsetse tissues and growth of trypanosomatids. J. Protozool. 1977, 24, 325–329. 10.1111/j.1550-7408.1977.tb00987.x. 881656

[ref78] Mikus J.; Steverding D. A simple colorimetric method to screen drug cytotoxicity against Leishmania using the dye Alamar Blue. Parasitol Int. 2000, 48, 265–269. 10.1016/S1383-5769(99)00020-3. 11227767

[ref79] Baltz T.; Baltz D.; Giroud C.; Crockett J. Cultivation in a semi-defined medium of animal infective forms of Trypanosoma brucei, T. equiperdum, T. evansi, T. rhodesiense and T. gambiense. EMBO J. 1985, 4, 1273–1277. 10.1002/j.1460-2075.1985.tb03772.x. 4006919 PMC554336

[ref80] Räz B.; Iten M.; Grether-Bühler Y.; Kaminsky R.; Brun R. The Alamar Blue assay to determine drug sensitivity of African trypanosomes (T.b. rhodesiense and T.b. gambiense) in vitro. Acta Trop. 1997, 68, 139–147. 10.1016/S0001-706X(97)00079-X. 9386789

[ref81] Buckner F. S.; Verlinde C. L.; La Flamme A. C.; Van Voorhis W. C. Efficient technique for screening drugs for activity against Trypanosoma cruzi using parasites expressing beta-galactosidase. Antimicrob. Agents Chemother. 1996, 40, 2592–2597. 10.1128/AAC.40.11.2592. 8913471 PMC163582

[ref82] Page B.; Page M.; Noel C. A new fluorometric assay for cytotoxicity measurements in-vitro. Int. J. Oncol. 1993, 3, 473–476. 10.3892/ijo.3.3.473. 21573387

[ref83] Ahmed S. A.; Gogal R. M. Jr; Walsh J. E. A new rapid and simple non-radioactive assay to monitor and determine the proliferation of lymphocytes: an alternative to [3H]thymidine incorporation assay. J. Immunol Methods. 1994, 170, 211–224. 10.1016/0022-1759(94)90396-4. 8157999

[ref84] Huber W.; Koella J. C. A comparison of three methods of estimating EC50 in studies of drug resistance of malaria parasites. Acta Trop. 1993, 55, 257–261. 10.1016/0001-706X(93)90083-N. 8147282

