# Supplementary material for: A Sample-Centric and Knowledge-Driven Computational Framework for Natural Products Drug Discovery
Source: ACS Cent Sci. 2024 Feb 20;10(3):494–510. doi: 10.1021/acscentsci.3c00800 (PMC10979503; doi:10.1021/acscentsci.3c00800)
Supplement: Supplementary file 1 — oc3c00800_si_001.pdf [file oc3c00800_si_001.pdf]

Supporting Informations: A Sample-Centric and Knowledge-Driven Computational Framework for Natural Products Drug Discovery

Arnaud Gaudry[1,2], arnaud.gaudry@unige.ch, 0000-0002-3648-7362
Marco Pagni[3], marco.pagni@sib.swiss, 0000-0001-9292-9463
Florence Mehl[3], florence.mehl@sib.swiss, 0000-0002-9619-1707
Sébastien Moretti[3], sebastien.moretti@sib.swiss, 0000-0003-3947-488X
Luis-Manuel Quiros-Guerrero[1,2], luis.guerrero@unige.ch, 0000-0002-1630-8697
Luca Cappelletti[8], luca.cappelletti@unifr.ch, 0000-0002-1269-2038
Adriano Rutz[1,2], rutz@imsb.biol.ethz.ch, 0000-0003-0443-9902
Marcel Kaiser[4,5], marcel.kaiser@swisstph.ch, 0000-0003-1785-7302
Laurence Marcourt[1,2], laurence.marcourt@unige.ch, 0000-0002-9614-1099
Emerson Ferreira Queiroz[1,2], emerson.ferreira@unige.ch, 0000-0001-9567-1664
Jean-Robert Ioset[6], jrioset@dndi.org, No ORCID
Antonio Grondin[7], antonio.grondin@pierre-fabre.com, 0000-0002-2952-6271
Bruno David[7], brunoxdavid@gmail.com, 0000-0002-6222-9228
Jean-Luc Wolfender[1,2], jean-luc.wolfender@unige.ch, 0000-0002-0125-952X
Pierre-Marie Allard[1,2,8], pierre-marie.allard@unifr.ch, 0000-0003-3389-2191

[1] Institute of Pharmaceutical Sciences of Western Switzerland, University of Geneva, 1211 Geneva 4, Switzerland.
[2] School of Pharmaceutical Sciences, University of Geneva, 1211 Geneva 4, Switzerland.
[3] Vital-IT, SIB Swiss Institute of Bioinformatics, 1015 Lausanne, Switzerland.
[4] Department of Medical and Parasitology and Infection Biology, Swiss Tropical and Public Health Institute, 4123 Allschwil, Switzerland.
[5] Faculty of Science, University of Basel, 4002 Basel, Switzerland.
[6] Drugs for Neglected Diseases Initiative (DNDi), 1202 Geneva, Switzerland.
[7] Green Mission Pierre Fabre, Institut de Recherche Pierre Fabre, 31562 Toulouse, France.
[8] Department of Biology, University of Fribourg, 1700 Fribourg, Switzerland.

Supplementary Material

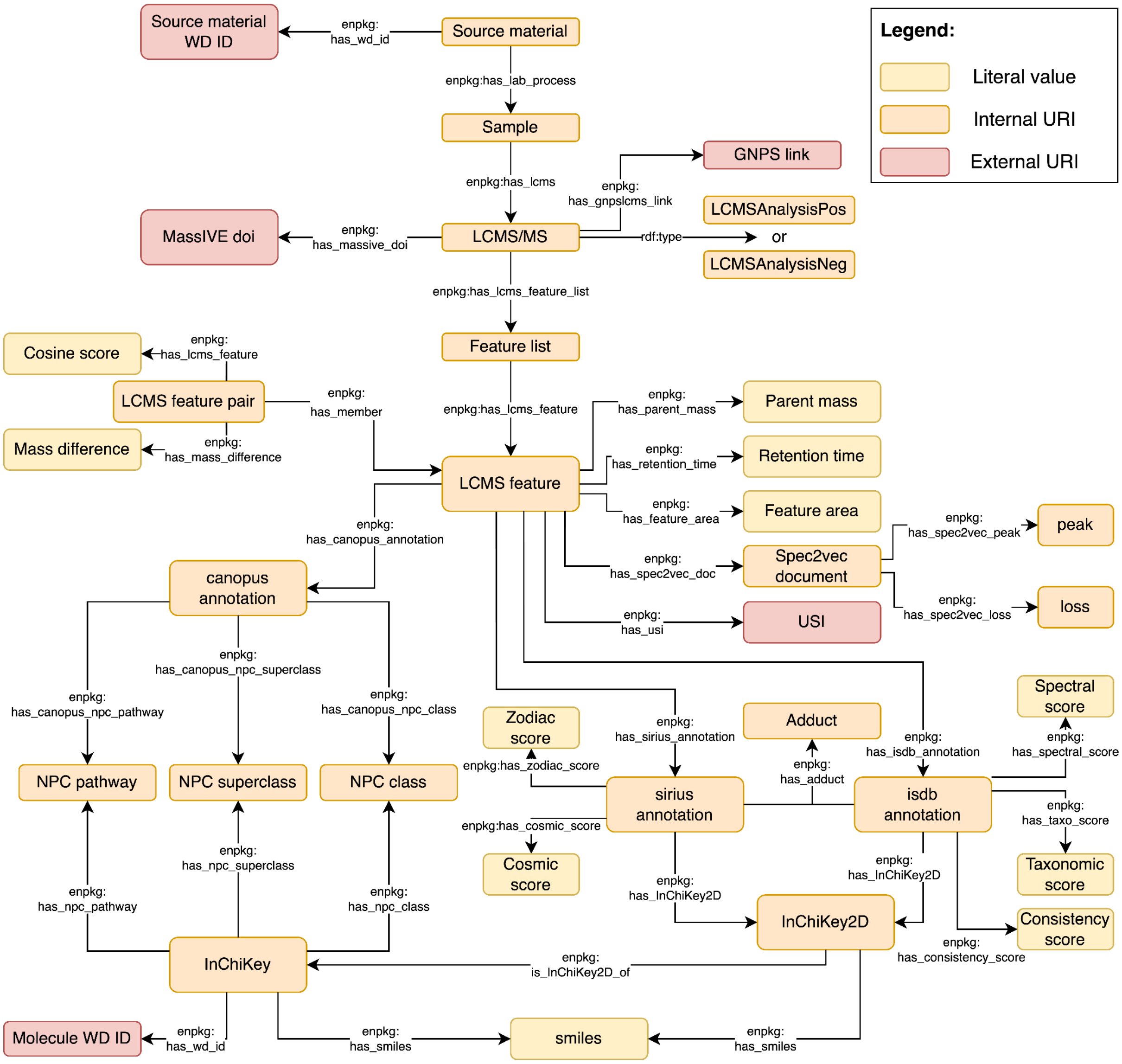

Figure S1: The generated Experimental Natural Products Knowledge Graph (KG) data model. The main object classes are represented.

> SPARQL query: For each NPClassifier chemical class, what is the average count of CANOPUS annotations (p>0.5) in each sample for the whole dataset and the cluster of active samples?

Returns: 152 chemical classes with at least 1 annotation in the selection, with the average CANOPUS annotation count in the whole set and in the selection

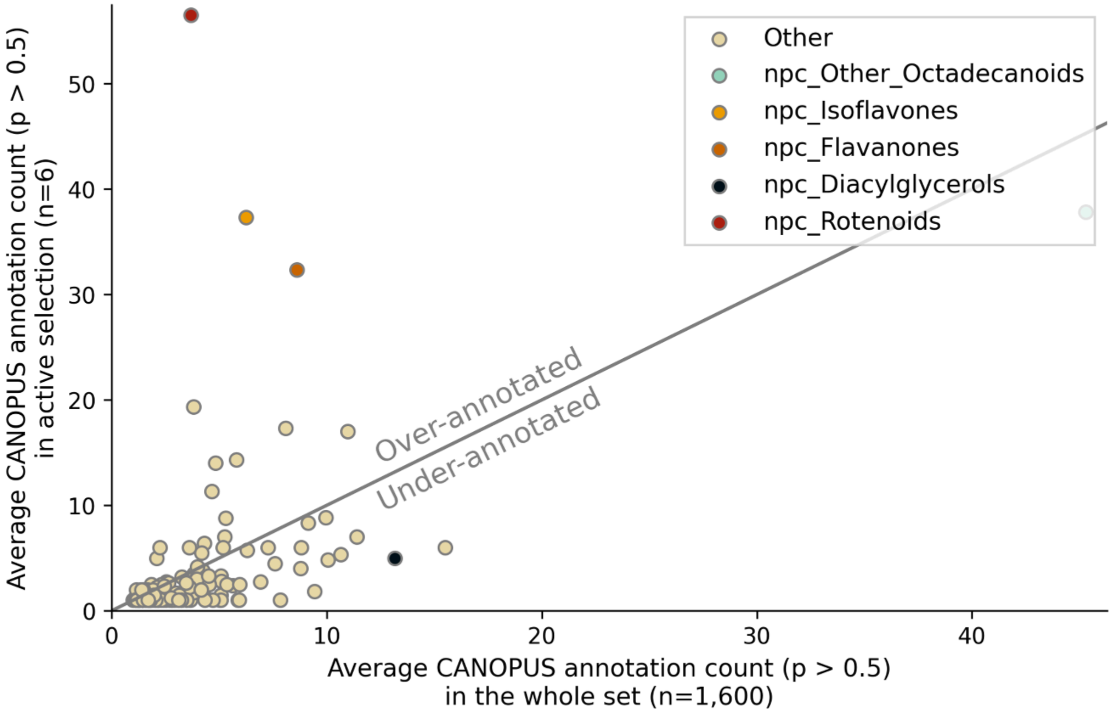

Figure S2: Scatter plot of the average occurrence in CANOPUS annotations (PI mode only, probability > 0.5) of each NPClassifier class in each sample of the datasets (n=1,600) versus the average occurrence in each sample of the selection (clustered *T. cruzi* hit samples, n=6). Only chemical classes present in the selection's CANOPUS annotations are plotted. Chemical classes above the gray line (rotenoids, flavanones, isoflavones) are overrepresented in the selection, while those under the line are underrepresented in the selection (diacylglycerols, other octadecanoids). Link to SPARQL query.

> SPARQL query: How many features by sample (1,600 plant extracts dataset) are annotated with confidence (p > 0.5) as rotenoids by CANOPUS ?

Returns: 207 samples with at least 1 feature annotated as rotenoid, and the number of features (PI and NI modes) annotated as rotenoids

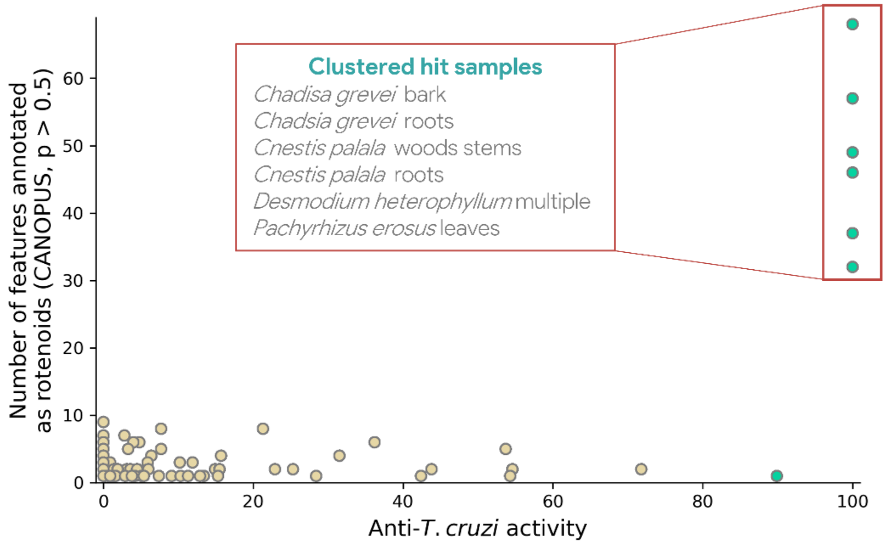

Figure S3: Scatter plot of the anti-T. cruzi activity versus the count of features annotated as rotenoids by CANOPUS, with a probability > 0.5. 207 samples with at least 1 feature annotated as a rotenoid are plotted. Hit samples on *T. cruzi* are colored in green. Link to SPARQL query.

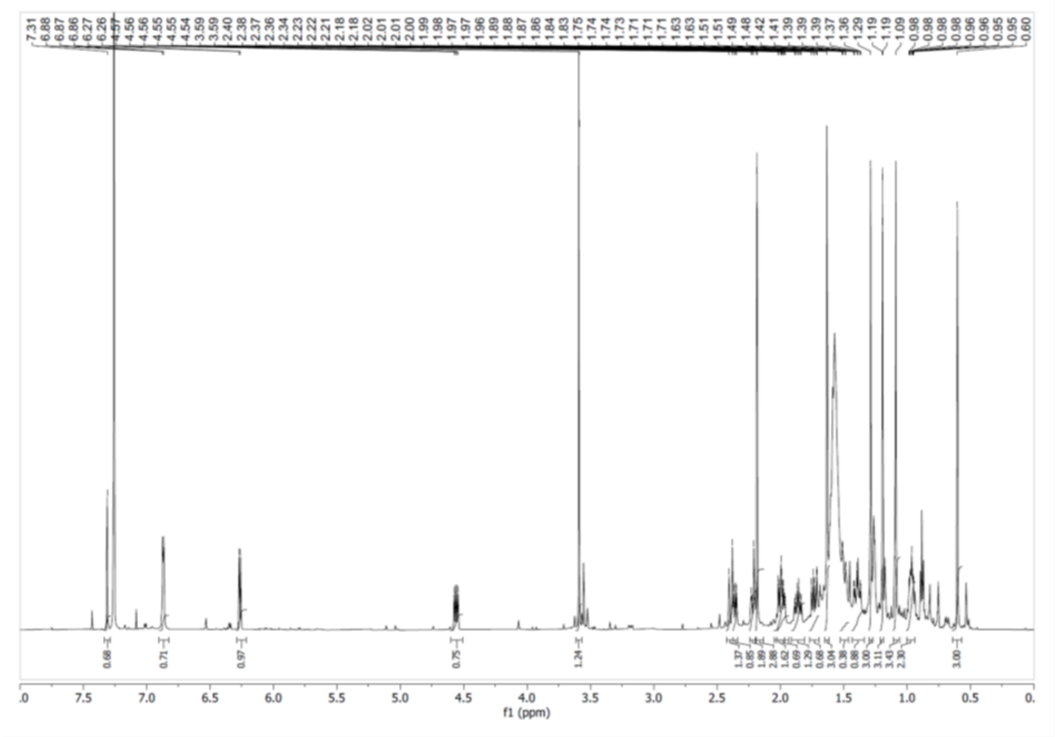

Figure S4: ^{1}H NMR spectra of **11-*β*-hydroxypristimerin** (3) in $CDCl_3$ at 600 MHz

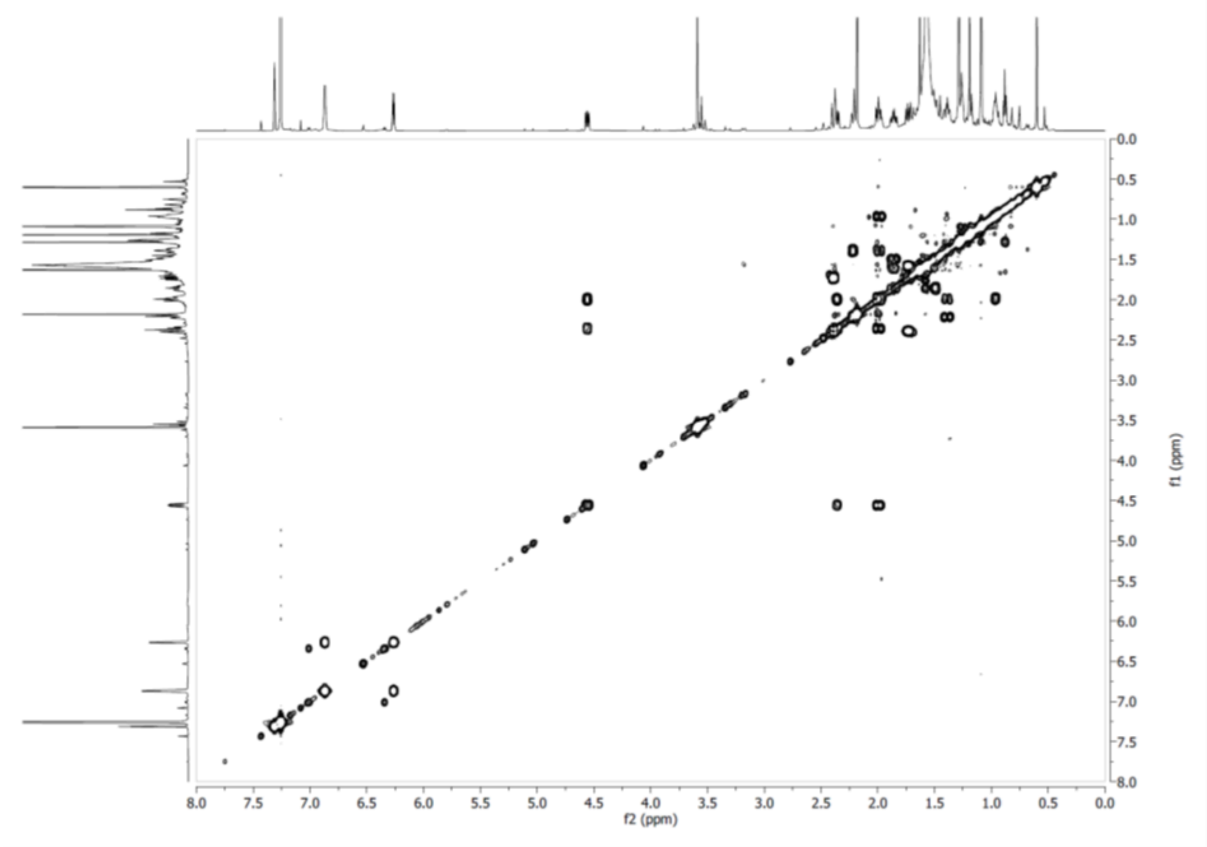

Figure S5: COSY NMR spectra of **11-*β*-hydroxypristimerin (3)** in $CDCl_3$ at 600 MHz.

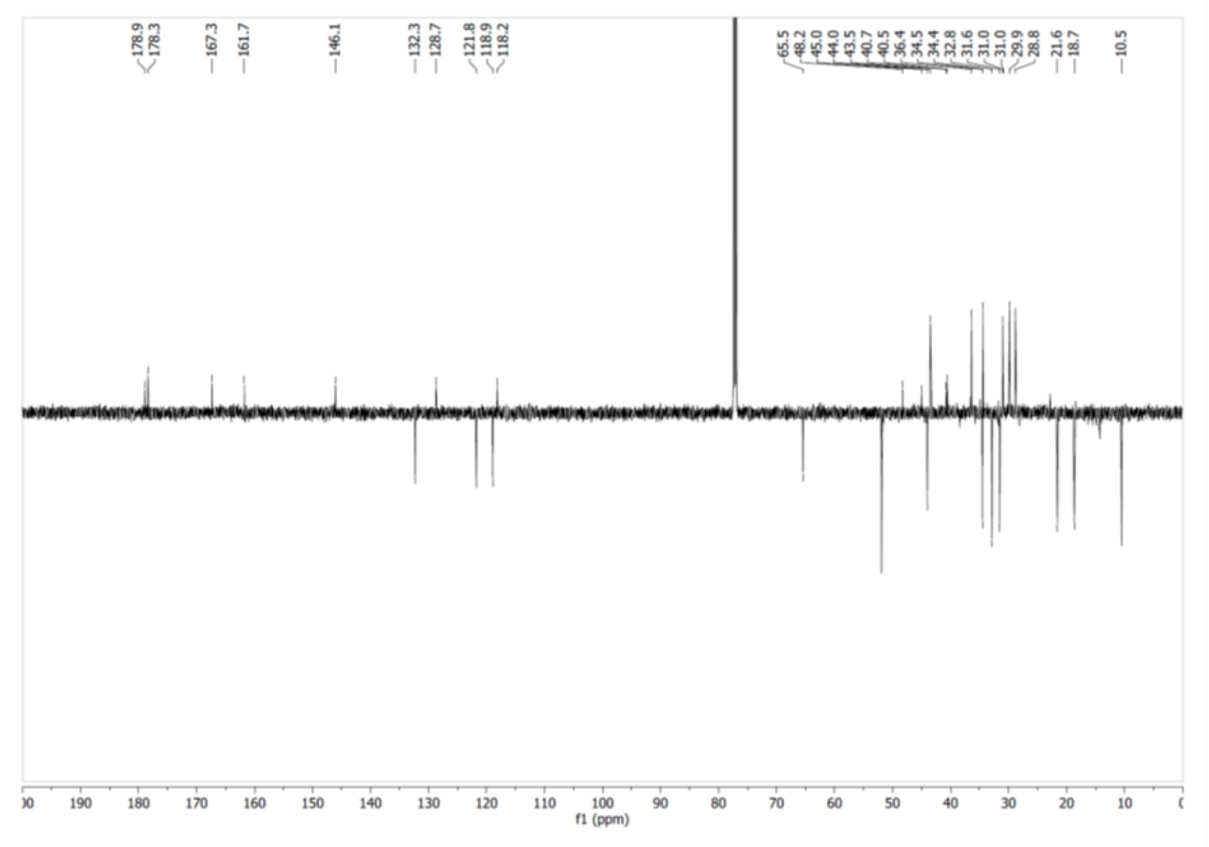

Figure S6: ^{13}C-DEPTQ NMR spectrum of **11-*β*-hydroxypristimerin (3)** in $CDCl_3$ at 151 MHz

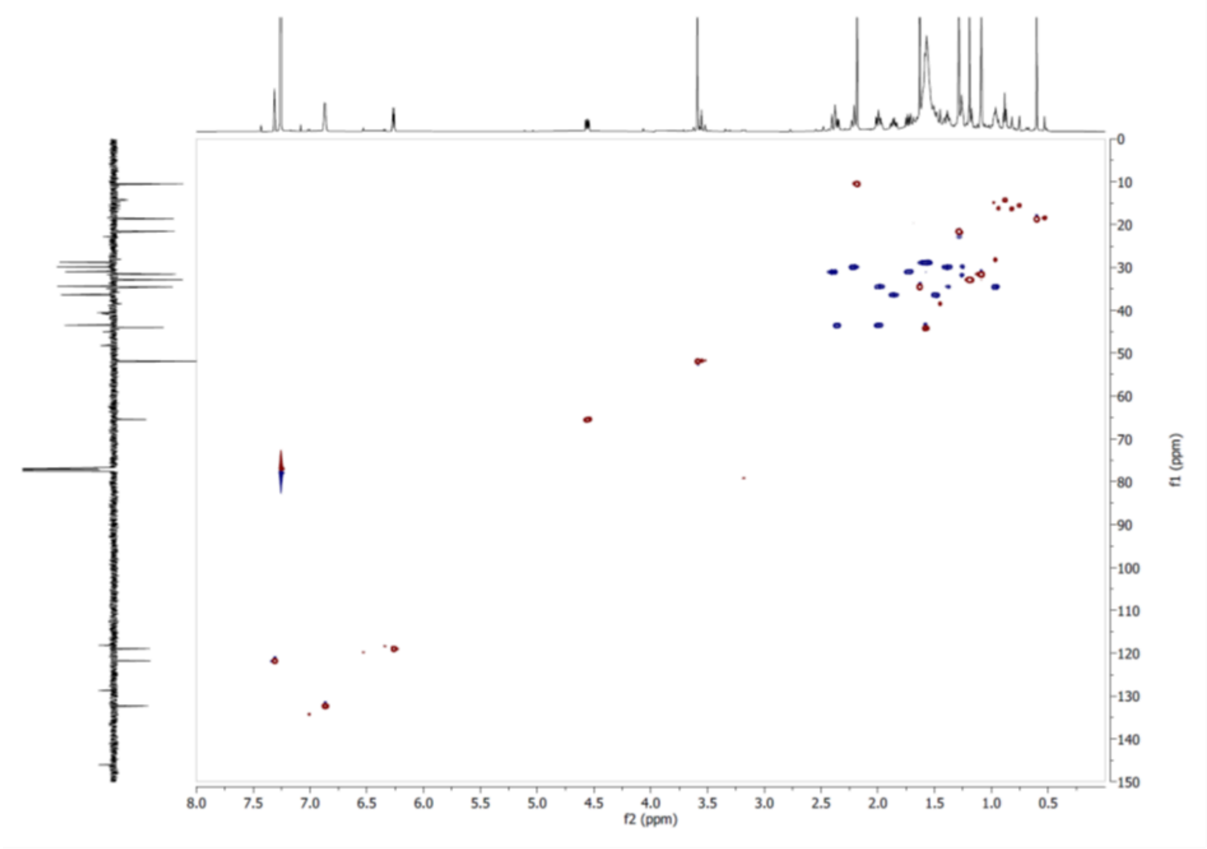

Figure S7: Edited-HSQC NMR spectrum of **11-*β*-hydroxypristimerin (3)** in $CDCl_3$ at 151 MHz

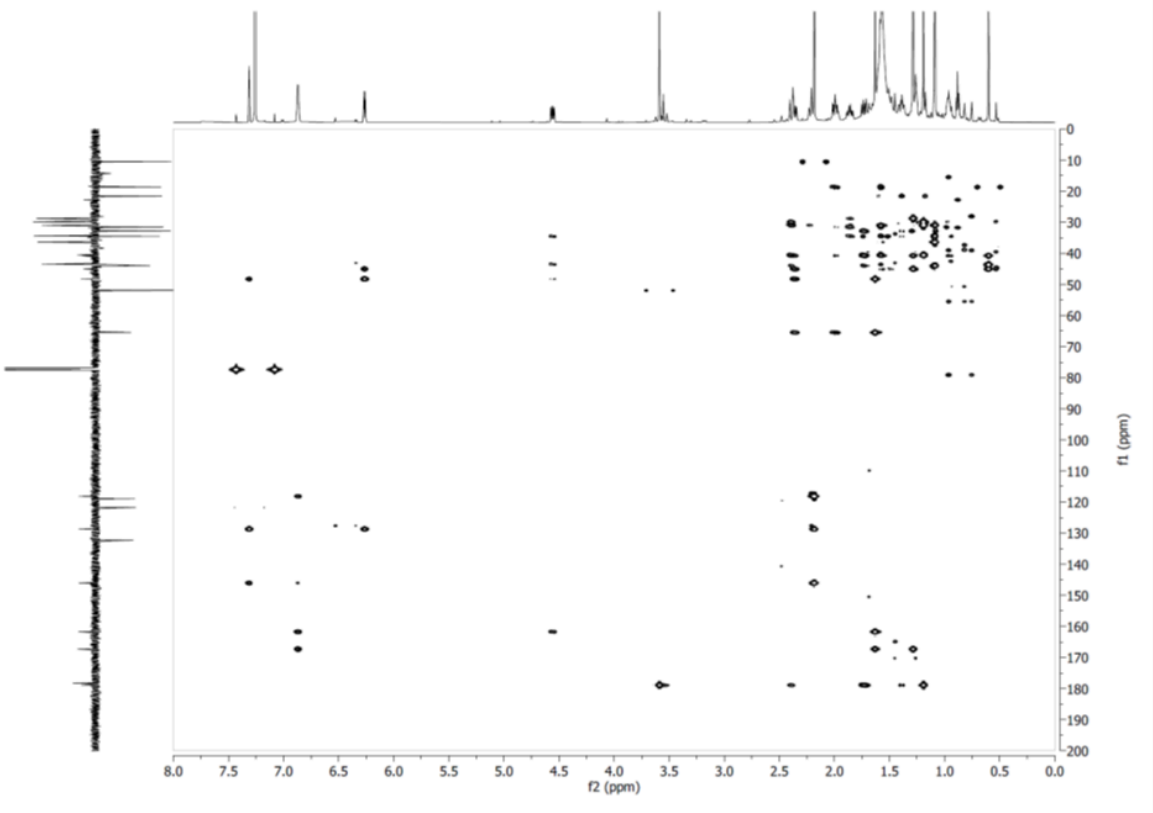

Figure S8: HMBC NMR spectrum of **11-*β*-hydroxypristimerin (3)** in $CDCl_3$ at 151 MHz

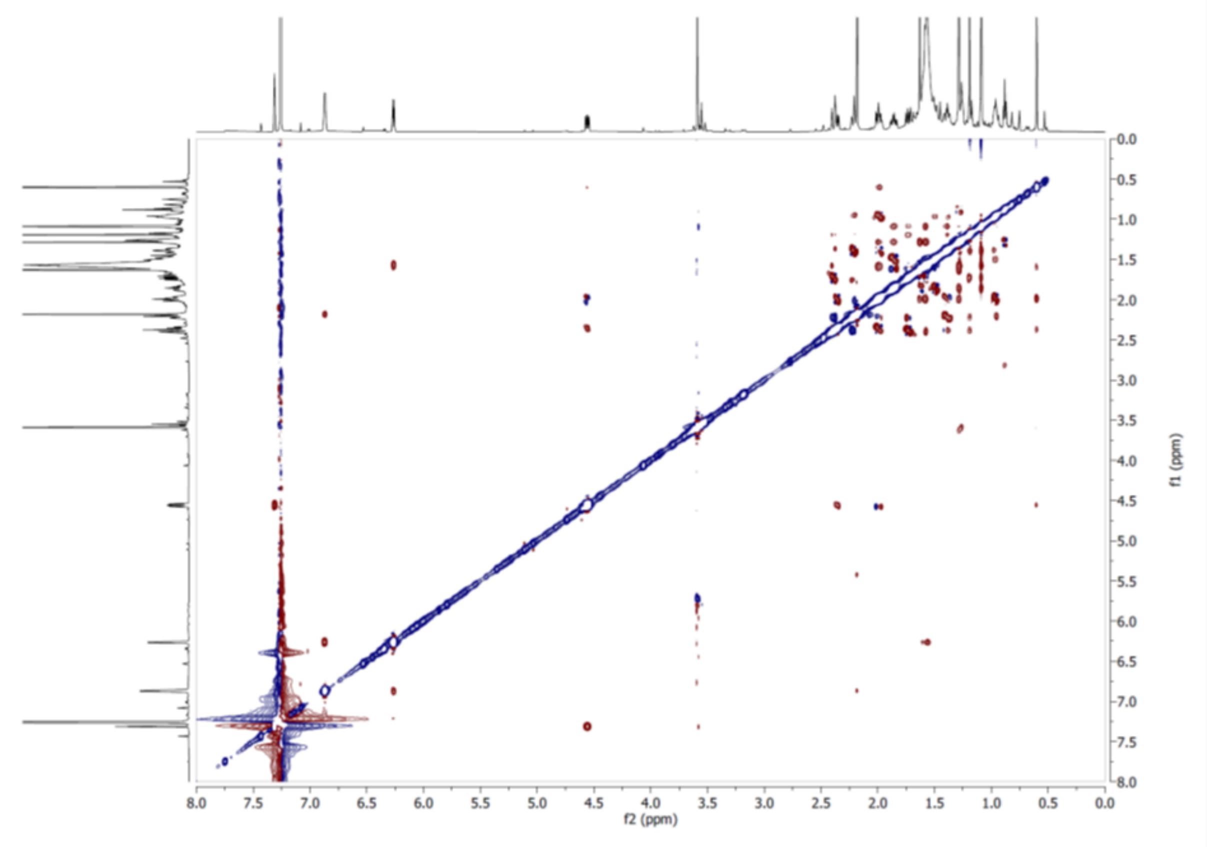

Figure S9: ROESY NMR spectrum of **11-*β*-hydroxypristimerin (3)** in $CDCl_3$ at 151 MHz

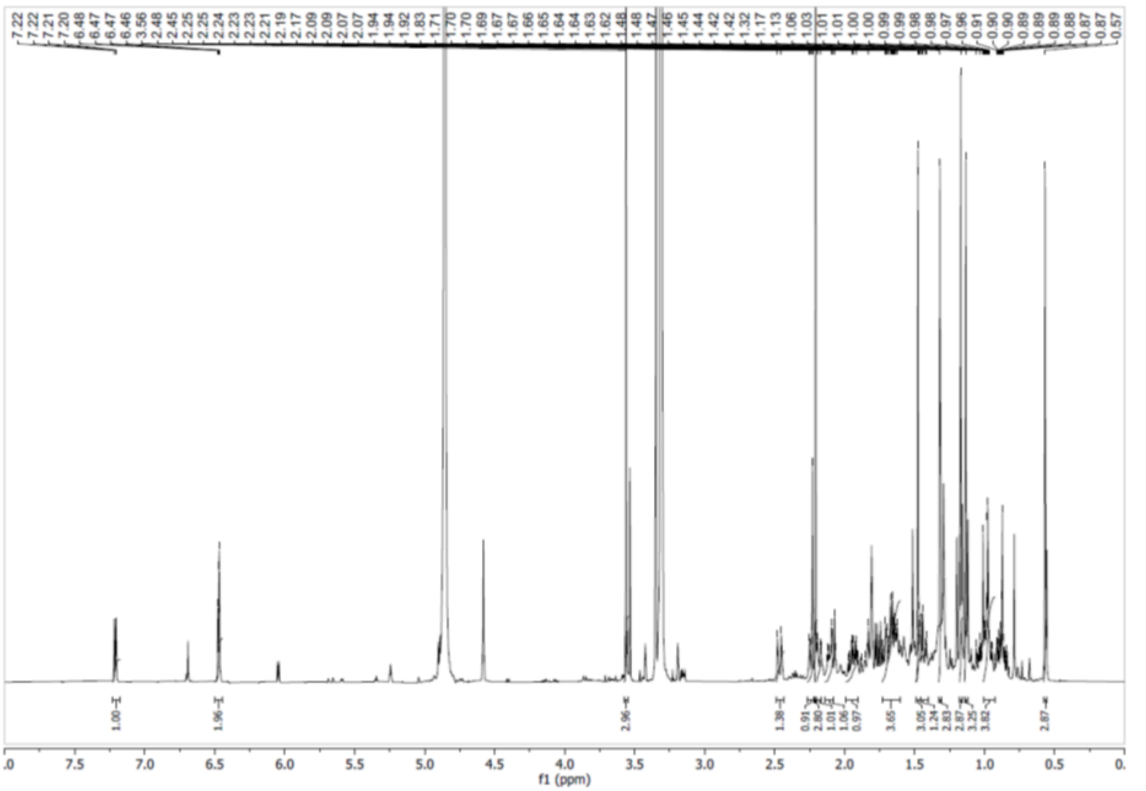

Figure S10: ^{1}H NMR spectra of **pristimerin (4)** in $CDCl_3$ at 600 MHz

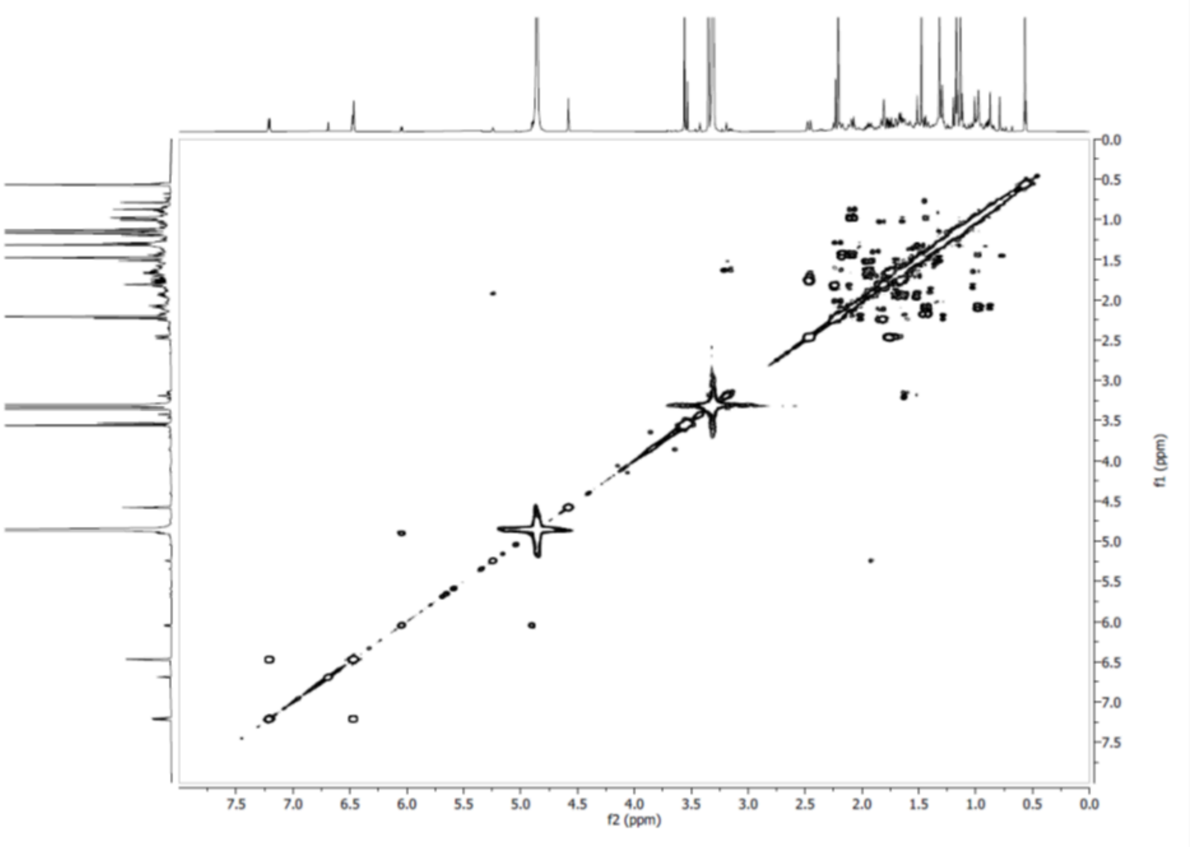

Figure S11: COSY NMR spectra of **pristimerin (4)** in $CDCl_3$ at 600 MHz.

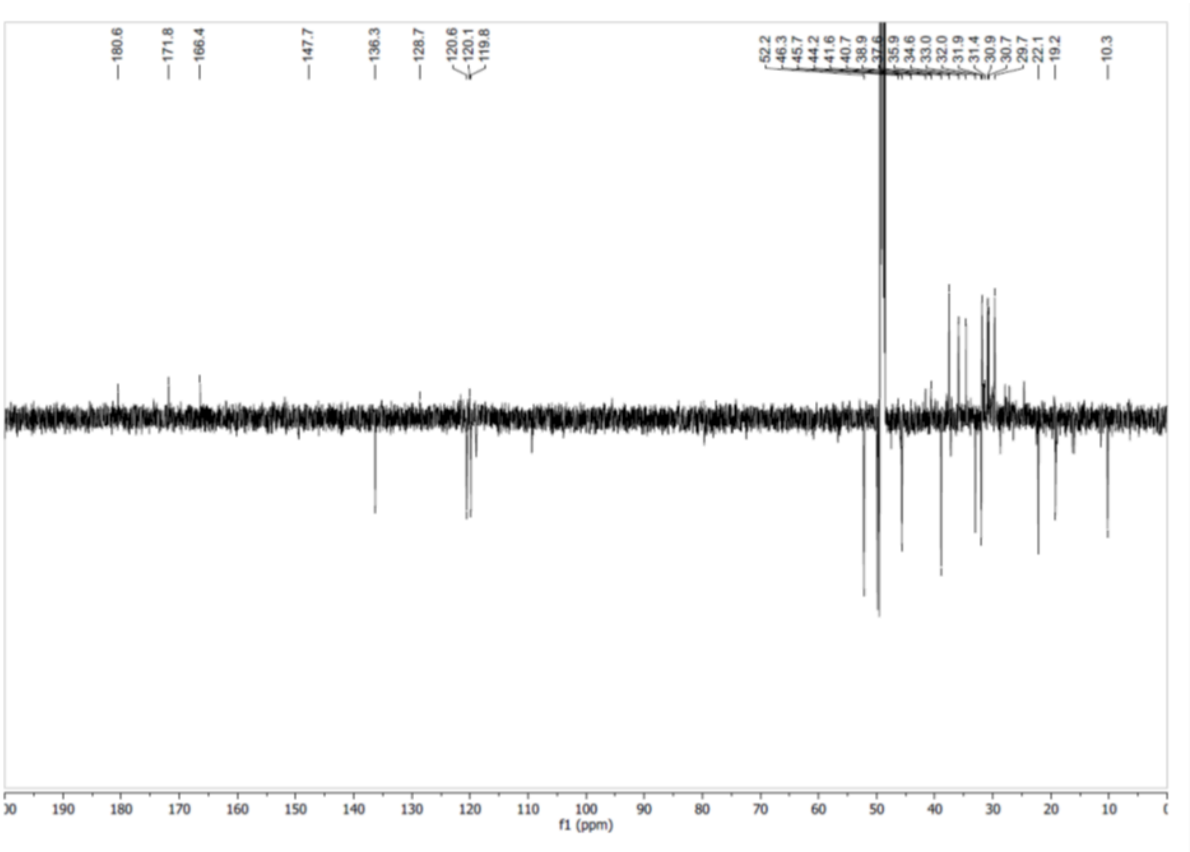

Figure S12: ^{13}C-DEPTQ NMR spectrum of **pristimerin (4)** in $CDCl_3$ at 151 MHz

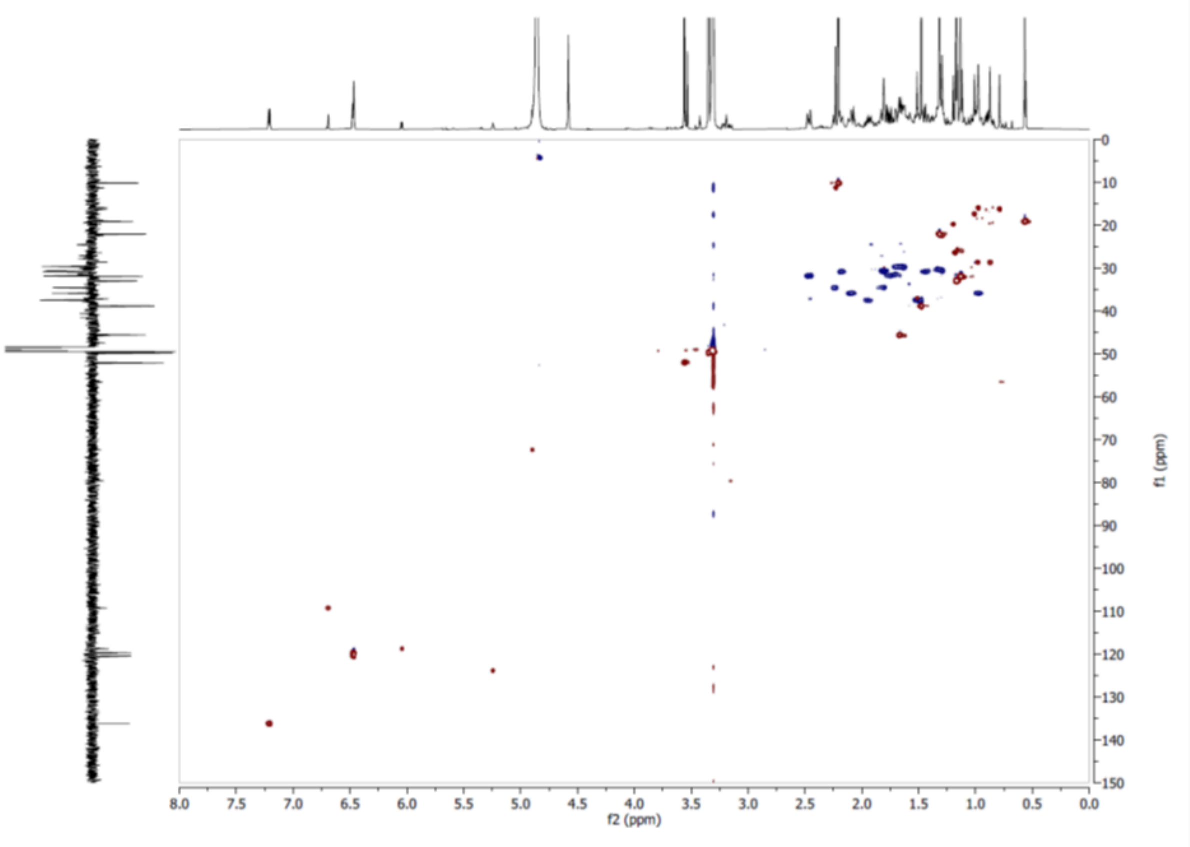

Figure S13: Edited-HSQC NMR spectrum of **pristimerin (4)** in $CDCl_3$ at 151 MHz

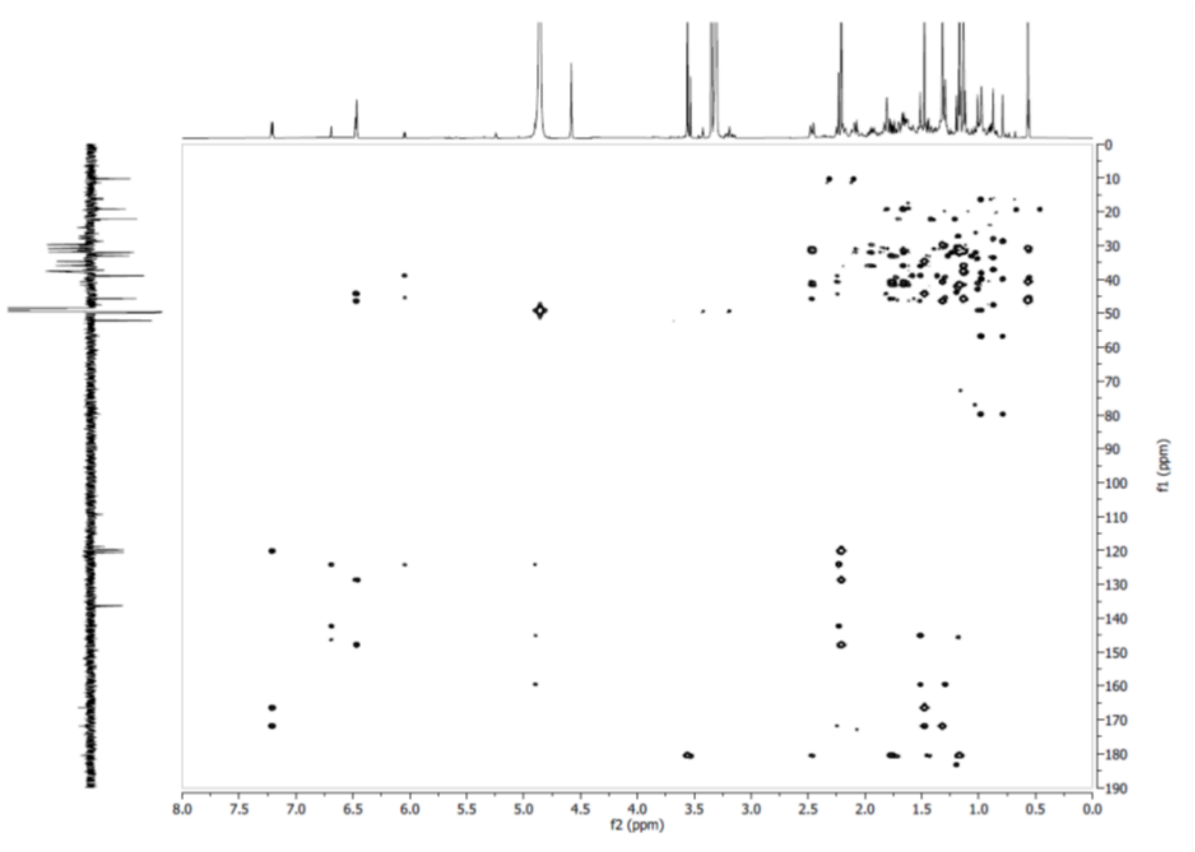

Figure S14: HMBC NMR spectrum of **pristimerin (4)** in $CDCl_3$ at 151 MHz

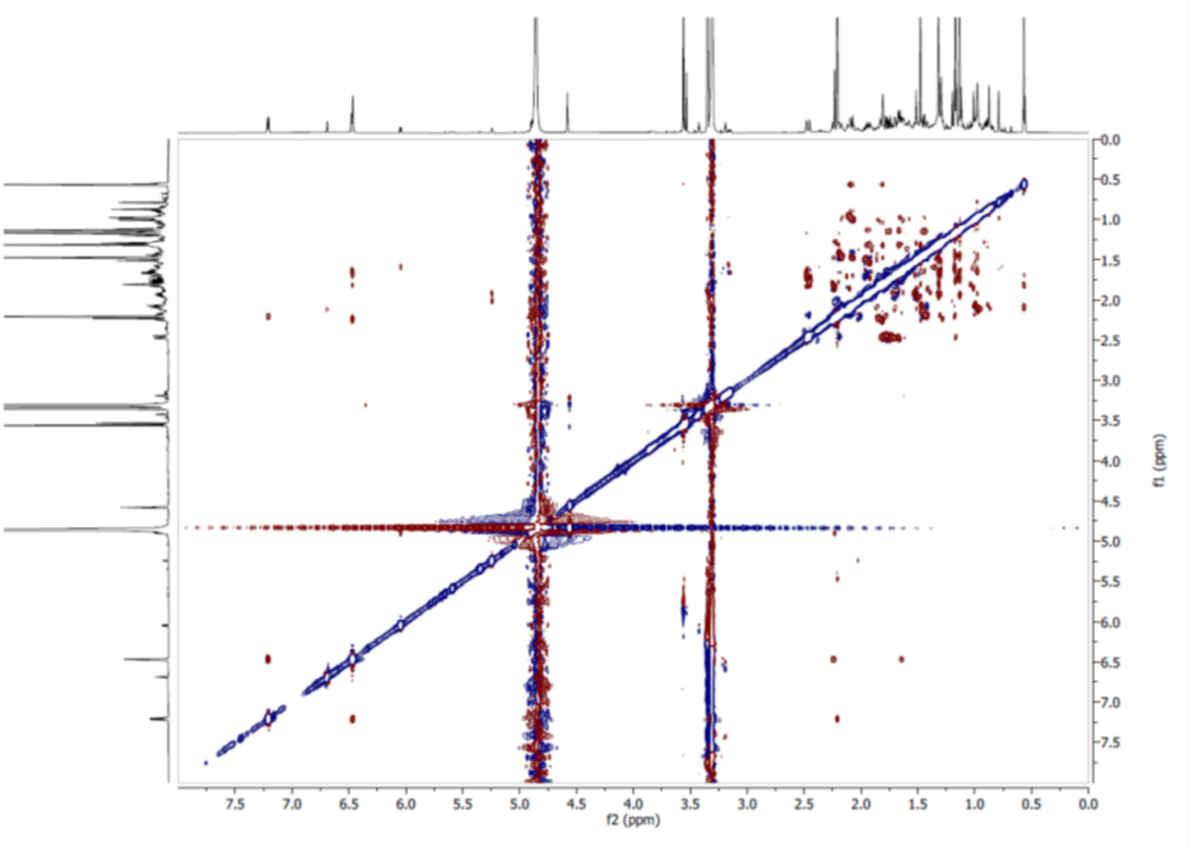

Figure S15: ROESY NMR spectrum of **pristimerin (4)** in $CDCl_3$ at 151 MHz

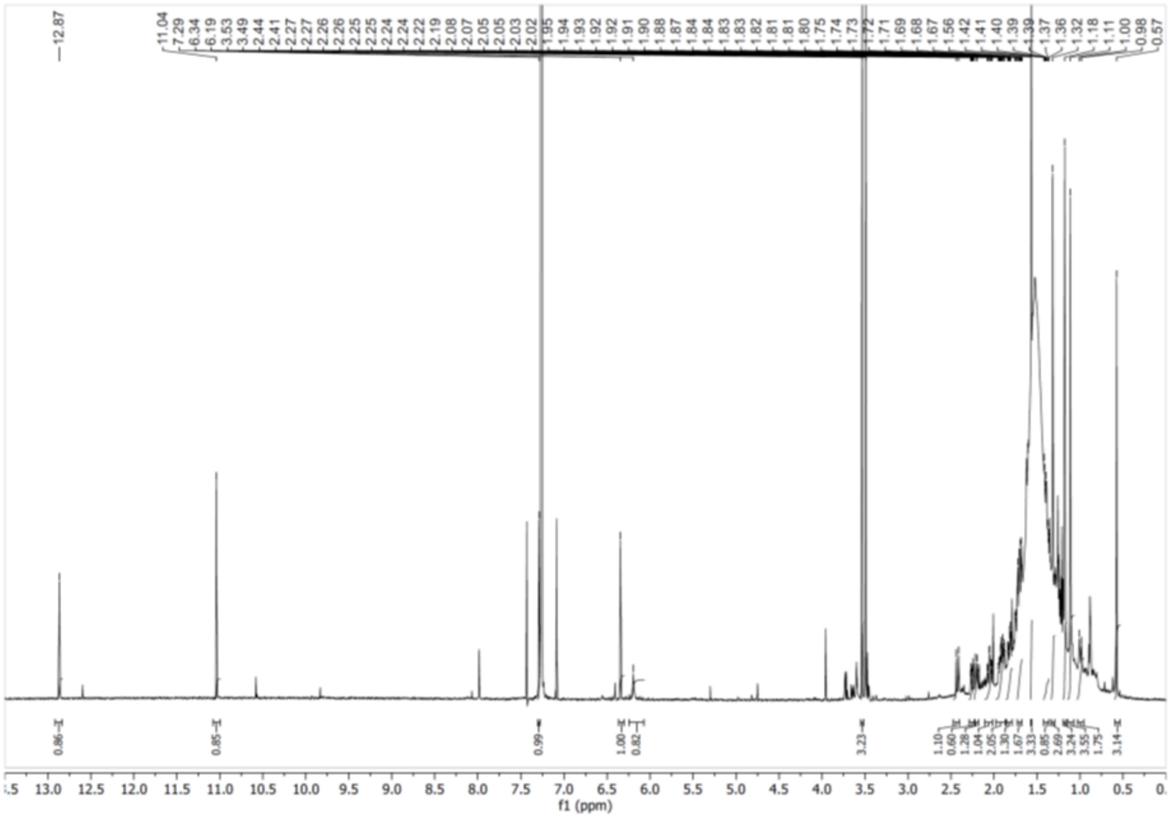

Figure S16: ^{1}H NMR spectra of **zeylasteral (5)** in $CDCl_3$ at 600 MHz

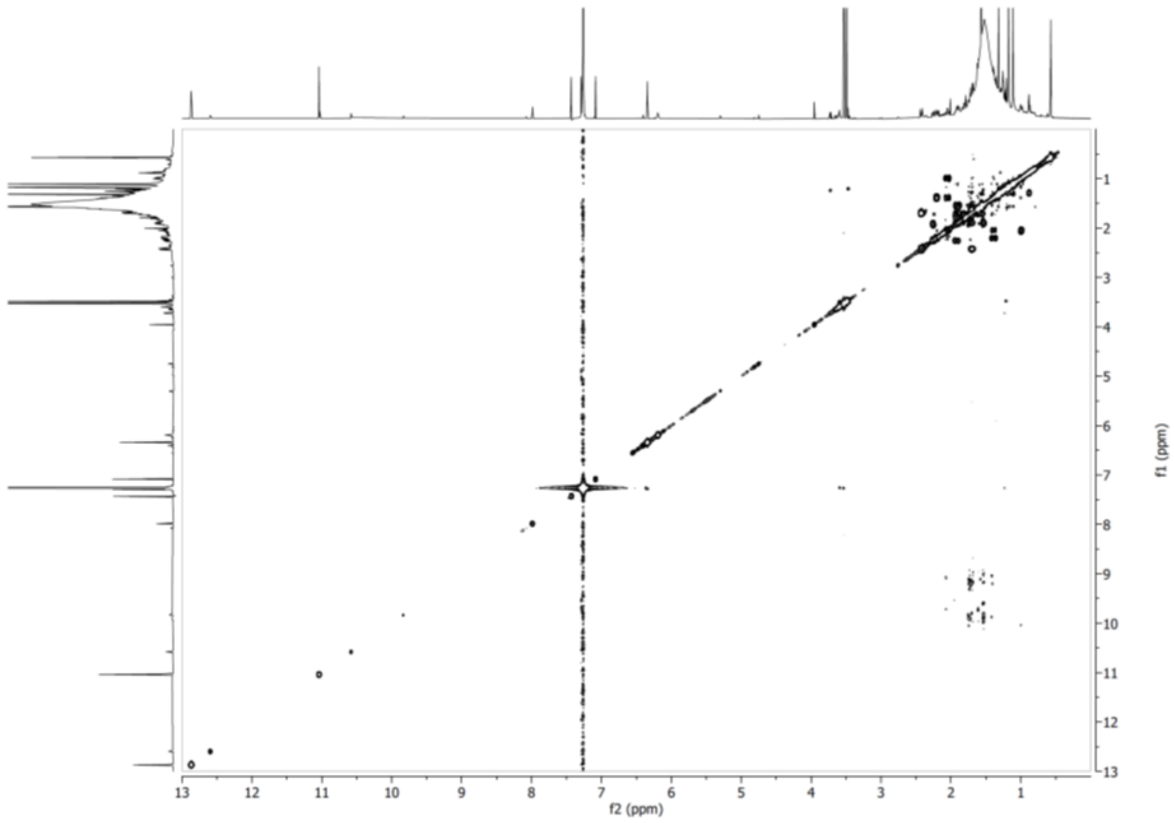

Figure S17: COSY NMR spectra of **zeylasteral (5)** in $CDCl_3$ at 600 MHz.

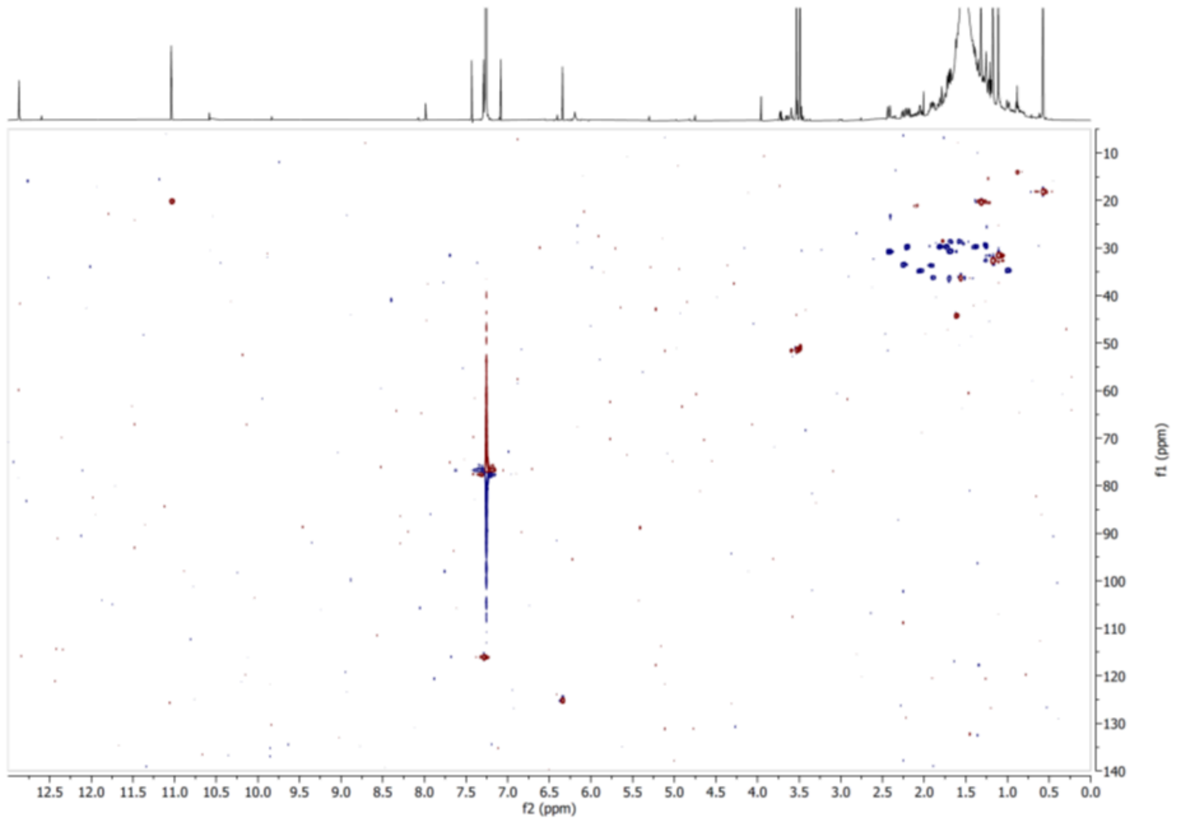

Figure S18: Edited-HSQC NMR spectrum of **zeylasteral (5)** in $CDCl_3$ at 151 MHz

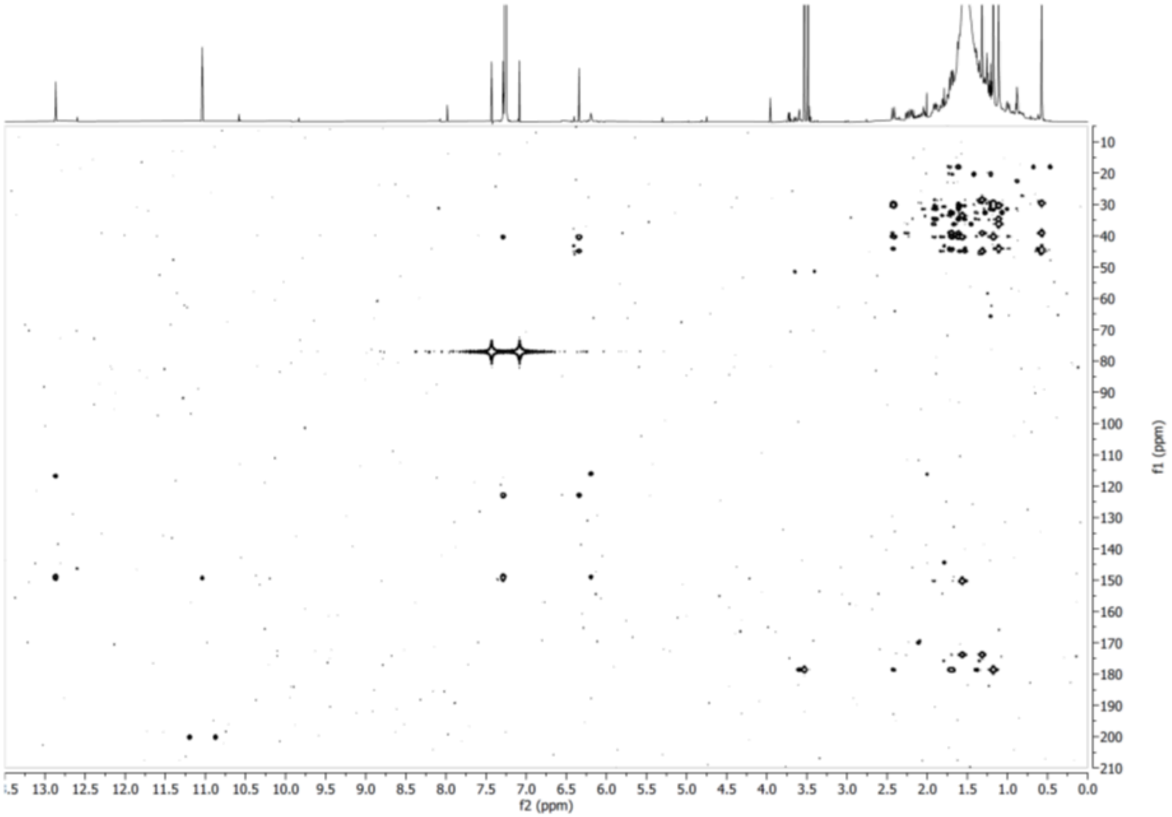

Figure S19: HMBC NMR spectrum of **zeylasteral (5)** in $CDCl_3$ at 151 MHz

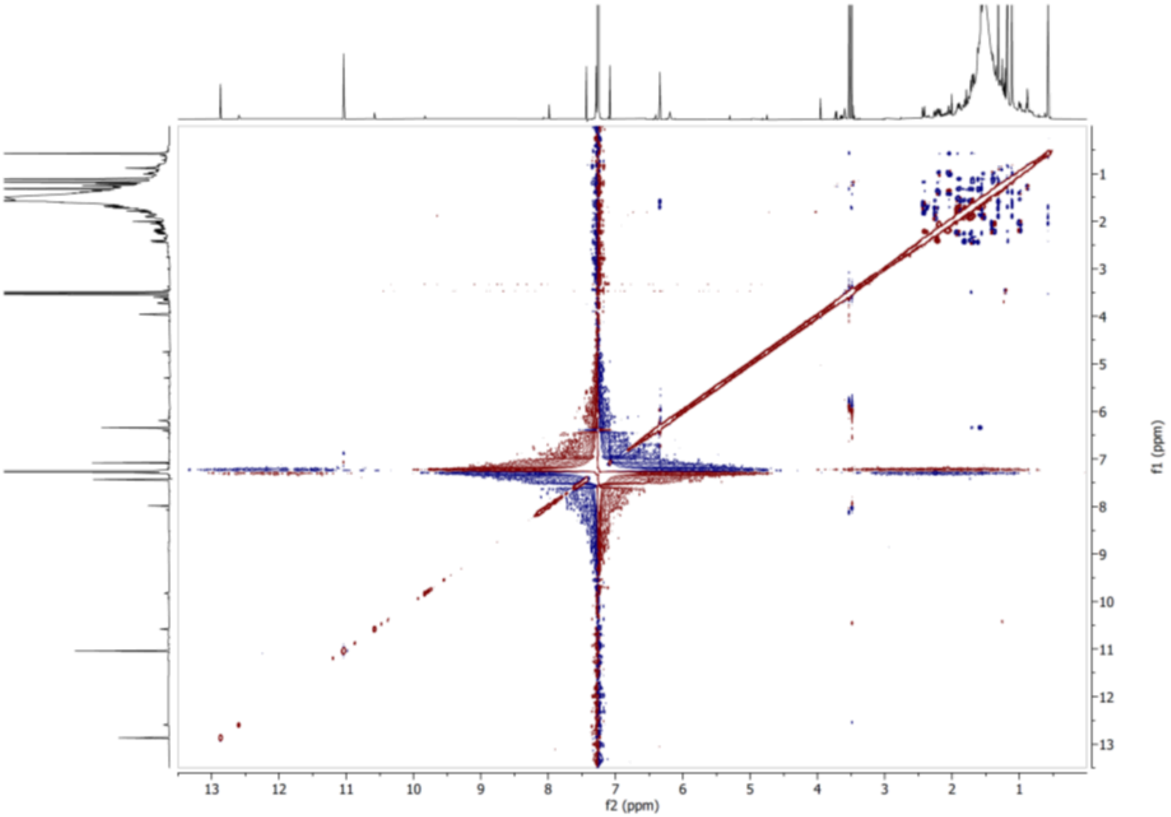

Figure S20: ROESY NMR spectrum of **zeylasteral (5)** in $CDCl_3$ at 151 MHz

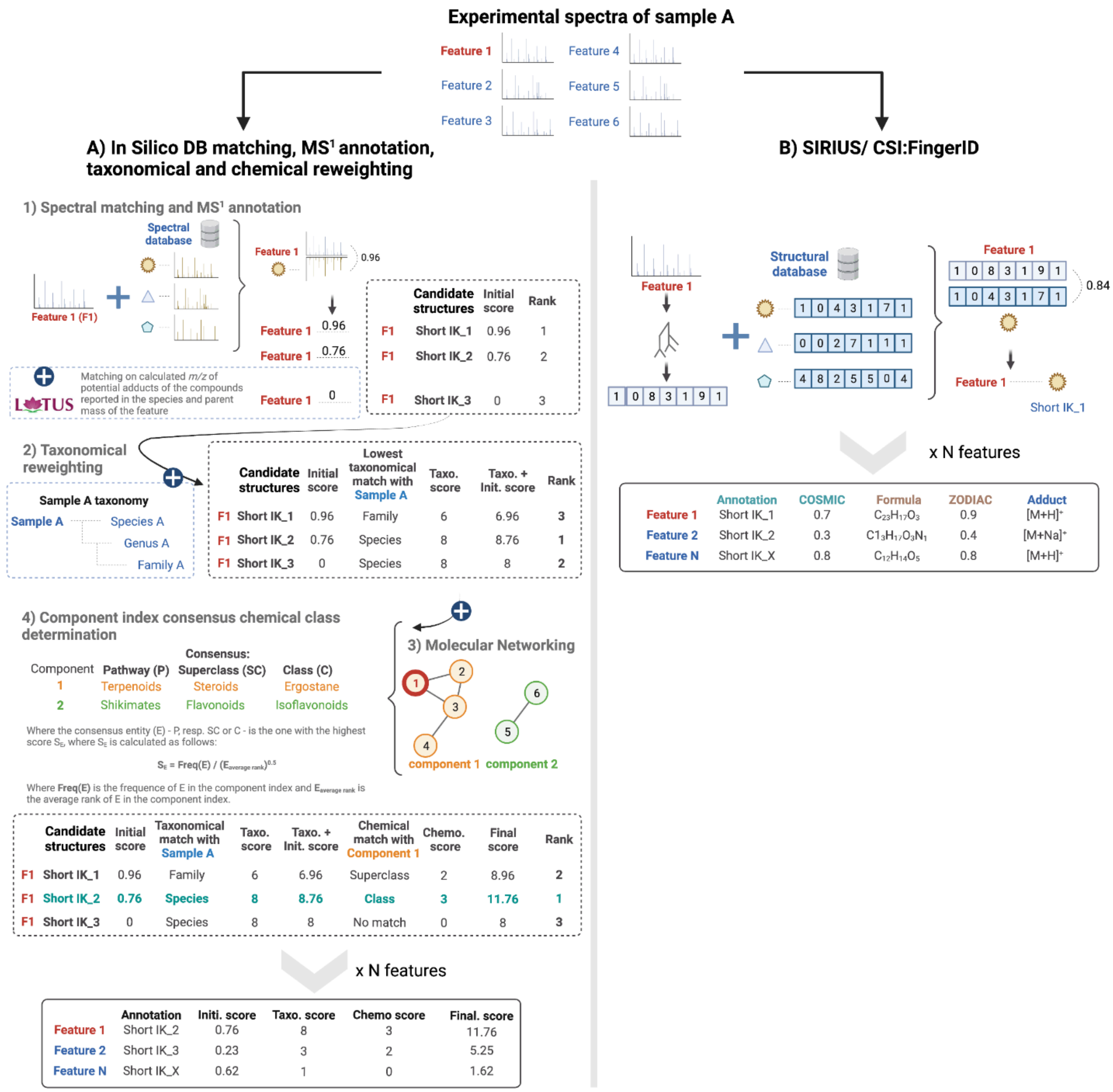

Figure S21: Schematic overview of the annotation workflow applied in the ENPKG framework. In **A)**, the *In Silico* DB matching, MS[1] annotation, taxonomical and chemical consistency reweighting. **B)** Sirius coupled to CSI:FingerID process. In **A**), the following process is applied for each feature: **1)** the feature's MS[2] spectrum is matched against an *in silico* generated spectral DB of NPs structures obtained from the LOTUS ressource (ISDB-LOTUS). This step returns a list of potential annotations as well as the spectral (or initial) score (cosine) between the candidate and the queried spectrum. Because the ISDB contains only [M+H]+ adducts, the feature's parent mass is also matched against all potential adducts' *m/z* of all the compounds reported in the same species than the one of the queried feature (MS[1] annotation). For these candidates, the initial score is set to 0. The second step (**2**) is the taxonomical reweighting: for each candidate annotation from step 1, the lowest common taxonomical level between the biological source(s) of the annotation and the one of the sample that contains the feature is retrieved. Based on this, a taxonomical score is given to the candidate annotation: if the candidate is reported in the same species as the considered feature, a score of 8 is given. The higher the match, the lower the score: 7 for the same genus, 6 for the same family, etc. The process's third step (3) is the FBMN

and the chemical reweighting (**4**). Based on the topology of the MN and the candidates' annotations of all the features, a consensus chemical class, superclass, and pathway are calculated for each component, or molecular family, of the FBMN. These consensus entities are then used to calculate the chemo-score: if the annotation shares the same chemical class as the consensus of its component, a chemo-score of 3 is attributed (2 for the superclass and 1 for the pathway). Finally, the three obtained scores (initial, taxonomical, and chemical) are summed to obtain the final score, and the candidate annotation with the highest final score is kept.
