# Supplementary material for: A Sample-Centric and Knowledge-Driven Computational Framework for Natural Products Drug Discovery
Source: ACS Cent Sci. 2024 Feb 20;10(3):494–510. doi: 10.1021/acscentsci.3c00800 (PMC10979503; doi:10.1021/acscentsci.3c00800)
Supplement: Supplementary file 2 — oc3c00800_si_003.pdf [file oc3c00800_si_003.pdf]

oc-2023-00800h.R1

Name: Peer Review Information for "A Sample-Centric and Knowledge-Driven Computational Framework for Natural Products Drug Discovery"

First Round of Reviewer Comments

Reviewer: 1

Comments to the Author

The manuscript entitled "A Sample-Centric and Knowledge-Driven Computational Framework for Natural Products Drug Discovery" by Gaudy et al., describes a new computational strategy for to parse metabolic data that is purported to reduce dependency on particular retention times (allowing for easier comparison across data sets) and peak intensity and instead stresses the use of metadata and semantic web technologies to increase the efficiency of natural product-based drug discovery.

Overall the manuscript is good. I do, however suggest some revision prior to publication.

1. The authors stress the broad applicability of their system to handle data sets from different sources, gathered on different instruments however, their example extract library is uniformly from ethyl acetate extracts from plants. Their newer comparison extracts are also ethyl acetate extracts from similar plants. It would be good to show example including her extraction techniques to confirm the broad applicability put forth by the authors.

2. The authors also state that data from different suites can be directly compared but have only on, very similar, data set which they use for comparison. The manuscript wold be strengthened by a comparison with publicly available MS2 data from GNPS which demonstrates this broad applicability.

3. The authors stress the increased importance of metadata in their method but do not address the significant difference in the quality of publicly-available metadata on various natural product collections. Some acknowledgement of the importance of properly annotated metadata (taxonomy, plant part etc.) would be helpful.

4. The authors show that their system does not rely on RT and peak intensity for their analysis. Some statement on the effect of varies MS settings (i.e. fragmented voltage differences) on the MS2 data and their ability to compare datasets would help the reader.

5. The authors show an examples of where collecting metabolic data on 1600 samples and using their analysis methodologies increased the efficiency of determining active structured from 6 bioactive samples. The relative efficiency of such large-scale data collection vs. the more direct analysis of only active fractions would be an informative area for the discussion section and would strengthen the manuscript.

6. On pg. 12, the authors discuss how the a feature sharing the highest number of peaks and losses became "feature #1" of an active extract of Melochia umbellata. Would such a prioritization strategy be indicative of the relative abundance of a feature in an extract (an alternative measure similar to peak intensity for relative quantification of a feature to associate with bioactivity?

7. The authors state that retention time is not important for their methodology yes in Table 1 Query #7 &8 are both dependent on retention time data. Please explain.

8. The authors on pg. 16 use the unit micrograms/mL for specific compounds and later use molar units for other compounds. Molar units should be used for all compounds for ease of comparison.

Overall the paper is interesting and potentially helpful to the natural products research community. I recommend publishing after revision.

Reviewer: 2

Comments to the Author

Review of oc-2023-000800h

This is an interesting and innovative paper describing the application of knowledge graph methods to questions in natural products. These approaches have not seen large-scale adoption by this community, and it will therefore be of broad interest to that readership. Use of knowledge graphs is an emerging strategy for large-scale data integration in other fields of biomedical science. This paper brings value by

not only demonstrating applications of the methodology for antiparasitic compound discovery, but also providing a computational framework to generate KGs using toolsets available to the NP community. Notwithstanding the benefits, I have several issues that should be addressed.

1) Positioning of selected tools. The paper uses a suite of annotation tools for NP discovery that offer one solution to problems of annotation and identification of untargeted MS datasets. However the text is written to imply that these are the only solutions to such problems, which is not correct. The early sections of the paper need to be expanded to place this workflow in context with the rest of the field. MZMine is not the only MS processing software package, NP Classifier is not the only chemical ontology etc.

2) Demonstration of utility of KG methodology. I found the two examples provided to be interesting, but I was not convinced that these results would have been that difficult to extract without KG approaches. "Which extracts are active against target X with low cytotoxicity against Y" could easily be accomplished by filtering in Excel. The second stage, where SIRIUS annotations are counted and examined (Fig 3B) is more complicated, but still not that difficult to do by collating and sorting results files for individual samples. I appreciate that this approach is scalable and flexible, but as written the examples do not present a step change in search ability or knowledge generation compared to existing analogue methods. The authors should consider additional information that could be derived from the graph. For example, does the general rotenone scaffold have precedent as an antiparasitic agent against other kinetoplastid parasites? Are any of those compounds present in the active extract set? Or other samples in the NP library?

3) Accessibility for end users. Most NP chemists are not computer science experts. Although the paper is accompanied by a GitHub repository that includes wrappers for running each of the required tools and instructions about data formatting etc. this is still a manual process that requires users to install and run each step in series. Adoption would be much higher if these steps were wrapped into a single package (e.g. using Gooey) that gave end users an easier interface. This would be particularly valuable if packaged as a stand-alone app for both Windows and Mac. This is clearly significant additional work, but not that complicated given that most individual steps are already single command line arguments. This addition would improve standardization and could dramatically increase community adoption.

Reviewer: 3

Comments to the Author

This paper by Gaudry et al describes their approach to processing and interpreting LC-MS data for natural product research. A key part of their workflow is the removal of the feature alignment step that is widely used for analysis of large datasets in order to allow each sample to be considered individually in

the data analysis instead of trying to compare entire feature lists from different datasets. The authors have proposed a brilliant way to utilize the amount of data that is publicly available and yet extremely underutilized due to the limitations that they point out. They even went so far as to prove how it can be used to identify new natural products and have performed purification and structure elucidation of several compounds, which is no small feat. It is difficult to find fault in this work and the only comment is that it will be highly impactful to the field only if they can address the main drawback to their approach, namely the high level of expertise needed to implement the proposed workflow and by extension the inaccessibility to the average researcher. The authors do seem to acknowledge this, and this reviewer hopes they continue to pursue the development of an accessible user interface.

1. Section 2.3.1: Are the computer specs necessary for such a big dataset workflow prohibitory? It seems like it would be difficult to get MZmine to process so much data and then work with a list of > 1 million features and 800,000 in silico annotations on a typical data analysis computer. How long did it take to process this data and is this type of analysis possible for most LC-MS users? Or did they run a single sample through the MZmine workflow at a time?

2. If retention time information is not used to align features can the authors comment on whether they think the mzmine processing and feature networking is necessary/superior to a classical molecular networking workflow where mzmine processing is not used and really only MS2 patterns are used to create networks? Or do they view them as similarly effective?

3. Again, the main critique is that it would take an experience untargeted metabolomics scientist an extensive amount of time to learn how to implement this framework. Do they have plans for a user platform or integration into large platforms like GNPS?

Author's Response to Peer Review Comments:

Dear editors,

First of all, we would like to thank all the reviewers for their attentive reading of our manuscript and their constructive comments. We have carefully considered all comments and suggestions and have made the necessary changes to the manuscript. The relevance of the comments prompted us to make changes that required a significant amount of work, hence the delays in our response (for example, the full integration into the KG of a public dataset of 337 Korean medicinal plants published by others than ourselves to illustrate ENPKG's capabilities in handling heterogeneous datasets; the implementation of a consolidated CLI command-line interface as well as a pilot web portal (shared with reviewers only)). Nevertheless, we believe that these comments and the revision process have benefited the manuscript. We hope that the revised version of the manuscript can be shared with the readers of ACS Central Science.

Please find our detailed response in the attached files.

With best regards,

Pierre-Marie Allard on behalf of the ENPKG authors

Journal: ACS Central Science
Manuscript ID: oc-2023-00800h
Original Submission Date: 30-Jun-2023
Title: "A Sample-Centric and Knowledge-Driven Computational Framework for Natural Products Drug Discovery"
Author(s): Gaudry, Arnaud; Pagni, Marco; Mehl, Florence; Moretti, Sébastien; Quirós-Guerrero, Luis; Rutz, Adriano; Kaiser, Marcel; Marcourt, Laurence; Queiroz, Emerson; Ioset, Jean-Robert; Grondin, Antonio; David, Bruno; Wolfender, Jean-Luc; Allard, Pierre-Marie Dear Dr. Allard:

Thank you for your submission to ACS Central Science. We have now received the reviews of your manuscript.

In short, we like it. However, in its current form, your manuscript is not yet suitable for publication in ACS Central Science. The reviewers have raised points that require significant consideration and major revision that may include additional experiments/data and discussion. However, with adequate revisions, your manuscript may become acceptable for publication.

Key to me seem:

[Reviewer 1]
-comparison with publicly available MS2 data from GNPS which demonstrates this broad applicability.

[Reviewer 2]
-early sections of the paper need to be expanded to place this workflow in context with the rest of the field

[Reviewer 2 and 3]
-wrapped into a single software package that gives end users an easier interface. This seems quite key to us, if this work is to act as a true community resource, as we hope.

We would like to receive your revision no later than 04-Oct-2023. The revision should address the reviewers' comments and include a point-by-point response. Your manuscript may be subject to further peer review and likely sent back to one or more of the original referees.

Let us know if you need more time.

In addition to a clean copy of the revised manuscript, please also submit a tracked version of the original submission that shows the actual changes (deletions and additions) made to the manuscript. You may highlight, color font, or underline the changes.

ACS Central Science offers authors an opportunity to participate in transparent peer review. Transparent peer review allows the reader to see the exchange between authors and reviewers. Transparent peer review allows the reader to see the exchange between authors and reviewers. If an author chooses to participate in transparent peer review, the anonymous reviewers' comments and author's response to the reviewers will be published as supporting information if the manuscript is accepted for publication. More information about transparent peer review can be found here https://urldefense.com/v3/__https://pubs.acs.org/page/peer_reviews/transparent_peer_review.html__;!!Dc8iu7o!00V_fCwx0Dsxpw-hNBr0MKP5ZKdni6ffZmTvGJDbdMBY4G6YMHvss1WSBXOf2UfNGjXS2GbCRXfH7zeyMg28yCjPDflVnvBlzIzj$ or at a recently published editorial https://urldefense.com/v3/__https://pubs.acs.org/doi/10.1021/acscentsci.1c01238__;!!Dc8iu7o!00V_fCwx0Dsxpw-hNBr0MKP5ZKdni6ffZmTvGJDbdMBY4G6YMHvss1WSBXOf2UfNGjXS2GbCRXfH7zeyMg28yCjPDflVnv2c20Rl$.

During submission, you were given a choice to participate in transparent peer review. You responded as follows:

Yes, I will participate in transparent peer review.

If you opted to participate in transparent peer review, you can change your mind at any revision stage. If you have questions about transparent peer review not answered in our FAQs, please contact ACS Publications Support at support@services.acs.org or contact the editorial office.

Prior to submitting your revision, please also be sure to address the formatting issues listed above the reviewer comments. Further information regarding press, hero images, etc. is included in the attached 'Author Checklist' document. Please note that certain non-scientific needs are required prior to acceptance.

Funding Sources: Authors are required to report ALL funding sources and grant/award numbers relevant to this manuscript. Enter all sources of funding for ALL authors relevant to this manuscript in BOTH the Open Funder Registry tool in ACS Paragon Plus and in the manuscript to meet this requirement. See https://urldefense.com/v3/__http://pubs.acs.org/page/4authors/funder_options.html__;!!Dc8iu7o!00V_fCwx0Dsxpw-hNBr0MKP5ZKdni6ffZmTvGJDbdMBY4G6YMHvss1WSBXOf2UfNGjXS2GbCRXfH7zeyMg28yCjPDflVngi4XVUo$ for complete instructions.

ORCID: Authors submitting manuscript revisions are required to provide their own validated ORCID iDs before completing the submission, if an ORCID iD is not already associated with their ACS Paragon Plus user profiles. This iD may be provided during original manuscript

submission or when submitting the manuscript revision. You can provide only your own ORCID iD, a unique researcher identifier. If your ORCID iD is not already validated and associated with your ACS Paragon Plus user profile, you may do so by following the ORCID-related links in the Email/Name section of your ACS Paragon Plus account. All authors are encouraged to register for and associate their own ORCID iDs with their ACS Paragon Plus profiles. The ORCID iD will be displayed in the published article for any author on a manuscript who has a validated ORCID iD associated with ACS Paragon Plus when the manuscript is accepted. Learn more at https://urldefense.com/v3/__http://www.orcid.org__;!!Dc8iu7o!00V_fCwx0Dsxpw-hNBr0MKP5ZKdni6ffZmTvGJDbdMBY4G6YMHvss1WSBXOf2UfNGjXS2GbCRXfH7zeyMg28yCjPDfIVng9p7ATd$.

The guidelines for submitting (1) the revised manuscript, (2) responses to reviewer concerns, and (3) the annotated manuscript are provided below.

(1) To submit the revised version, log into ACS Paragon Plus with your ACS ID at https://urldefense.com/v3/__http://acsparagonplus.acs.org/__;!!Dc8iu7o!00V_fCwx0Dsxpw-hNBr0MKP5ZKdni6ffZmTvGJDbdMBY4G6YMHvss1WSBXOf2UfNGjXS2GbCRXfH7zeyMg28yCjPDfIVntQfT4k8$ and select "My Authoring Activity." There you will find your manuscript title listed under "Revisions Requested by Editorial Office." With the exception of your main text file, all of your original files will be available to you for review or replacement during the revision process. If you need to replace a file, please be sure to remove the original before uploading a new one. Please note that you must upload a new, revised manuscript file.

(2) The ACS Paragon Plus system also allows you to respond to the comments made by the reviewer(s) either in the text box provided or by attaching a file containing your detailed responses to all of the points raised by the reviewers.

(3) In addition to uploading your revised manuscript file, please also upload an annotated copy of the manuscript that tracks deletions and additions for the benefit of the reviewers and editor. This marked-up manuscript should be uploaded electronically in the File Upload section as "Supporting Information for Review Only".

ACS Central Science is a diamond open access journal and publishes exclusively using a Creative Commons Attribution license (CC-BY) license. If your article is accepted in ACS Central Science, your article will be published under a CC-BY license at no charge.

ACS Publications uses CrossCheck's iThenticate software to detect instances of similarity in submitted manuscripts. In publishing only original research, ACS is committed to deterring plagiarism, including self-plagiarism. Your manuscript may be screened for similarity to published material.

We look forward to receiving your revised manuscript.

Best wishes,

Ben Davis
Senior Editor
ACS Central Science
Phone: + 44 (0)1865 275652
Fax: (202) 513-8930
Email: davis-office@centralscience.acs.org

--

Formatting Needs:

PULL QUOTE (OUTLOOKS + IN FOCUS): We encourage you to select 3 - 4 quotes from your submission that you would like highlighted in your paper. The quotes should be one sentence-long, unique to the submission and not from previously cited work. Please list your quotes at the end of the manuscript file.

Done

SIGNIFICANCE STATEMENT (IN FOCUS): Please edit your Abstract to be reflective of a Significance Statement. The Significance Statement should be a brief 60 word explanation of how your work will be a good resource for the community. It should be labeled as this instead of Abstract as well.

Done

TOC: Please move to the last page of the manuscript, beneath the References, and label "TOC Graphic"

Done

SI PARAGRAPH: If the manuscript is accompanied by any supporting information for publication, a brief description of the supplementary material is required in the manuscript. The appropriate format is: Supporting Information. Brief statement in non-sentence format listing the contents of the material supplied as Supporting Information.

Done

SI IN MS: Please separate your Supporting Information from the MS, and upload a clean copy as "Supporting Information for Publication." Please make sure the SI file includes a Title Page with all the identical information found on the MS Title Page.

Done

In addition, the supporting information pages must be numbered consecutively, starting with page S1. Done

----

Reviewer(s)' Comments to Author:

Reviewer: 1

Recommendation: Reconsider after major revisions noted.

Comments:

The manuscript entitled "A Sample-Centric and Knowledge-Driven Computational Framework for Natural Products Drug Discovery" by Gaudy et al., describes a new computational strategy for to parse metabolic data that is purported to reduce dependency on particular retention times (allowing for easier comparison across data sets) and peak intensity and instead stresses the use of metadata and semantic web technologies to increase the efficiency of natural product-based drug discovery.

Overall the manuscript is good. I do, however suggest some revision prior to publication.

1. The authors stress the broad applicability of their system to handle data sets from different sources, gathered on different instruments however, their example extract library is uniformly from ethyl acetate extracts from plants. Their newer comparison extracts are also ethyl acetate extracts from similar plants. It would be good to show example including her extraction techniques to confirm the broad applicability put forth by the authors.

The designed approach can indeed align samples in a retention-time agnostic manner, hence allowing the comparison of datasets acquired in different chromatographic conditions but also extracts obtained from different experimental conditions. The main extract collection studied in the frame of this paper has indeed been constituted using EtOAc as an extraction solvent.

However, the additional extracts dataset from *Waltheria indica* (https://doi.org/doi:10.25345/C5129X), which were used to both illustrate the incremental sample addition process and quickly highlight the presence of anti-trypanosomatid quinolones from *Melochia umbellata*, were acquired under different chromatographic and extraction conditions. They are not EtOAc extracts but **dichloromethane** extracts, hence illustrating the versatility of the approach, also regarding several extraction methods. We thank the reviewer for this comment and have more clearly underlined the difference in the extraction method in the manuscript.

Original: "To illustrate the capacities of the ENPKG approach for incremental sample addition, we also integrated data from three *Waltheria indica* (Q7966688) samples acquired in the context of a previous project at our lab, including a sample profiled in 2014 on a different analytical platform [14]."

Edited: “To illustrate the capacities of the ENPKG approach for incremental sample addition, we also integrated data from three *Waltheria indica* (Q7966688) samples acquired in the context of a previous project at our lab, including samples profiled in 2014 on a different analytical platform, using different extraction protocols [14].”

Furthermore, we have integrated an external dataset (MSV000086161, which was entirely extracted using MeOH see https://www.nature.com/articles/s41597-022-01662-2), illustrating the feasibility of comparing datasets extracted under different experimental conditions. Please refer to the answer to Question 2 below for details.

2. The authors also state that data from different suites can be directly compared but have only on, very similar, data set which they use for comparison. The manuscript wold be strengthened by a comparison with publicly available MS2 data from GNPS which demonstrates this broad applicability.

As underlined by the reviewer, the possibility of comparing multiple datasets acquired under different experimental conditions and the connection of the knowledge graph contents with external public data sources constitute one of the most substantial advantages of the proposed approach.

However, our approach makes it possible to connect the ENPKG formatted datasets directly to public spectral repositories. We thank the reviewer for his comment. Following this suggestion, we have greatly improved the interconnection with the globality of the MS/MS spectral repositories available in the GNPS ecosystem. For this, we have added 4 additional links to *each of the spectrum* of the ENPKG. These four additional links allow direct spectral matching using the Fasst Search interface. The *fast_search_gnpsdata_index* links allow the realization of a spectral similarity match of the spectrum against all of the GNPS index, hence allowing the identification of related, but not necessarily annotated spectra, and thus quickly identifying relevant datasets and eventually fostering collaboration across researchers. The *fast_search_gnpslibrary* links are limited to the GNPS spectral libraries and can thus be used to identify experimental spectral matches. Both search modes are also provided in “analog” mode, allowing us to annotate structural analogs.

Enclosed are examples of spectral search links for Feature 4 of an extract of *Datura stramonium.*

- **enpkg:fast_search_gnpsdata_index_no_analog**
  https://fasst.gnps2.org/fastsearch/?usi1=mzspec:MSV000087728:VGF151_E05_features_ms2_pos.mgf:scan:4
- **enpkg:fast_search_gnpsdata_index_analog**
  https://fasst.gnps2.org/fastsearch/?usi1=mzspec:MSV000087728:VGF151_E05_features_ms2_pos.mgf:scan:4&analog_select=Yes

- **enpkg:fast_search_gnpslibrary_no_analog** https://fasst.gnps2.org/fastsearch/?usi1=mzspec:MSV000087728:VGF151_E05_features_ms2_pos.mgf:scan:4&library_select=gnpslibrary&analog_select=No
- **enpkg:fast_search_gnpslibrary_analog** https://fasst.gnps2.org/fastsearch/?usi1=mzspec:MSV000087728:VGF151_E05_features_ms2_pos.mgf:scan:4&library_select=gnpslibrary&analog_select=Yes

Here, for example, the enpkg:fast_search_gnpslibrary_no_analog links to a direct spectral search of this feature in the GNPS libraries, pointing in this case to an excellent spectral match (cosine 1) with scopolamine, a known compound of *Datura stramonium*.

This has been specified in the 2.2. Technical Overview of the ENPKG Workflow section in the following paragraph: "*We have notably added four links allowing to realize, for each spectrum of the ENPKG, a direct or analog spectral search against the GNPS libraries or the GNPS data index through the fasst Search interface (https://fasst.gnps2.org/fastsearch/).*"

Additionally, we have exemplified a direct spectral search for one of the features of interest discussed in the paper here: "*This feature shares 40 peaks and neutral losses with the waltherione G [M+H]+ ion and the* fasst *search against the GNPS libraries further point towards a strong spectral match with waltherione G.*"

Overall, we thank the reviewer for this suggestion. Our provided solution should answer this comment and now allows users of the ENPKG graphs to benefit from a direct spectral search against the GNPS libraries or the complete GNPS Massive datasets, both in direct or analog search mode, with a simple click.

Another aspect of the initial question of the reviewer can be understood as a suggestion to illustrate the capacities of the ENPKG workflow to handle datasets previously uploaded in the GNPS ecosystem (notably through the MassIVE repositories). It is worth noting that for metabolomics datasets to be integrated into the ENPKG, several basic requirements, notably concerning the associated metadata formatting, need to be met. These requirements are detailed here: https://github.com/enpkg/enpkg_full/tree/main/01_enpkg_data_organization. At the moment, the availability of such properly documented public datasets is limited and only a portion of the MassIVE datasets contains such information. Some properly documented datasets can however be obtained through the ReDU portal. We believe that more systematic appropriate metadata documentation will gain traction once the advantages become clearer. We hope that the current manuscript will participate in this sense.

In order to address the reviewer's suggestion we have integrated to the ENPKG, in addition to the data corresponding to the 1600 tropical plants and the Waltheria extracts profile, an additional dataset that has been profiled previously by another research team under different experimental conditions and shared publicly

(https://www.nature.com/articles/s41597-022-01662-2). This dataset consists of Xevo QToF profiles of the methanolic extracts of 337 medicinal plants from Korea.

For this, we proceeded as follows. The metadata were collected from the ReDU (https://massive.ucsd.edu/ProteoSAFe/dataset.jsp?task=1c8710f557ab4882971a1fe1045daa25) web interface, and the corresponding raw mass spectrometry profiles were downloaded from the corresponding MassIVE archive. After peak picking of the individual samples profile (focussing on the positive ionization mode), we uploaded the corresponding *.mgf* files to the MassIVE repository MSV000093464. The files required for the ENPKG workflow processing were also shared on the ENPKG Zenodo community here (https://doi.org/10.5281/zenodo.10198219).

Generic SPARQL queries can have a filter allowing users to apply them to a specific dataset identified by its MassIVE ID. By editing the value of this filter, it is possible to run the query on all datasets aggregated in the current ENPKG or only on a specific one. See L11 in SPARQL Query 1 for example. In addition, we have established specific SPARQL queries designed to illustrate the possibilities open by the ENPKG approach for heterogeneous dataset investigations. For example, Query 9 allows us to retrieve the features sharing the highest numbers of peaks and losses in their fragmentation spectrum with a given input spectrum across datasets. In addition, the query fetches the identity of the species and its upper taxonomy through a federated Wikidata query. We intended to search for plants from the Korean medicinal plants dataset that would produce features spectrally related to feature mzspec:MSV000087728:VGF151_E05_features_ms2_pos.mgf:scan:4 from an extract of the 1600 tropical plants dataset and identified as scopolamine in *Datura stramonium* (see above). The top 2 features of this search were found in extracts to *Datura metel* and *Scopolia japonica,* both from the Solanaceae family. The feature in *Datura metel* was putatively identified as scopolamine; see the corresponding fasst search in the GNPS libraries. The feature highlighted in Scopolia japonica apparently corresponds to a dihydrogenated version of scopolamine (see corresponding fasst search in the GNPS libraries).

The integration of this metabolomics dataset processed by another team and the associated SPARQL queries illustrate the power of the proposed ENPKG approach, which allows for the search of common chemistries across metabolomics datasets acquired at different times, using different platforms (Orbitrap vs. QToF), under different chromatographic conditions (8 min versus 20 min runs) and extracted using different solvents (EtOAc vs. MeOH).

The integration of this additional dataset was detailed in the manuscript.

3. The authors stress the increased importance of metadata in their method but do not address the significant difference in the quality of publicly-available metadata on various natural product collections. Some acknowledgement of the importance of properly annotated metadata (taxonomy, plant part etc.) would be helpful.

The improvement of metadata collection mechanisms is indeed fundamental. Following the reviewers' suggestion, we have developed these aspects in the manuscript.

See manuscript: "*For approaches like the ones we have presented to be more widely adopted, several aspects must be improved. First, it is essential to establish richer metadata collection mechanisms at the public mass spectrometry repository level. The ReDU framework [https://doi.org/10.1038/s41592-020-0916-7] represents a fundamental advancement in this sense.*

*Its future developments could take advantage of the progress made by initiatives such as the CEDAR workbench (https://metadatacenter.org/) and open-source toolkits such as Frictionless (https://frictionlessdata.io/) as powerful ways to standardize and facilitate metadata collection protocols. Other examples of community initiatives aiming for standardization of MS instrumental metadata or sample's metadata have been described (*https://doi.org/10.1038/s41467-023-42543-5*,* https://github.com/ERGA-consortium/ERGA-sample-manifest*) Standardizing the steps required to prepare data from the raw mass spectrometry to formats amenable to constructing a KG is also required. This standardization will require strengthening the current data processing workflow through sounder data validation processes and will require the development of simplified user interfaces to facilitate data deposition*."

4. The authors show that their system does not rely on RT and peak intensity for their analysis. Some statement on the effect of varies MS settings (i.e. fragmented voltage differences) on the MS2 data and their ability to compare datasets would help the reader.

We thank the reviewer for this question. We would, however, like to underline that the alignment approach of the ENPKG is multimodal and does not strictly rely on spectral-based alignments. Of course, such spectral-based alignments are possible (see, for example, Query 6 or Query 9) and, as shown in Query 9, it is possible to realize on MSMS spectra acquired using different platforms (in this case Orbitrap and QToF) and by consequence under different fragmentation mechanisms and energies. Here, the decomposition of individual spectra in peaks and losses offers novel spectral search possibilities. For now, we employed a spectral matching search based on the count of common peaks and losses across features. The improvements we are currently working on are based on integrating the importance order of the peaks within a given spectrum and exploiting spectral motifs (specific combinations of peaks and losses). Of course, such spectral searches depend on the richness of the compared spectra and thus on the ionization and fragmentation energies settings. These differences will inevitably impact any spectral-based comparison methods, and it is out of the scope of the current paper to enter into these considerations. Furthermore, we would also like to underline that the ENPKG approach allows for alternative alignment strategies thanks to the KG structures (using chemical structures, for example). We hope that the manuscript provides enough examples of these new ways to proceed to metabolomics profiles alignment.

5. The authors show an examples of where collecting metabolic data on 1600 samples and using their analysis methodologies increased the efficiency of determining active structured

from 6 bioactive samples. The relative efficiency of such large-scale data collection vs. the more direct analysis of only active fractions would be an informative area for the discussion section and would strengthen the manuscript.

We thank the reviewer for this comment and agree that the advantage of including data from inactive samples to interpret the screening results was not emphasized enough. We have therefore clarified the concept of biodereplication introduced in this paper and which has been followed in the presented examples.

See addition in the section (2.3.2. Specific Applications in a Drug Discovery Context) "*The transformation of large metabolomics datasets in a queryable knowledge graph structure enhanced by the connection with public electronic resources offers exciting possibilities in the frame of drug discovery research programs. Indeed the whole process can be viewed as a "virtual fractionation" of the profiled extracts, which, when applied to large extracts collections has been proven to help in the removal of common metabolic background across extracts and to efficiently highlight chemical scaffolds responsible of the observed bioactivities at the extract level, without passing by the cumbersome physical fractionation of the individual extracts [12]. Here the approach is pushed further, it leverages the precise results of the high-throughput reductionist mass-spectrometry fragmentation process (information are obtained down to the sub-molecular level in the forms of singular peaks and losses) and enhances them through a contextualization process inherent to the KG structure. The reconciliation of chemical structure identifiers allows to connect metabolite annotation results to public resource documenting the bioactivity of molecular structures (e.g. CheMBL) offering precious information for the identification of bioactive molecular structures before their physical isolation, a process we define here as biodereplication and illustrate through the following examples.*"

Regarding the specific identification of rotenoids as responsible for the activity of the 6 selected extracts, this assumption was strengthened by the fact that while fatty acids were also shared among the active extracts, only the rotenoids were specific to them. Such conclusions could not have been made if the whole dataset (including inactive extract) wouldn't have been profiled and considered as a whole.

To highlight this, we modified the manuscript accordingly and added this clarification: "*[...]. It is important here to highlight the interest in integrating data from inactive samples in the analysis. By looking only at common annotations in active extracts, rotenoids and fatty acids derivatives are highlighted (Figure 3 B, first TMAP from the left). However, by looking at the specificity of these compounds, we can observe that only the rotenoids are specific to the cluster of active extracts, while fatty acids are spread among a large number of samples and thus less likely to be responsible for the activity (Figure 3 B, second TMAP from the left).*"

In addition, this sample set of 1,600 tropical extracts is also assayed on other targets in current and future projects (including 4 pro-metabolic readouts and anti-infective bioassays). In this respect, integrating these multiple bioassay results, even negative ones, allows to infer the properties of some extracts and their content, for example, to spot generally cytotoxic extracts and molecules. While a

general comparison of the efficiency of the different methods available is difficult in practice and greatly project-dependent, every experimental result is in our view equally important and should therefore be made available to the community.

Finally we have specified in the manuscript that: "*We would like to underline that if we have here incorporated ChEMBL datasets relevant to our current drug discovery objectives (i.e. anti.trypanosomatids), there are basically no limits to the incorporation of other bioactivity datasets comprised of chemical structure and their evaluated bioactivity on any given target — provided that these are shared publicly. This opens exciting possibilities as it becomes possible to detect spectrally related analogues of compounds previously bio-assayed by others, hence opening the possibility to fish potential bioactives from complex extracts library* without *even realizing the initial screening campaigns.*"

We hope that these additions to the manuscript clarify the interest of building large knowledge graphs from experimental metabolomics outputs in the frame of drug discovery projects.

6. On pg. 12, the authors discuss how the a feature sharing the highest number of peaks and losses became "feature #1" of an active extract of Melochia umbellata. Would such a prioritization strategy be indicative of the relative abundance of a feature in an extract (an alternative measure similar to peak intensity for relative quantification of a feature to associate with bioactivity?

Here, we have rephrased the sentence, including the mention of "feature #1" to "feature id 1" and have added a link to the URL of the given feature inside the ENPKG (https://enpkg.commons-lab.org/graphdb/resource?uri=https:%2F%2Fenpkg.commons-lab.org%2Fkg%2Flcms_feature_mzspec:MSV000087728:VGF156_A06_features_ms2_pos.mgf:scan:1&role=subject). Indeed the formulation could be misleading, and here, there has been no re-ranking of the feature; it just turned out that the feature displaying the highest number of common peaks and losses with the waltherione G spectra was a feature with id number 1 in the *Melochia* extract. In the presented query, while this is available information within the KG, we are not considering peak intensity. Indeed, we believe that the batch effects are too important for datasets acquired on several platforms to exploit such a dimension and link it to the detected bioactivities.

7. The authors state that retention time is not important for their methodology yes in Table 1 Query #7 &8 are both dependent on retention time data. Please explain.

We thank the reviewer for this observation. In the ENPKG approach, we indeed proceed to alignment using dimensions other than the ones classically used in metabolomics, namely retention time and *m/z* ratio. This allows us to proceed to the alignment of largely heterogeneous datasets acquired under different experimental conditions (see query 9). However, this does not mean we do not exploit such dimensions for other purposes. For example, in queries 7 and 8, we use the retention time tolerance because we are comparing profiles of extracts acquired under the same Liquid Chromatography conditions (in this case, across a positive ionization and negative ionization mode). Retention time,

despite not being used for the alignment of all samples of the ENPKG datasets, is still valuable information. We can, for example, knowing the chromatographic condition of a given run, exploit the RT information to judge the pertinence of an annotation (e.g. fatty acid derivatives being annotated toward the end of a chromatographic run in reverse phase).

8. The authors on pg. 16 use the unit micrograms/mL for specific compounds and later use molar units for other compounds. Molar units should be used for all compounds for ease of comparison.

We thank the reviewer for this suggestion. We previously reported the activity for some compounds in micrograms/mL since this was the unit used in the original paper describing the observed bioactivity. To ease the comparison with other activity values, we added the corresponding concentration in μM.

Overall the paper is interesting and potentially helpful to the natural products research community. I recommend publishing after revision.

Additional Questions:
Element of innovation: High

Impact on the field at large: High

Strength of authors plan to promote uptake and dissemination of the work: High

Does the work address an important, unmet need in the chemical community?: Yes

If yes, how important is the unmet need?: Very important

Is this research study suitable for media coverage or a First Reactions (a News & Views piece in the journal)?: No

Reviewer: 2

Recommendation: Reconsider after major revisions noted.

Comments:
Review of oc-2023-000800h

This is an interesting and innovative paper describing the application of knowledge graph methods to questions in natural products. These approaches have not seen large-scale adoption by this community, and it will therefore be of broad interest to that readership. Use of knowledge graphs is an emerging strategy for large-scale data integration in other fields of biomedical science. This paper brings value by not only demonstrating applications of the methodology for antiparasitic compound discovery, but also providing a computational

framework to generate KGs using toolsets available to the NP community. Notwithstanding the benefits, I have several issues that should be addressed.

1) Positioning of selected tools. The paper uses a suite of annotation tools for NP discovery that offer one solution to problems of annotation and identification of untargeted MS datasets. However the text is written to imply that these are the only solutions to such problems, which is not correct. The early sections of the paper need to be expanded to place this workflow in context with the rest of the field. MZMine is not the only MS processing software package, NP Classifier is not the only chemical ontology etc.

We thank the reviewer for this comment. Indeed the current ENPKG workflow is tailored to treat the computational mass spectrometry tools that are typically used within our labs and we are perfectly aware that other valuable solutions exist. We have not found a formulation in the original manuscript implying that these were the only solutions for peak-picking or metabolite annotation, yet we thank the reviewer for his comment which prompted us to discuss these topics in the possible optimizations of the designed approach. Extending the capacities of the ENPKG workflow to handle the outputs of other popular or less known software of the metabolomics toolbox would indeed be an improvement. We have mentioned this explicitly in section *2.4. Overview, current limitations & future improvements*.

See added paragraph: "*We would like to underline that we have here employed a series of tools and strategies commonly used in our labs for the peak-picking (i.e. MzMine) and metabolite annotation stage (i.e. Sirius, taxonomically informed metabolite annotation using the ISDB-LOTUS). These tools are however not the only ones available and the ENPKG workflow should be adapted in the future to better handle the output of other peak-picking (e.g. XCMS or asari), metabolite annotation strategies (e.g. MSDial) or chemical taxonomies (e.g. ChemOnt from ClassyFire).*"

2) Demonstration of utility of KG methodology. I found the two examples provided to be interesting, but I was not convinced that these results would have been that difficult to extract without KG approaches. "Which extracts are active against target X with low cytotoxicity against Y" could easily be accomplished by filtering in Excel. The second stage, where SIRIUS annotations are counted and examined (Fig 3B) is more complicated, but still not that difficult to do by collating and sorting results files for individual samples. I appreciate that this approach is scalable and flexible, but as written the examples do not present a step change in search ability or knowledge generation compared to existing analogue methods. The authors should consider additional information that could be derived from the graph. For example, does the general rotenone scaffold have precedent as an antiparasitic agent against other kinetoplastid parasites? Are any of those compounds present in the active extract set? Or other samples in the NP library?

We thank the reviewer for his comment. Indeed, within Table 1, the first SPARQL queries have been designed for pedagogic purposes (e.g. SPARQL 1 and 2). In the text we also present basic SPARQL queries such as a simple feature count. We agree that these examples could be obtained using other tools, for example, tables join operations or filtering, eventually achievable using Excel. However, the power of the KG integration lies in the possibility of querying and

combining mixed data types (spectral, taxonomical, bioactivity, etc.) using a single technology and from a single platform. In this respect, other presented queries, such as Query #5 from Table 1, illustrates this combination: it integrates spectral annotations, Wikidata-hosted structure-organism pair information, and ChEMBL biological activity data. While the same results could be obtained through individual queries of each of these resources, the possibility of combining them in a single query on the KG greatly simplifies and streamlines this work. Such complex requests are not achievable through simple Excel searches.

Regarding the rotenoid activity example (section 2.3.2.1. Identification of Potent Anti-Trypanosoma cruzi Agents), and following the reviewer's suggestion, we have added a supplementary query and modified the manuscript to highlight that among annotated compounds in the cluster of active extracts, no rotenoid has an activity previously reported against *T. cruzi*, *T. brucei* or *L. donovani*. The other questions (*Are any of those compounds present in the active extract set? Or other samples in the NP library?*) are answered through the queries and graphical displays of section B) in Figure 3.

Added sentence: "*In addition, a search of ChEMBL reported anti-T. cruzi, T. brucei or L. donovani activity among annotated compounds in these extracts returns eight distinct compounds but no rotenoids, confirming the potentially new activity of this class of compounds against T. cruzi (query).*"

3) Accessibility for end users. Most NP chemists are not computer science experts. Although the paper is accompanied by a GitHub repository that includes wrappers for running each of the required tools and instructions about data formatting etc. this is still a manual process that requires users to install and run each step in series. Adoption would be much higher if these steps were wrapped into a single package (e.g. using Gooey) that gave end users an easier interface. This would be particularly valuable if packaged as a stand-alone app for both Windows and Mac. This is clearly significant additional work, but not that complicated given that most individual steps are already single command line arguments. This addition would improve standardization and could dramatically increase community adoption.

We thank the reviewer for this suggestion. We know that for such approaches to benefit from a broader adoption by the community, it will be required to develop tools facilitating data formatting and preparation. The establishment of a standalone GUI is, however, out of the scope of the current paper, which is rather designed as a proof of concept regarding the application of Linked Open Data formatting for the organization of large metabolomics datasets. We have, however, followed the reviewer's suggestion and provided a solidified command line interface allowing interested users to run all the steps of the overall ENPKG workflow (see https://github.com/enpkg/enpkg_full). Furthermore, we established a toy dataset consisting of three samples and the required data and metadata. This example dataset is available here: https://doi.org/10.5281/zenodo.10016507.

In addition, in the frame of our current research projects and to facilitate wider adoption of the proposed workflow by possible contributors, we are working on the establishment of a web interface that will be able to digest a compressed archive provided by the user, proceed to all of the steps described in the ENPKG workflow and return a set of files which can directly be viewed and exploited in software suites such as GraphDB. This interface is under active development but can be observed and used by the reviewer at https://kg.earthmetabolome.org. For this the .zip of the example dataset obtained at https://doi.org/10.5281/zenodo.10016507 can be simply dragged and dropped after ORCID login to the portal.

Additional Questions:
Element of innovation: Top 5%

Impact on the field at large: High

Strength of authors plan to promote uptake and dissemination of the work: Top 5%

Does the work address an important, unmet need in the chemical community?: Yes

If yes, how important is the unmet need?: Very important

Is this research study suitable for media coverage or a First Reactions (a News & Views piece in the journal)?: No

Reviewer: 3

Recommendation: Publish in ACS Central Science without change.

Comments:
This paper by Gaudry et al describes their approach to processing and interpreting LC-MS data for natural product research. A key part of their workflow is the removal of the feature alignment step that is widely used for analysis of large datasets in order to allow each sample to be considered individually in the data analysis instead of trying to compare entire feature lists from different datasets. The authors have proposed a brilliant way to utilize the amount of data that is publicly available and yet extremely underutilized due to the limitations that they point out. They even went so far as to prove how it can be used to identify new natural products and have performed purification and structure elucidation of several compounds, which is no small feat. It is difficult to find fault in this work and the only comment is that it will be highly impactful to the field only if they can address the main drawback to their approach, namely the high level of expertise needed to implement the proposed workflow and by extension the inaccessibility to the average researcher. The authors do seem to acknowledge this, and this reviewer hopes they continue to pursue the development of an accessible user interface.

1. Section 2.3.1: Are the computer specs necessary for such a big dataset workflow prohibitory? It seems like it would be difficult to get MZmine to process so much data and then work with a list of > 1 million features and 800,000 in silico annotations on a typical data analysis computer. How long did it take to process this data and is this type of analysis possible for most LC-MS users? Or did they run a single sample through the MZmine workflow at a time?

The reviewer is correct with the last proposition. Indeed, one of the significant advantages of the proposed workflow is the sample-centric data treatment. With the alignment established via the KG structure, there is no need to proceed to an alignment step in MZMine. This alignment step can be computationally demanding, notably because it cannot be parallelized. To resume, the MZmine processing of the current dataset (1920 runs) for the ENPKG can be achieved on a standard laptop. On the other hand, it is true that bypassing the RT-based alignment highly increases the number of spectral features to be annotated in a second step (in this case, passing from ~120,000 to > 700,000 features for the PI mode of the 1,600 plant extracts dataset, see https://academic.oup.com/gigascience/article/doi/10.1093/gigascience/giac124/6980761 for the aligned data). This implies a higher number of annotations to be done and, therefore, a higher computational cost. However, as samples only have to be processed once to be included in a KG, we believe this initial investment pays for itself in the form of easily reusable data.

2. If retention time information is not used to align features can the authors comment on whether they think the mzmine processing and feature networking is necessary/superior to a classical molecular networking workflow where mzmine processing is not used and really only MS2 patterns are used to create networks? Or do they view them as similarly effective?

If we do not realize the RT-based alignment step in our workflow, we strongly believe in the utmost importance of applying a peak-picking stage in order to exploit the separative power of the LC system. Hence, we view FBMN approaches as superior to classical MN approaches. The possibilities to 1) resolve isomers and 2) conserve accurate mass information are crucial. Furthermore, if RT is not a dimension used for large-scale alignment in our approach, it is still a perfectly valid source of information that can help in the structural determination process. For example, the annotation of highly polar compounds, such as polyoses, at the end of an LC run on a C18 column is dubious.

3. Again, the main critique is that it would take an experience untargeted metabolomics scientist an extensive amount of time to learn how to implement this framework. Do they have plans for a user platform or integration into large platforms like GNPS?

We thank the reviewer for this suggestion. It has also been highlighted as a point to improve by another reviewer. Here is our response:

We thank the reviewer for this suggestion. We know that for such approaches to benefit from a broader adoption by the community, it will be required to develop tools facilitating data formatting

and preparation. The establishment of a standalone GUI is, however, out of the scope of the current paper, which is rather designed as a proof of concept of applying Linked Open Data formatting for the organization of large metabolomics datasets.

We have, however, followed the reviewer's suggestion and provided a solidified command line interface allowing interested users to run all the steps of the overall ENPKG workflow (see https://github.com/enpkg/enpkg_full). Furthermore, we established a toy dataset consisting of three samples and the required data and metadata. This example dataset is available here: https://doi.org/10.5281/zenodo.10016507.

In addition, in the frame of our current research projects and to facilitate wider adoption of the proposed workflow by possible contributors, we are working on the establishment of a web interface that will be able to digest a compressed archive provided by the user, proceed to all of the steps described in the ENPKG workflow and return a set of files which can directly be viewed and exploited in software suites such as GraphDB. This interface is under active development but can be observed and used by the reviewer at https://kg.earthmetabolome.org. For this the .zip of the example dataset obtained at https://doi.org/10.5281/zenodo.10016507 can be simply dragged and dropped after ORCID login to the portal.

Additional Questions:
Element of innovation: High

Impact on the field at large: High

Strength of authors plan to promote uptake and dissemination of the work: Top 5%

Does the work address an important, unmet need in the chemical community?: Yes

If yes, how important is the unmet need?: Very important

Is this research study suitable for media coverage or a First Reactions (a News & Views piece in the journal)?: Yes

oc-2023-00800h.R2

Name: Peer Review Information for "A Sample-Centric and Knowledge-Driven Computational Framework for Natural Products Drug Discovery"

Second Round of Reviewer Comments

Reviewer: 3

Comments to the Author

The authors have addressed all comments and provided the clarification necessary for publication.

Reviewer: 2

Comments to the Author

This revision was not easy to review as a marked copy of the revised manuscript was not provided. The authors addressed my comments about placing this work in the broader context of NP-based metabolomics workflows by adding two sentences to section 2.4. This has made it clearer that the approach will only work with the specified tools, and has also improved recognition of alternative (unsupported) methods for data processing in this area.

Secondly, the authors have created a new repo in the GitHub project (enpkg_full) that provides a wrapper to run the set of individual steps that are part of the enpkg workflow. While this is a welcome addition, the instructions for this new tool are still very sparse. The full README includes fewer than 30 lines of content and is far too brief to be of practical use to non-specialists. Therefore, my original concern that this package is not accessible to the general NP community remains. There are additional instructions but these are buried as multi-line comments in enpkg_full/tests/test_data_organization.py and are not very informative. For example, lines 17 – 20 read:

"# XCMS pipeline for peak picking

  \#   - Problem with the XCMS pipeline:

  \#   - The files for the Earth metabolome are meant to be from several different machines, and therefore the parameters such as the noise would need to be adjusted for each machine.

  \# For the time being we will assume that the processed data are

  \# provided by a machine expert user."

The authors state twice in the response to reviewers that creating a user interface is outside the scope of this study, but without minimally instructive documentation this pipeline will remain inaccessible to most readers. The authors either need to create a functional and well-tested UI, or they need to create appropriate documentation to allow naïve users to install, launch and use this new pipeline. Many academic tools provide this level of documentation. Examples include GNPS (https://ccms-

ucsd.github.io/GNPSDocumentation/quickstart/), SIRIUS (https://boecker-lab.github.io/docs.sirius.github.io/), SMART NMR (https://mwang87.github.io/SMART_NMR_Documentation/smart_usage/) and SNAP-MS (https://liningtonlab.github.io/snapms_documentation/).

Reviewer: 1

Comments to the Author

Gaudy and co-authors have submitted an improved revised manuscript which conscientiously addressed all of this reviewer's previous comments. The additional proofs of utility for their method have strengthened the manuscript. I encourage acceptance without further revision.

Author's Response to Peer Review Comments:

Dear Prof. Editor,

We thank you and the three reviewers for their feedback on this second round of reviews which has been crucial to improve the quality of the manuscript and the code. We sincerely hope that the answer we are now bringing (bold blue in the attached document) satisfy Rev 2.

We are working hard on the development of a web portal to democratize the use of semantic web technologies in the field of metabolomics. The development of such a portal will take time but we are convinced that the current seminal paper constitutes a first step towards the adoption of such approaches and hold the potential to deeply transform the field and help in better characterizing the chemodiversity of living systems. We believe it will be of great interest to readers of ACS Central Science.

With our best regards,

Pierre-Marie Allard - on the behalf of the ENPKG authors

08-Jan-2024

Journal: ACS Central Science

Manuscript ID: oc-2023-00800h.R1

Original Submission Date: 30-Jun-2023 Title: "A Sample-Centric and Knowledge-Driven Computational Framework for Natural

Products Drug Discovery"

Author(s): Gaudry, Arnaud; Pagni, Marco; Mehl, Florence; Moretti, Sébastien; QuirósGuerrero, Luis; Cappelletti, Luca; Rutz, Adriano; Kaiser, Marcel; Marcourt, Laurence; Queiroz, Emerson; Ioset, Jean-Robert; Grondin, Antonio; David, Bruno; Wolfender, Jean-Luc; Allard,

Pierre-Marie

Dear Dr. Allard:

Thank you for your recent submission to ACS Central Science. We have now received the reviews for your manuscript and I am pleased to inform you they were quite positive.

However, the reviewers have raised important points that require attention and must be addressed before a final decision can be made.

Please make the appropriate changes to your manuscript and submit a revised manuscript no later than 22-Jan-2024. Your manuscript may be subject to further peer review but if the revision can thoroughly address the outstanding concerns, we aim to minimize further backand-forth correspondence between authors and referees and make editorial decisions in house, which will save time and effort for all and expedite processing of your paper.

The revision should address the reviewers' comments and include a point-by-point response. In addition to a clean copy of the revised manuscript, please also submit a tracked version of the original submission that shows the actual changes (deletions and additions) made to the manuscript. You may highlight, color font, or underline the changes.

ACS Central Science offers authors an opportunity to participate in transparent peer review. Transparent peer review allows the reader to see the exchange between authors and reviewers. Transparent peer review allows the reader to see the exchange between authors and reviewers. If an author chooses to participate in transparent peer review, the anonymous reviewers' comments and author's response to the reviewers will be published as supporting information if the manuscript is accepted for publication. More information about transparent peer review can be found here https://pubs.acs.org/page/peer_reviews/transparent_peer_review.html or at a recently published editorial https://pubs.acs.org/doi/10.1021/acscentsci.1c01238.

During submission, you were given a choice to participate in transparent peer review. You responded as follows:

Yes, I will participate in transparent peer review.

If you opted to participate in transparent peer review, you can change your mind at any revision stage. If you have questions about transparent peer review not answered in our FAQs, please contact ACS Publications Support at support@services.acs.org or contact the editorial office.

Prior to submitting your revision, please also be sure to address the formatting issues listed above the reviewer comments. Further information regarding press, hero images, etc. is included in the attached 'Author Checklist' document. Please note that certain non-scientific needs are required prior to acceptance.

Funding Sources: Authors are required to report ALL funding sources and grant/award numbers relevant to this manuscript. Enter all sources of funding for ALL authors relevant to this manuscript in BOTH the Open Funder Registry tool in ACS Paragon Plus and in the manuscript to meet this requirement. See http://pubs.acs.org/page/4authors/funder_options.html for complete instructions.

ORCID: Authors submitting manuscript revisions are required to provide their own validated ORCID iDs before completing the submission, if an ORCID iD is not already associated with their ACS Paragon Plus user profiles. This iD may be provided during original manuscript submission or when submitting the manuscript revision. You can provide only your own ORCID iD, a unique researcher identifier. If your ORCID iD is not already validated and associated with your ACS Paragon Plus user profile, you may do so by following the ORCID-related links in the Email/Name section of your ACS Paragon Plus account. All authors are encouraged to register for and associate their own ORCID iDs with their ACS Paragon Plus profiles. The ORCID iD will be displayed in the published article for any author on a manuscript who has a validated ORCID iD associated with ACS Paragon Plus when the manuscript is accepted. Learn more at http://www.orcid.org.

The guidelines for submitting (1) the revised manuscript, (2) responses to reviewer concerns, and (3) the annotated manuscript are provided below.

(1) To submit the revised version, log into ACS Paragon Plus with your ACS ID at http://acsparagonplus.acs.org/ and select "My Authoring Activity." There you will find your manuscript title listed under "Revisions Requested by Editorial Office." With the exception of your main text file, all of your original files will be available to you for review or replacement during the revision process. If you need to replace a file, please be sure to remove the original before uploading a new one. Please note that you must upload a new, revised manuscript file.

(2) The ACS Paragon Plus system also allows you to respond to the comments made by the reviewer(s) either in the text box provided or by attaching a file containing your detailed responses to all of the points raised by the reviewers.

(3) In addition to uploading your revised manuscript file, please also upload an annotated copy of the manuscript that tracks deletions and additions for the benefit of the reviewers and editor. This marked-up manuscript should be uploaded electronically in the File Upload section as "Supporting Information for Review Only".

ACS Central Science is a diamond open access journal and publishes exclusively using a Creative Commons Attribution license (CC-BY) license. If your article is accepted in ACS Central Science, your article will be published under a CC-BY license at no charge.

ACS Publications uses CrossCheck's iThenticate software to detect instances of similarity in submitted manuscripts. In publishing only original research, ACS is committed to deterring plagiarism, including self-plagiarism. Your manuscript may be screened for similarity to published material.

We look forward to receiving your revised manuscript.

Best wishes,

Prof. Editor

ACS Central Science

Deputy Editor Email:

Formatting Needs:

AU EMAIL: Please label as email

GOOGLE SCHOLAR ID: Please label

ABSTRACT: Please remove, the Significance Statement will take its place

REFERENCES: Please move to the last page of the manuscript

TOC: Please move to the last page of the manuscript, beneath the References.

SI PG#S: The supporting information pages must be numbered consecutively, starting with page S1.

Reviewer(s)' Comments to Author:

Reviewer: 3

Recommendation: Publish in ACS Central Science without change.

Comments:

The authors have addressed all comments and provided the clarification necessary for publication.

Additional Questions:

Element of innovation: High

Impact on the field at large: High

Strength of authors plan to promote uptake and dissemination of the work: Top 5%

Does the work address an important, unmet need in the chemical community?: Yes

If yes, how important is the unmet need?: Very important

Is this research study suitable for media coverage or a First Reactions (a News & Views piece in the journal)?: Yes

Reviewer: 2

Recommendation: Publish in ACS Central Science after minor revisions noted.

Comments:

This revision was not easy to review as a marked copy of the revised manuscript was not provided. The authors addressed my comments about placing this work in the broader context of NP-based metabolomics workflows by adding two sentences to section 2.4. This has made it clearer that the approach will only work with the specified tools, and has also improved recognition of alternative (unsupported) methods for data processing in this area.

We thank the reviewer for his appreciation. The manuscript with track of changes was submitted in the last revision (file called A Sample-Centric and Knowledge-Driven Computational Framework for Natural Products Drug Discovery_Track_Of_Changes.docx). This time we also submit two files for the reviewer to keep track of changes. The first one keeps track of changes between the initial submission and the current version (A SampleCentric and Knowledge-Driven Computational Framework for Natural Products Drug Discovery_TOC_vs_initial_submission.docx) the second one keeps track of changes between the first resubmission and the current one (A Sample-Centric and Knowledge-Driven Computational Framework for Natural Products Drug Discovery_TOC_vs_initial_resubmission.docx).

Secondly, the authors have created a new repo in the GitHub project (enpkg_full) that provides a wrapper to run the set of individual steps that are part of the enpkg workflow. While this is a welcome addition, the instructions for this new tool are still very sparse. The full README includes fewer than 30 lines of content and is far too brief to be of practical use to nonspecialists. Therefore, my original concern that this package is not accessible to the general NP community remains.

We thank the reviewer for his appreciation. However, we do not agree that longer README means better README. The current README has been clarified. https://github.com/enpkg/enpkg_full It should allow user to fully run the enpkg_full pipeline. We have specified unified the parameters settings in a single .yaml file. Furthermore, the toy dataset (downloadable directly vie the enpkg_full commands) allows users to replicate the pipeline on a minimal example. We have specified and ecouraged question through issue mechanism on the github repository. We are inviting the reviewer to directly post issues and / or edits of the README if this one would prove to be insufficient to run the enpkg_full pipeline.

There are additional instructions but these are buried as multi-line comments in enpkg_full/tests/test_data_organization.py and are not very informative. For example, lines 17 – 20 read:

“# XCMS pipeline for peak picking

#   - Problem with the XCMS pipeline: #   - The files for the Earth metabolome are meant to be from several different machines, and therefore the parameters such as the noise would need to be adjusted for each machine.

For the time being we will assume that the processed data are

provided by a machine expert user."

Many thanks for this observation. These lines were indeed corresponding to reliques of the initial development of the enpkg_full pipeline. We have removed them.

The authors state twice in the response to reviewers that creating a user interface is outside the scope of this study, but without minimally instructive documentation this pipeline will remain inaccessible to most readers. The authors either need to create a functional and welltested UI, or they need to create appropriate documentation to allow naïve users to install, launch and use this new pipeline. Many academic tools provide this level of documentation. Examples include GNPS (https://ccms-ucsd.github.io/GNPSDocumentation/quickstart/), SIRIUS (https://boecker-lab.github.io/docs.sirius.github.io/), SMART NMR (https://mwang87.github.io/SMART_NMR_Documentation/smart_usage/) and SNAP-MS (https://liningtonlab.github.io/snapms_documentation/).

We thank the reviewer for his insistence on this important point. We still maintain that the development of a standalone web interface is completely out of the scope of the current paper which had for objective to showcase, with reusable and reproducible code the transformation of untargeted metabolomics datasets in Linked Open Data format. The examples provided by the reviewer (e.g. GNPS, SIRIUS) have 10+ years of dedicated development. We however do fully agree that such interfaces are required for a better adoption by the community. For this, as stated in the initial resubmission we have, thanks to the comments of the reviewers, developed a prototype of a web interface which is accessible to users after ORCID login at https://kg.earthmetabolome.org and already allows to use a test example, drag and drop it on the interface and returns a set of .ttl files usable in a graph management software such as GraphDB. This portal is currently under active development and will be entirely coded as a Rust monolith with API for Python modules.

This will require a dedicated datascientist and a postdoc of our lab in the next year. The code is fully public at https://github.com/earth-metabolome-initiative/emikg

Additional Questions:

Element of innovation: Top 5%

Impact on the field at large: Top 5%

Strength of authors plan to promote uptake and dissemination of the work: Moderate

Does the work address an important, unmet need in the chemical community?: Yes

If yes, how important is the unmet need?: Very important

Is this research study suitable for media coverage or a First Reactions (a News & Views piece in the journal)?: No

Reviewer: 1

Recommendation: Publish in ACS Central Science without change.

Comments:

Gaudy and co-authors have submitted an improved revised manuscript which conscientiously addressed all of this reviewer's previous comments. The additional proofs of utility for their method have strengthened the manuscript. I encourage acceptance without further revision.

Additional Questions:

Element of innovation: High

Impact on the field at large: Moderate

Strength of authors plan to promote uptake and dissemination of the work: High

Does the work address an important, unmet need in the chemical community?: Yes

If yes, how important is the unmet need?: Very important

Is this research study suitable for media coverage or a First Reactions (a News & Views piece in the journal)?: No

----

FOR ASSISTANCE WITH YOUR MANUSCRIPT SUBMISSION PLEASE CONTACT:
ACS Publications Customer Services & Information (CSI)
Email: support@services.acs.org Phone: 202-872-4357 Toll-Free Phone: 800-227-9919 (USA/Canada only)

PLEASE NOTE: This email message, including any attachments, contains confidential information related to peer review and is intended solely for the personal use of the recipient(s) named above. No part of this communication or any related attachments may be shared with or disclosed to any third party or organization without the explicit prior written consent of the journal Editor and ACS. If the reader of this message is not the intended recipient or is not responsible for delivering it to the intended recipient, you have received this communication in error. Please notify the sender immediately by e-mail, and delete the original message.

As an author or reviewer for ACS Publications, we may send you communications about related journals, topics or products and services from the American Chemical Society. Please email us at pubs-comms-unsub@acs.org if you do not want to receive these. Note, you will still receive updates about your manuscripts, reviews, or future invitations to review.

Thank you.
